# Essential role of Cp190 in physical and regulatory boundary formation

**DOI:** 10.1126/sciadv.abl8834

**Published:** 2022-05-13

**Authors:** Anjali Kaushal, Julien Dorier, Bihan Wang, Giriram Mohana, Michael Taschner, Pascal Cousin, Patrice Waridel, Christian Iseli, Anastasiia Semenova, Simon Restrepo, Nicolas Guex, Erez Lieberman Aiden, Maria Cristina Gambetta

**Affiliations:** 1 Center for Integrative Genomics, University of Lausanne, 1015 Lausanne, Switzerland.; 2 Bioinformatics Competence Center, University of Lausanne, 1015 Lausanne, Switzerland.; 3 Department of Fundamental Microbiology, University of Lausanne, 1015 Lausanne, Switzerland.; 4 Protein Analysis Facility, University of Lausanne, 1015 Lausanne, Switzerland.; 5 arcoris bio AG, Lüssirainstrasse 52, 6300 Zug, Switzerland.; 6 The Center for Genome Architecture, Baylor College of Medicine, Houston, TX 77030, USA.; 7 National Institute of Genetics, 1111 Yaya, Mishima, Shizuoka 411-8540, Japan.; 8 UWA School of Agriculture and Environment, The University of Western Australia, Perth, WA 6009, Australia.; 9 Shanghai Institute for Advanced Immunochemical Studies, ShanghaiTech, Pudong 20120, China.

## Abstract

Boundaries in animal genomes delimit contact domains with enhanced internal contact frequencies and have debated functions in limiting regulatory cross-talk between domains and guiding enhancers to target promoters. Most mammalian boundaries form by stalling of chromosomal loop-extruding cohesin by CTCF, but most *Drosophila* boundaries form CTCF independently. However, how CTCF-independent boundaries form and function remains largely unexplored. Here, we assess genome folding and developmental gene expression in fly embryos lacking the ubiquitous boundary-associated factor Cp190. We find that sequence-specific DNA binding proteins such as CTCF and Su(Hw) directly interact with and recruit Cp190 to form most promoter-distal boundaries. Cp190 is essential for early development and prevents regulatory cross-talk between specific gene loci that pattern the embryo. Cp190 was, in contrast, dispensable for long-range enhancer-promoter communication at tested loci. Cp190 is thus currently the major player in fly boundary formation and function, revealing that diverse mechanisms evolved to partition genomes into independent regulatory domains.

## Teaser

Fly Cp190 is an essential adaptor protein required to form contact domain boundaries and prevent regulatory cross-talk.

## INTRODUCTION

Chromosomal contact domains are ubiquitous in different species, but there is evidence that they form through diverse mechanisms. Two fundamental questions are as follows: How are contact domains formed, and what is their function? Contact domains are known to form through compartmentalization of transcriptionally active and inactive domains or extrusion of chromosomal loops by cohesin until cohesin is stalled by DNA-bound CTCF at domain boundaries (*1*–*4*). Contact domains arising from these different mechanisms have respectively been dubbed “compartmental domains” or “topologically associating domains” (TADs) (*1*, *5*, *6*). CTCF is required to form a large proportion of mammalian contact domain boundaries but less than 10% of all boundaries in *Drosophila* (*7*). In *Drosophila*, three-quarters of contact domain boundaries are located at promoters of highly and ubiquitously expressed genes and hence called promoter boundaries, while the remaining one-quarter are nonpromoter boundaries occupied by different DNA binding proteins such as CTCF or Su(Hw) (suppressor of Hairy-Wing) (*8*). Promoter and nonpromoter boundaries in flies all share a common feature: They are bound by Centrosomal protein 190 kDa (Cp190) (*7*, *8*). The diversity of boundary-associated factors in flies compared to mammals raises the possibility that additional proteins other than CTCF form physical boundaries in chromosomes through yet unknown mechanisms.

How genome folding into contact domains affects gene regulation has been intensely investigated by studying mammalian CTCF. A major challenge is that CTCF is essential for mammalian cell survival, and acute CTCF depletion results in few transcriptional effects (*2*). Locus-specific CTCF-dependent boundary perturbations led to different conclusions on their relevance for gene regulation (*9*–*13*). Contact domains generally contain co-regulated genes and their cognate regulatory elements (*8*, *14*, *15*) and, in some cases, foster efficient activation of promoters by enhancers within the same domain (*16*–*18*). Conversely, contact domain boundaries can exert genetic insulator activity by strongly dampening communication between regulatory elements and gene promoters in flanking domains, in exceptional cases preventing developmental defects and human disease (*18*–*20*). An emerging model in mammals is that cohesin-mediated loop extrusion brings enhancers into functional contact with compatible promoters all the way until cohesin is stalled by CTCF (*21*, *22*).

In contrast to mammals, CTCF has a limited role at selected boundaries in *Drosophila* (*7*). Despite the presence of Cp190 at nearly all boundaries, studies have not yet addressed whether Cp190 is critical for gene regulation specificity. It can seem puzzling that Cp190 associates with very different types of boundaries (promoter and nonpromoter), and it remains unclear whether it exerts different activities at these sites (*23*, *24*).

Cp190 was identified in a genetic screen as essential for the activity of the well-characterized *gypsy* insulator and was later shown to be required at additional insulators (*25*–*27*). It remains, however, unclear how relevant Cp190 is for natural gene expression (*7*, *28*–*30*). Cp190 copurifies with diverse proteins, indicating assembly into complexes whose exact compositions remain unclear because it is challenging to assemble them recombinantly. Several Cp190 interactors exert insulator activity in transgenic reporter assays, suggesting that Cp190 is an essential insulator cofactor (*31*, *32*). Cp190 is recruited to chromosomes by sequence-specific DNA binding insulator proteins (*7*, *26*, *27*, *30*). For example, CTCF recruits Cp190 to CTCF-dependent boundaries (*7*). In *CTCF^0^* mutants, residual Cp190 binding was observed at some former CTCF peaks, and this importantly correlated with boundary retention (*7*). This had raised the possibility that Cp190 synergizes with CTCF to form contact domain boundaries.

Apart from limiting regulatory cross-talk, some Cp190-bound insulators were shown to physically pair and thereby bring linked promoters and distal regulatory elements into close proximity to enable long-range regulation (*33*–*35*). In extreme examples, physical pairing of insulators enabled regulation of a promoter by an enhancer 140 kb away or by an enhancer located on the homologous chromosome (*36*–*38*). Observations from seminal studies led to the influential model in which DNA-bound insulator proteins pair through Cp190 acting as a universal glue, for example by dimerizing through its BTB (Broad-Complex, Tramtrack and Bric a brac) domain (*29*, *33*, *39*, *40*).

Here, we directly address the biological relevance of the major fly boundary-associated factor Cp190. We performed Hi-C, Capture-C, and chromatin immunoprecipitation sequencing (ChIP-seq) in *Cp190^0^* mutants completely lacking Cp190 to uncover that Cp190 is required to form most nonpromoter boundaries. Promoter boundaries are insensitive to Cp190 loss and are thus formed through separate mechanisms. By optimizing our genetic strategy to generate not only *Cp190^0^* and *CTCF^0^* single mutants but also *double^0^* mutants lacking both CTCF and Cp190 products combined, we demonstrate that Cp190 is required for DNA-bound CTCF to form a robust boundary. We show that Cp190 associates with various insulator proteins in the context of core complexes with shared subunits. We quantify the relative enhancer-blocking activities of these complexes in a reporter assay to assess whether promoter and nonpromoter boundaries have different insulator activities. Last, by exploring how gene regulation is affected at well-characterized developmental loci, we were able to clearly assess the relevance of widespread contact domain boundary impairment for both inhibition and facilitation of enhancer-promoter communication at these loci during embryogenesis. In *Cp190^0^* mutants, all tested developmental genes are expressed in their endogenous and, in some cases, additional ectopic patterns in a manner consistent with characterized enhancers in flanking contact domains. We reveal that Cp190 is critical for the ability of classical developmental gene boundaries that we tested to block enhancers but not to mediate long-range gene activation by distal enhancers located in another contact domain. These findings demonstrate that diverse mechanisms exist to fold genomes into independent gene regulatory domains beyond what is currently known in mammals and refine our understanding of how fly contact domain boundaries affect gene regulation.

## RESULTS

### Cp190 is required to form nonpromoter boundaries in fly embryos

**Fig. 1.**
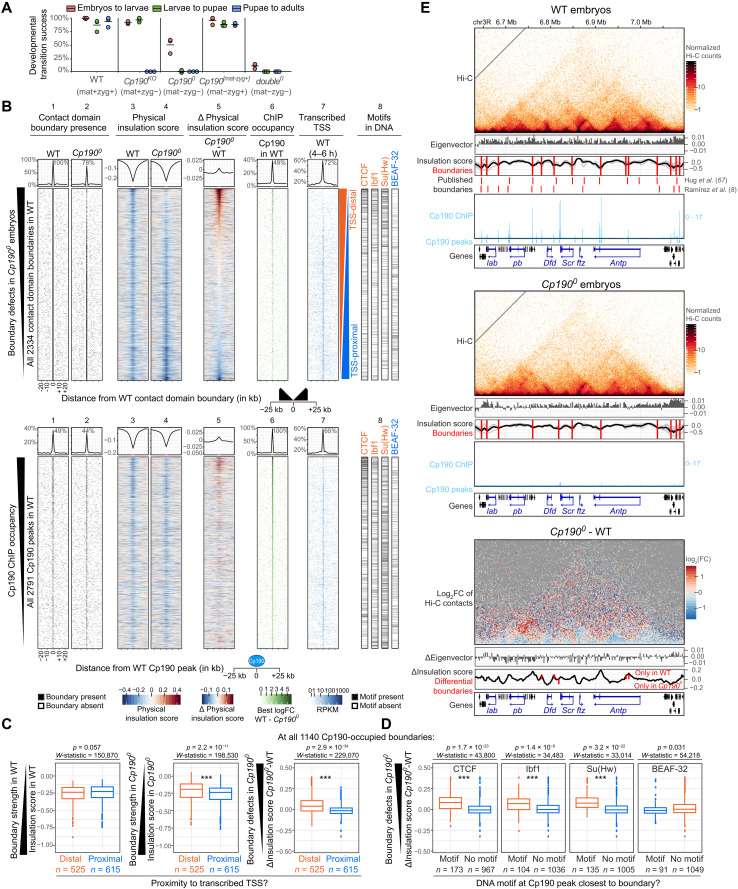
Cp190 is required to form nonpromoter boundaries in fly embryos. (**A**) Percentages of indicated genotypes (with/without maternal/zygotic protein) that completed indicated developmental transitions in three biological replicates. Horizontal lines show means. (**B**) Top: Distribution of indicated datasets in 2-kb bins ±25 kb around all WT contact domain boundaries ranked by physical insulation defects in *Cp190^0^*. Lane 8 shows the presence of indicated DNA motifs in the central 2-kb bin. Summarized values (average physical insulation score or percentage of WT boundaries with boundary/ChIP peak/transcribed TSS present) across 2-kb bins are shown. Enrichments ±2 kb around the central boundary are indicated. Bottom: Same but for all WT Cp190 ChIP peaks ranked by ChIP occupancy. (**C**) Physical insulation scores or differences measured at all 1140 Cp190-occupied boundaries whose most boundary-proximal Cp190 peak is distal or proximal to a transcribed TSS in WT embryos (*75*). *P* values and *W*-statistics from two-sided Wilcoxon rank sum test with continuity correction are indicated. (**D**) Similar to (C) but at all 1140 Cp190-occupied boundaries whose most boundary-proximal Cp190 peak overlaps or does not overlap the indicated DNA motif. (**E**) Example locus (dm6 coordinates) Hi-C maps (2-kb resolution), eigenvector values (2-kb resolution, positive/negative for A/B compartments), physical insulation score (calculated with different window sizes in gray, average in black), and contact domain boundaries (red lines) from this (above) and published (below) Hi-C studies (*8*, *67*) and Cp190 ChIP-seq (reads per million). Homeobox genes are blue. Differential (*Cp190^0^* minus WT) values are shown below.

To address whether Cp190 is required to form contact domain boundaries, we performed Hi-C on 2- to 6-hour-old wild-type (WT) and *Cp190^0^* embryos lacking both maternal and zygotic *Cp190* gene products (fig. S1A). This early developmental stage was chosen to avoid confounding effects of lethality of *Cp190^0^* mutants, as half of *Cp190^0^* mutants arrest development at late embryogenesis with the remaining animals dying as young larvae (Fig. 1A). Two four-cutter restriction enzymes were combined for enhanced resolution, and Hi-C maps consisting of 80 million reads per genotype were obtained by combining the four biological replicates (table S1). In parallel, Cp190 binding sites were mapped in embryos by anti-Cp190 ChIP-seq performed in biological triplicates. A total of 2791 Cp190 peaks were defined as enriched in WT relative to *Cp190^0^* mutants (data S1). To assess the relation between Cp190 peaks and contact domain boundaries in 2- to 6-hour-old embryo Hi-C maps, boundaries were identified at 2-kb resolution with TopDom (data S2) (*41*). Boundaries within ±2 kb of a Cp190 ChIP peak were defined as Cp190-occupied boundaries. Physical insulation scores were also measured for every 2-kb bin in the genome to determine how strongly Hi-C contacts are depleted across a region of interest such as a boundary or a ChIP peak (small physical insulation scores indicate strong physical insulation) (data S3). The difference of physical insulation scores in mutant versus WT embryos was calculated to assess boundary defects in mutants.

In Fig. 1B (top), all 2334 contact domain boundaries called in WT embryos are ranked by strongest (top) to weakest (bottom) boundary defects in *Cp190^0^* embryos. WT and *Cp190^0^* Hi-C maps were globally similar, and compartmental interactions were unaffected (fig. S1, B and C, and data S4). However, 22% of all contact domain boundaries detected in WT were lost in *Cp190^0^* mutants (Fig. 1B, lanes 1 and 2). Additional boundaries were retained but weaker in *Cp190^0^* than in WT, and overall, 26% of all WT boundaries were either lost or strongly weakened in *Cp190^0^* (Fig. 1B, lanes 1 to 5). The remaining three-quarters of boundaries were unaffected by Cp190 loss (Fig. 1B, lanes 1 to 5). Many of these unaffected boundaries were proximal [within ±200 base pairs (bp)] to an active transcription start site (TSS) [Fig. 1B (lane 7) and fig. S1D]. Consistently, boundary defects were significantly higher at promoter-distal boundaries than at promoter-proximal boundaries in *Cp190^0^* mutants, although Cp190-occupied promoter-distal and promoter-proximal boundaries had similar strengths in WT embryos (Fig. 1C and fig. S1, E and F).

A previous analysis of boundaries defined in a high-resolution Hi-C study revealed that promoter and nonpromoter boundaries are differentially enriched in DNA motifs and differentially bound by the corresponding transcription factors (*8*). Consistently, we found that the most common motifs enriched at nonpromoter boundaries [CTCF, Ibf1 (insulator binding factor 1), and Su(Hw)] were visibly enriched at Cp190-dependent boundaries, while motifs enriched at promoter boundaries [such as BEAF-32 (boundary element-associated factor of 32 kD), M1BP (motif 1 binding protein), core motif-6, and ZIPIC (zinc-finger protein interacting with CP190)] were visibly enriched at Cp190-independent boundaries [Fig. 1B (lane 8) and fig. S1D]. Conversely, boundary defects measured in *Cp190^0^* mutants at boundaries occupied by Cp190 in WT were significantly higher when Cp190 peaks overlapped CTCF, Ibf1, or Su(Hw) motifs (Fig. 1D and fig. S1G). In contrast, boundary defects in *Cp190^0^* were not higher at boundaries occupied by Cp190 in WT that overlapped BEAF-32, ZIPIC, M1BP, or core motif-6 than at boundaries not overlapping these motifs (Fig. 1D and fig. S1G).

To determine how generally physical insulation defects are observed at former Cp190 peaks in *Cp190^0^* irrespective of their localization at contact domain boundaries defined by TopDom, physical insulation score differences between WT and *Cp190^0^* mutants were measured across all 2791 Cp190 peaks [ranked by ChIP occupancy in Fig. 1B (bottom)]. Domain boundaries were enriched within ±2 kb of many (49%) Cp190 peaks (Fig. 1C, lane 1). Boundary defects in *Cp190^0^* mutants were only observed at a subset of former Cp190 peaks, with more prominent defects visible at Cp190 peaks that are highly occupied in WT [Fig. 1C (lane 5) and fig. S1H].

These results are illustrated at the *Antennapedia* complex (ANT-C) comprising five HOX genes that determine the identity of anterior body segments (Fig. 1E). Cp190 was bound at most contact domain boundaries in WT embryos but was undetectable in *Cp190^0^* (Fig. 1E). Some boundaries were lost in *Cp190^0^*, and others were weaker but retained (Fig. 1E). The consequences of these blurred contact domain boundaries on HOX gene regulation are described later in the “Cp190 prevents regulatory cross-talk between early patterning gene loci” section.

We conclude that Cp190 is required to form one-quarter of all fly domain boundaries and is thus the major architectural protein required for fly domain boundary formation described to date. Although Cp190 is widely associated with domain boundaries, it only mediates formation of nonpromoter boundaries. Some boundaries are weakened but persist in *Cp190^0^* mutants, suggesting that Cp190 synergizes with other boundary-forming mechanisms at these sites.

### Cp190 is required for boundary formation at CTCF peaks

**Fig. 2.**
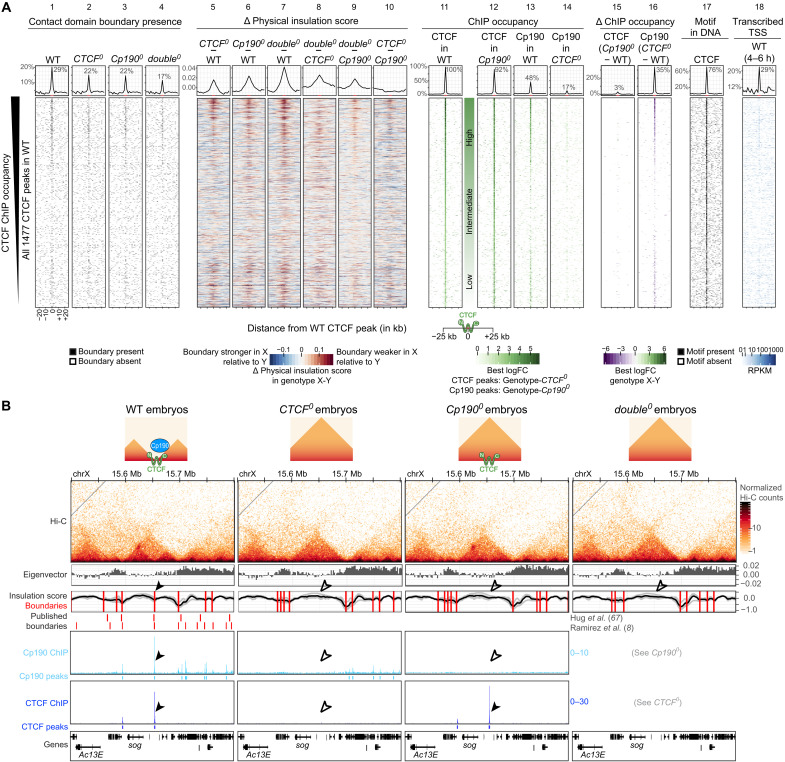
Cp190 is required for boundary formation at CTCF peaks. (**A**) Distribution of indicated datasets in ±25-kb windows centered around all 1477 CTCF peaks identified in WT ranked by ChIP occupancy. (Lanes 1 to 4) Presence of contact domain boundaries called in each genotype by TopDom in 2-kb bins around the peak center. (Lanes 5 to 10) Physical insulation score differences measured in genotype X (top) minus genotype Y (bottom) by Hi-C. (Lanes 11 and 12) CTCF or (Lanes 13 and 14) Cp190 ChIP occupancy in indicated genotypes. (Lanes 15 and 16) Differential CTCF and Cp190 ChIP occupancy in genotype X (top) minus genotype Y (bottom). (Lane 17) CTCF motif presence in DNA. (Lane 18) Expressed TSSs in WT 4- to 6-hour-old embryos (*75*). Summarized values (average physical insulation score or percentage of WT CTCF peaks with boundary/ChIP peak/differentially bound region/CTCF motif/transcribed TSS present) across 2-kb bins are shown above with indicated enrichments ±2 kb around the central CTCF peak (highlighted in red on the *x* axis). (**B**) Example locus (dm6 coordinates) Hi-C maps presented as in Fig. 1E. Arrowheads point to a CTCF and Cp190 cobound peak located at a domain boundary in WT; empty arrowheads indicate their absence in the mutants. ChIP-seq scale is reads per million. Cartoons summarize CTCF and Cp190 binding status and boundary presence/absence. RPKM, reads per kilobase per million reads.

Our finding that a subset of Cp190-dependent boundaries is enriched for CTCF motifs (Fig. 1B, lane 8) and the fact that CTCF recruits Cp190 to CTCF binding sites (*7*, *26*, *27*) led us to hypothesize that Cp190 is an essential cofactor required for the ability of CTCF to form robust boundaries. This hypothesis predicts that only those CTCF peaks that are cobound by Cp190 would be physical boundaries. To test this, we identified 1477 CTCF peaks defined as enriched in WT relative to *CTCF^0^* mutants by anti-CTCF ChIP-seq (data S5) and assessed the location of boundaries around CTCF ChIP peaks. Thirteen percent of all contact domain boundaries in WT embryos were located within ±2 kb of a CTCF peak (fig. S2A, lane 11), consistent with previous reports that CTCF is only enriched at a subset of boundaries (*7*, *8*). When all CTCF peaks were ranked from highest (top) to lowest (bottom) ChIP occupancy (Fig. 2A, lane 11), CTCF peaks with high or low CTCF occupancy were associated with contact domain boundaries but an abundant class of CTCF peaks with intermediate ChIP occupancy were clearly not [Fig. 2A (lane 1) and fig. S2B]. CTCF peaks without associated boundaries did not colocalize with Cp190, whereas higher and lower occupancy CTCF peaks did (Fig. 2A, lane 13). CTCF+Cp190 colocalization was significantly positively associated with localization at a boundary (fig. S2C). By assessing their genomic locations, we realized that CTCF peaks not colocalizing with Cp190 correspond to previously described CTCF standalone peaks that are frequently located in introns in contrast to CTCF+Cp190 cobound peaks (fig. S2D) (*30*, *42*). We had not noticed that CTCF standalone peaks are not physical boundaries in our previous analysis of *CTCF^0^* mutant larval central nervous systems (*7*) because many CTCF peaks with intermediate occupancy in WT embryos are low occupancy peaks in WT larval nervous systems (fig. S2, E and F). We had thus previously assumed that domain boundaries were difficult to detect at weakly occupied CTCF peaks. These new results in *CTCF^0^* embryos, however, clearly reveal that physical boundaries are only present at CTCF sites that are co-occupied by Cp190. Cp190 might therefore be required for boundary formation at CTCF peaks, or alternatively, boundaries may not be established when CTCF binds at specific genomic locations (such as introns).

If Cp190 is a CTCF cofactor required for robust boundary formation, a second prediction is that CTCF-dependent boundaries should also depend on Cp190. To test this, we performed Hi-C on *CTCF^0^* and *double^0^* (completely lacking both CTCF and Cp190; fig. S1A) 2- to 6-hour-old embryos, in parallel to the WT and *Cp190^0^* embryos described above (table S1). Cp190 ChIP peaks were also mapped in *CTCF^0^*, and CTCF ChIP peaks were mapped in *Cp190^0^*. Fewer *double^0^* mutants completed embryogenesis compared to *Cp190^0^*, indicating that additional loss of CTCF aggravated the embryonic lethality of *Cp190^0^* (Fig. 1A). In *CTCF^0^* mutants, physical insulation defects were observed at many former CTCF+Cp190 cobound peaks [Fig. 2A (lanes 2 and 5) and data S2 and S3]. Cp190 binding was reduced at most of these sites [Fig. 2A (lanes 13, 14, and 16) and data S6 and S7], and we therefore could not say whether CTCF acts alone or together with Cp190 to form these boundaries. In *Cp190^0^* mutants, however, CTCF binding was largely unaffected [Fig. 2A (lanes 11, 12, and 15), and data S8 and S9], yet boundaries were defective at formerly Cp190 cobound CTCF peaks relative to WT (Fig. 2A, lane 6). An example of a domain boundary located at a CTCF+Cp190 cobound peak that relies on both CTCF and Cp190 is shown in Fig. 2B. This demonstrates that Cp190 is required for DNA-bound CTCF to form a robust boundary.

The fact that only a subset of CTCF peaks colocalize with Cp190 suggests that CTCF also exerts Cp190-independent functions. To test this, we introduced transgenes expressing truncated *CTCF* versions completely lacking CTCF N (*CTCF*^Δ*N*^) or C termini (*CTCF*^Δ*C*^) into *CTCF^0^* animals. *CTCF*^Δ*C*^ mutants lack the Cp190-interacting domain, and *CTCF*^Δ*N*^ lack the cohesin-interacting domain (*7*). Both truncated CTCF proteins were expressed in vivo (fig. S2G) and enabled about one-third of *CTCF^0^* mutants, which never hatch from the pupal case, to hatch into very short-lived adults (fig. S2H). The fact that CTCF lacking its C terminus retains some limited function indeed suggests that CTCF exerts some Cp190-independent functions.

### Boundary defects in *CTCF^0^* correlate with Cp190 retention

**Fig. 3.**
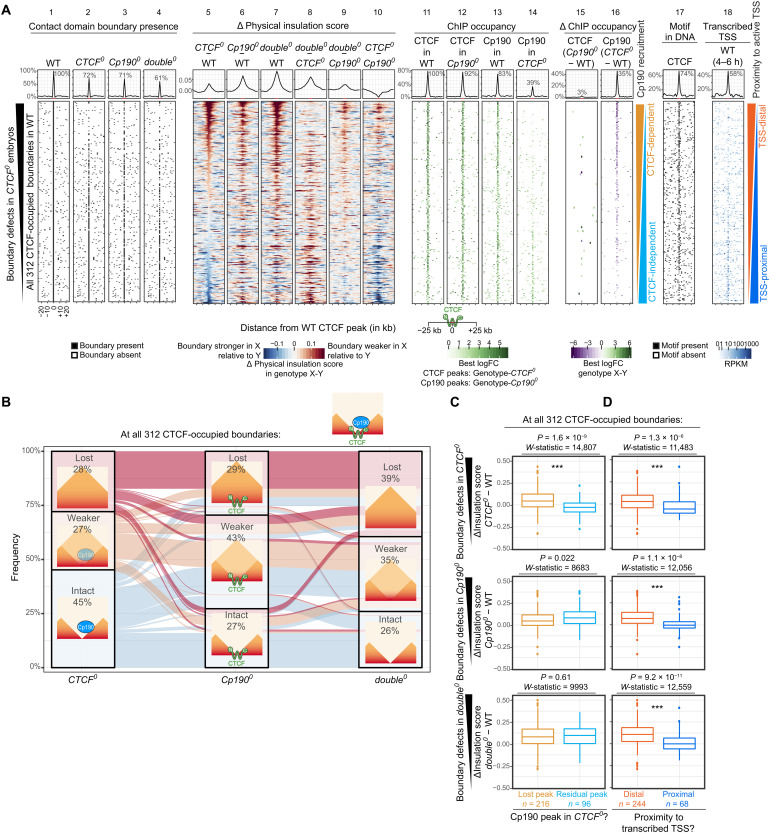
CTCF-occupied boundaries are differentially sensitive to CTCF or Cp190 loss. (**A**) Same as Fig. 2A but for all 312 CTCF-occupied boundaries ranked by physical insulation defects in *CTCF^0^* relative to WT. (**B**) Alluvial plot of the same data as in (A). Lost boundaries are absent in the mutant but present in WT. Weaker and intact boundaries are retained in the mutant but have a delta physical insulation score in the mutant minus WT ≥0.01 or <0.01, respectively. Cartoons show typical CTCF and Cp190 ChIP occupancy in each scenario. (**C**) Box plots of data shown in (A). Physical insulation score differences in indicated mutants minus WT at all 312 CTCF-occupied boundaries at which Cp190 recruitment to the nearest CTCF peak to the boundary is strictly CTCF dependent (lost Cp190 peak in *CTCF^0^*) or at least partially CTCF independent (residual Cp190 peak at the former CTCF peak in *CTCF^0^*). *P* values and *W*-statistics from two-sided Wilcoxon rank sum test with continuity correction are indicated. Box plot: Center line, median; box limits, upper and lower quartiles; whiskers, 1.5× interquartile ranges; points, outliers; *n* = CTCF-occupied boundaries of indicated categories. (**D**) Same as (C) but for CTCF-occupied boundaries at which the nearest CTCF peak to the boundary is distal (further than ±200 bp) or proximal (within 200 bp) to an actively transcribed TSS (RPKM > 0) in WT embryos (*75*). (C and D) *** indicate high statistical significance (*P* ≤ 0.0001). The precise *P* values are shown in the figures.

If Cp190 is required for robust boundary formation, then a third prediction is that boundaries co-occupied by CTCF+Cp190 will be retained in *CTCF^0^* mutants if Cp190 is retained at the boundary despite CTCF loss. To test this, we focused on CTCF-occupied boundaries, defined as those within ±2 kb of a CTCF peak in WT (Fig. 3A, lanes 1 and 11). All 312 CTCF-occupied boundaries were ranked from strongest (top) to weakest (bottom) physical insulation defects in *CTCF^0^* relative to WT (Fig. 3A, lane 5). Only 28% of these boundaries were lost in *CTCF^0^* [Figs. 3, A (lane 2) and B]. Several boundaries were even unexpectedly reinforced in *CTCF^0^* relative to WT (Fig. 3A, lane 5). Boundaries that remained intact in *CTCF^0^* retained a residual Cp190 peak (Fig. 3A, lanes 5 and 14), revealing that Cp190 is recruited there at least partially CTCF independently. Boundaries at which Cp190 was retained in *CTCF^0^* mutants were often promoter-proximal (Fig. 3A, lanes 14 and 18), suggesting that Cp190 may be redundantly recruited by promoter-associated factors. Conversely, boundaries that retained a residual Cp190 peak were significantly less weakened in *CTCF^0^* than those that lost Cp190 [Fig. 3C and fig. S3A (top)]. This effect was not seen in *Cp190^0^* [Fig. 3C and fig. S3A (middle)] or *double^0^* [Fig. 3C and fig. S3A (bottom)], indicating that boundary retention in *CTCF^0^* correlates with Cp190 presence.

More than half of CTCF-occupied boundaries that were intact in *CTCF^0^* were lost or weakened in *Cp190^0^* (Fig. 3B). In addition, the average physical insulation score defect measured at CTCF-occupied boundaries was larger in *Cp190^0^* than in *CTCF^0^* (Fig. 3A, top of lanes 5 and 6). Cp190 is therefore also required to form boundaries occupied by CTCF but unaffected or, in some cases, unexpectedly reinforced in *CTCF^0^*. Nevertheless, some boundaries were more strongly affected in *CTCF^0^* than in *Cp190^0^* (Fig. 3A, top of lane 10), indicating that CTCF retains some ability to form boundaries without Cp190 at several sites.

Seventy-two to 74% of CTCF-occupied boundaries were lost or at least measurably weaker in *Cp190^0^* or *double^0^* mutants (Fig. 3B), indicating that Cp190 is required to form robust boundaries at many, but not all, CTCF-occupied boundaries. Boundary defects in all genotypes were significantly weaker at CTCF-occupied boundaries that were TSS-proximal than those that were TSS-distal [Fig. 3, A (lanes 5 to 7 and 18) and D, and fig. S3B], again suggesting that transcription-dependent mechanisms may redundantly form boundaries, as we observed for all Cp190-occupied boundaries (Fig. 1C).

An example locus illustrating these results is shown in fig. S3C. We conclude that Cp190 reinforces several CTCF-occupied boundaries independently of CTCF. CTCF-occupied boundaries to which Cp190 is recruited by additional factors other than CTCF are more sensitive to Cp190 loss because CTCF loss is compensated by redundant Cp190 recruitment.

### Su(Hw) recruits Cp190 to a distinct subset of Cp190-dependent boundaries

Of boundaries occupied by Cp190 in WT and lost in *Cp190^0^*, only 40% are co-occupied by CTCF. Su(Hw) occupies a distinct subset of nonpromoter boundaries than CTCF (*8*) and directly recruits Cp190 to some of its binding sites (*30*). Some Cp190-dependent boundaries are enriched for Su(Hw) motifs (Fig. 1B), suggesting that Su(Hw) recruits Cp190 to these sites to form boundaries. To test this, we identified Su(Hw)-dependent Cp190 peaks. Cp190 ChIP-seq could not directly be performed on *su(Hw)^0^* mutant embryos lacking maternal and zygotic Su(Hw) because Su(Hw) is essential for female germline development (*43*). Instead, we performed Cp190 ChIP-seq in larval central nervous systems of *su(Hw)^KO^* mutants with a deletion of the *su(Hw)* open reading frame and diluted maternal Su(Hw) and in WT and *Cp190^KO^* mutants as controls (fig. S4A and data S10 to S12) and subsequently intersected Cp190 peaks identified in larval central nervous systems with Cp190-occupied boundaries in WT embryos. Of 1140 Cp190-occupied boundaries in WT embryos, 1125 (99%) overlapped a Cp190 peak in WT larval central nervous systems. Among these, 88 of 1125 (8%) did not overlap a Cp190 peak in *su(Hw)^KO^* larval central nervous systems. Physical insulation defects in *Cp190^0^* embryos were significantly larger at boundaries overlapping Su(Hw)-dependent Cp190 peaks than at boundaries overlapping Su(Hw)-independent Cp190 peaks (fig. S4B). Together, these results suggest that Cp190 is recruited to independent sites by CTCF and Su(Hw) to form boundaries (fig. S4C).

### Diverse Cp190 complexes exert similar enhancer-blocking activity in a reporter assay

**Fig. 4.**
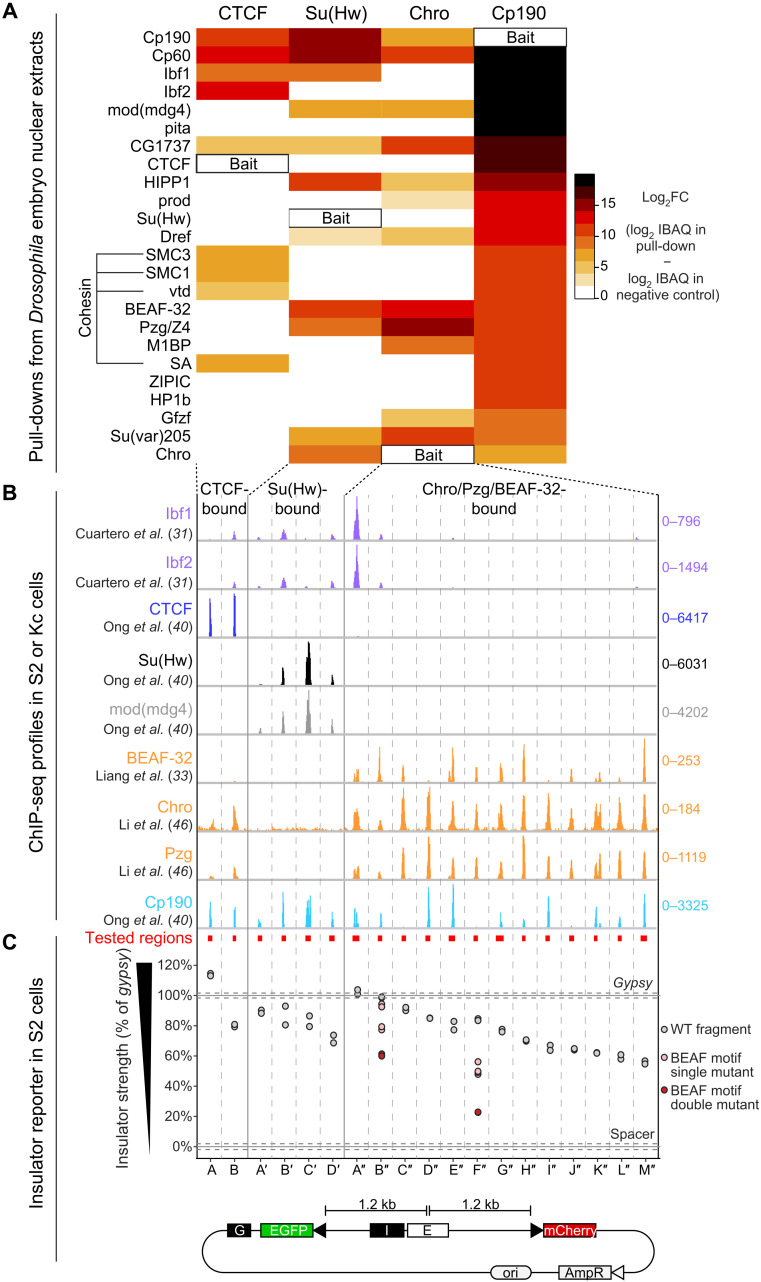
Cp190 complexes exert similar enhancer-blocking activity in a quantitative reporter assay. (**A**) Enrichments of indicated proteins (rows) in pull-downs with indicated GFP-tagged baits (columns, Su(Hw)[1-219], CTCF[1-293], Chro[613-926], and full-length Cp190) from the same batches of embryo nuclear extracts, analyzed by mass spectrometry (MS) and ranked by their specific enrichment in the Cp190 pull-down. Scale bar indicates log_2_ fold change (FC) of average intensity based absolute quantification (iBAQ) values of biological duplicate pull-downs over negative control pull-downs with unrelated GFP-tagged bait. CTCF pull-downs were previously described (*7*). (**B**) Published ChIP-seq profiles in S2 or Kc cell lines of indicated insulator proteins ±1 kb around the cloned genomic fragments (345 to 888 bp long indicated by red boxes, loci separated by vertical lines). Scales show total counts. (**C**) Insulator strengths of cloned genomic fragments measured in S2 cells transiently transfected with reporters with indicated I (insulator) test fragments, expressed as percentage of *gypsy* insulator strength (set to 100%). Insulators block EGFP activation by the enhancer (E). A *gypsy* insulator (“G”) blocks EGFP activation by the enhancer from the left. Fragments were tested in biological duplicates (dots). Horizontal lines show average values obtained with *gypsy* or a neutral spacer (*n* = 8 biological replicates); dotted lines show SDs. Tested fragments were bound by CTCF (A and B), Su(Hw) (A′ to D′), or Chro+Pzg+BEAF-32 (A″ to M″). Single (pink dots) or two (red dots) pairs of non-overlapping BEAF motifs in B″ and F″ were mutated.

Our finding that Cp190 associates with both promoter and nonpromoter boundaries but is only required to form the latter (Fig. 1B) raises the question of whether Cp190 exerts different activities at different boundaries, possibly in the context of distinct multiprotein complexes. To test this, we first clarified the compositions of distinct Cp190-containing complexes since these complexes were previously purified from different sources using different protocols, precluding their direct comparison. We pulled down CTCF (*7*), Su(Hw), Chro, and Cp190 from the same batches of *Drosophila* embryonic nuclear extracts (data S13). Cp190 copurified with all expected insulator-binding proteins such as Ibf1, Ibf2, mod(mdg4), pita, CTCF, Su(Hw), BEAF-32, and ZIPIC (Fig. 4A) (*25*, *27*, *31*, *32*). Cp190, CTCF, Su(Hw), and Chro pull-downs identified partially overlapping sets of copurifying proteins, and all contained Cp190, Cp60 [Cp190’s partner protein at centrosomes (*44*)], and CG1737 [which previously copurified with HP1a (heterochromatin protein 1) (*45*)] (Fig. 4A). Recombinant Cp190-Cp60 complexes directly interacted with CTCF C terminus, Su(Hw) N or C terminus, or full-length Ibf1 or 2, and CTCF directly interacted with Cp60 in addition to its previously known direct interaction with Cp190 (fig. S4, D and E) (*7*). Cohesin subunits (SMC1, SMC3, SA, and vtd) were specifically enriched in CTCF and Cp190 pull-downs (Fig. 4A). Proteins copurifying with CTCF, Su(Hw), or Chro generally colocalized with these proteins in published ChIP-seq experiments (Fig. 4B) (*31*, *33*, *40*, *46*).

We then tested whether different Cp190 complexes assembled at separate loci exert enhancer-blocking activity in a quantitative insulator reporter assay (*7*). Test fragments (345 to 888 bp long, average 496 bp) were cloned in between an enhanced green fluorescent protein (EGFP) reporter and an enhancer, while an mCherry reporter present at a similar distance from the enhancer serves as a reference. Relative EGFP and mCherry intensities were measured in thousands of single transfected *Drosophila* S2 (Schneider’s *Drosophila* Line 2) cells with a cell analyzer. Sites bound by CTCF, Su(Hw), or Chro+Pzg+BEAF-32 specifically reduced EGFP fluorescence to varying degrees relative to the well-characterized *gypsy* insulator (Fig. 4C) (*7*, *47*). We mutagenized two boundaries each containing two pairs of overlapping BEAF-32 motifs (*48*). Single point mutations in both BEAF-32 motif pairs had a stronger effect than mutating a single pair, indicating that each pair of overlapping BEAF-32 motifs contributes independently to insulator activity (Fig. 4C). Prior transgenic insulator reporter assays in flies concluded that only a subset of insulator protein–bound sites are insulators or that sites must be multimerized to reveal insulator function (*30*, *49*–*51*). Insulator activity depends on chromatin context (*30*), and the robustness of our transiently transfectable reporter suggests that it is chromatin context independent. We conclude that Cp190 assembles into diverse multisubunit protein complexes bound at distinct genomic loci that exert similar enhancer-blocking activities in a reporter assay.

### Cp190 prevents regulatory cross-talk between early patterning gene loci

**Fig. 5.**
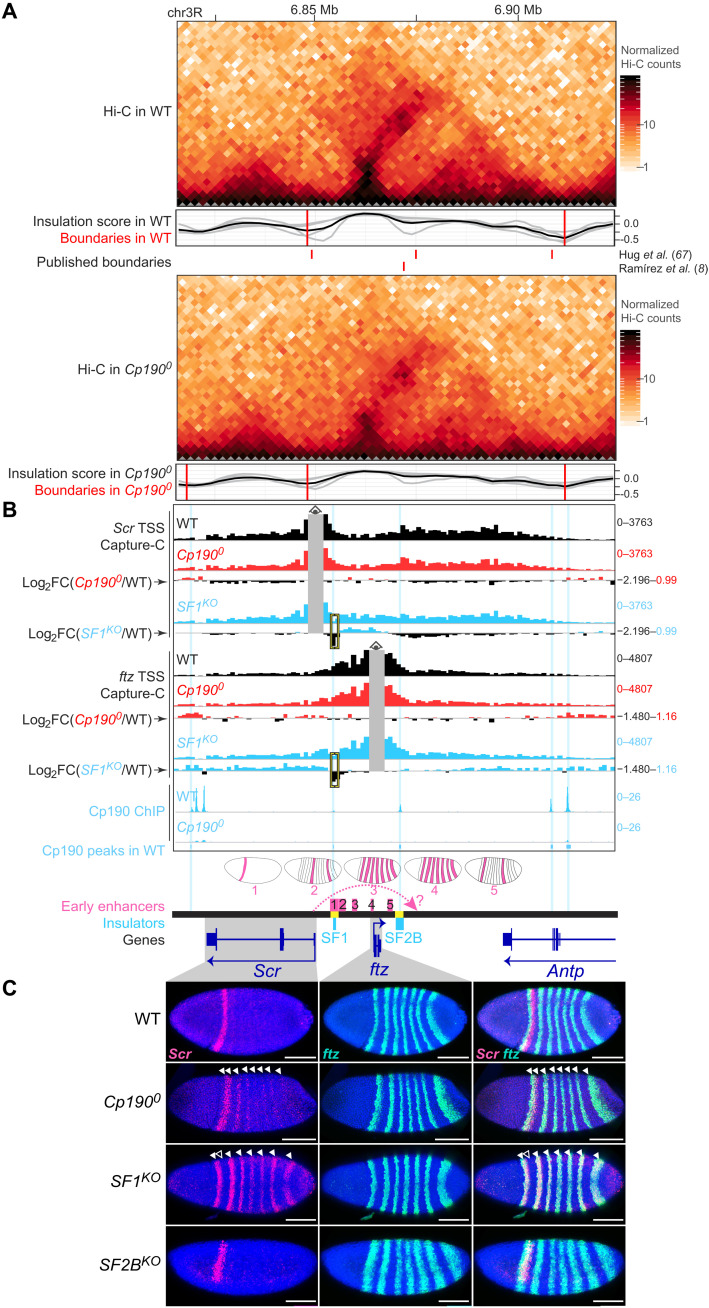
Cp190 prevents regulatory cross-talk between early patterning gene loci. (**A**) Extended *ftz* locus (dm6 coordinates) Hi-C maps presented as in Fig. 1E. (**B**) NG Capture-C profiles (1-kb resolution) around *Scr* or *ftz* TSS viewpoints showing average normalized reads (in reads per million) of biological triplicates excluding bins ±2 kb around the viewpoint (gray). Differential Capture-C profiles in mutant versus WT are shown as log_2_ fold change profiles obtained from diffHic. Yellow brackets mark the deleted boundary in *SF1^KO^*. WT Cp190 ChIP peaks are highlighted in blue (two prominent Cp190 ChIP signals in WT overlap a blacklisted region and were not called peaks). Early stripe enhancers with schematized expression patterns are numbered 1 to 5 (references in Materials and Methods). Dotted arrow shows *Scr* regulation by a hypothetical distal enhancer (question mark). *SF1^KO^* and *SF2B^KO^* deletions are yellow. (**C**) RNA–fluorescence in situ hybridization (FISH) with cohybridized antisense probes against *Scr* (red) and *ftz* (green) mRNAs in 4′,6-diamidino-2-phenylindole (DAPI)–stained early gastrula embryos (anterior left; posterior right; scale bars, 100 μm; merged images on the right). In *Cp190^0^*, *Scr* is expressed in its WT stripe and in ectopic *ftz* stripes (filled arrowheads). In *SF1^KO^* embryos, *Scr* is lost in its WT stripe (empty arrowhead) and only expressed in *ftz* stripes (filled arrowheads). In *SF2B^KO^* embryos, *Scr* and *ftz* expression seem normal (embryo rotation reveals a normal ventral gap in *Scr* expression).

Given its critical boundary function, we then investigated how Cp190 affects the expression of well-studied developmental genes. We first focused on the ANT-C locus comprising essential developmental genes and harboring several contact domain boundaries that were defective in *Cp190^0^* (Figs. 1E and 5, A and B, and fig. S5A). More specifically, we focused on the extended *Sex combs reduced* (*Scr*) locus because the spatial and temporal activity patterns of enhancers present in a 70-kb region around *Scr* have been systematically characterized in WT embryos (*52*, *53*), enabling us to interpret gene misexpression phenotypes in *Cp190^0^* mutants with respect to local enhancers. *Scr* is a HOX gene conferring segmental identity to specific anterior body segments, and its neighboring gene *fushi tarazu* (*ftz*) is a pair-rule homeodomain gene required to help segment the very early embryo (*54*). *Scr* and *ftz* are expressed in independent spatiotemporal patterns in early embryos. *ftz* is expressed in seven equally spaced stripes beginning after zygotic genome activation, while *Scr* is expressed later in early gastrulae in a band of cells that partially overlaps the first *ftz* stripe (Fig. 5C). *ftz* expression is driven by stripe enhancers contained within the *ftz* contact domain (numbered 2 to 5 in Fig. 5B) (*52*, *55*, *56*). This domain interrupts a larger contact domain containing *Scr* and its enhancers, including a putative distal *Scr* enhancer located downstream of *ftz* (question mark in Fig. 5B) (*52*). Two characterized insulators named *Scr-ftz* (SF) boundaries SF1 and SF2B (*57*, *58*) overlap *ftz* contact domain boundaries (Fig. 5B). According to a published model, SF1 and SF2B pair and thereby “loop” *ftz* out of the *Scr* domain to prevent *Scr*-*ftz* regulatory cross-talk and enable the *Scr* promoter to skip the intervening *ftz* domain and reach its putative distal enhancer (shown as a dotted arrow in Fig. 5B) (*58*, *59*). To clarify how SF boundaries function and understand how Cp190 contributes to boundary function, we analyzed boundary defects at higher resolution and examined *Scr* and *ftz* expression in *Cp190^0^* mutants and in embryos carrying deletions of SF1 (*SF1^KO^*) or SF2B (*SF2B^KO^*).

Cp190 binds to *ftz* contact domain boundaries in WT, and *ftz* boundaries were slightly weakened in *Cp190^0^* Hi-C maps (Fig. 5A and fig. S5A). Interdomain contacts between *Scr* and *ftz* contact domains were not significantly increased in higher-resolution next-generation (NG) Capture-C (*60*) experiments on 2- to 6-hour-old WT and *Cp190^0^* embryos with viewpoints in *Scr* and *ftz* TSSs (Fig. 5B and data S14). In *Cp190^0^* mutants, *Scr* was expressed in its endogenous stripe and in seven stripes overlapping *ftz* expression (Fig. 5C). This suggests that *ftz* enhancers are able to ectopically activate *Scr* transcription upon Cp190 loss despite retention of a physical boundary.

Deletion of SF1 boundary DNA led to stronger interdomain contacts between *Scr* TSS and *ftz* contact domains than Cp190 loss, as revealed by simultaneous NG Capture-C on 2- to 6-hour-old *SF1^KO^* embryos (Fig. 5B and data S15). In contrast to *Cp190^0^*, *SF1^KO^* embryos lost *Scr* expression in its endogenous anterior stripe, and *Scr* expression was completely replaced by the *ftz* pattern (Fig. 5C) as recently described (*59*). This result had previously been interpreted to support the model in which SF1-SF2B pairing is required to bridge *Scr* to its putative distal enhancer downstream of *ftz* (*58*, *59*). Inconsistent with this model, however, *Scr* expression was normal in *SF2B^KO^* mutant embryos (Fig. 5C). Instead, we found that *SF1^KO^* embryos likely lose endogenous *Scr* expression because SF1 deletion concomitantly deletes an enhancer that we noticed was active in an early *Scr*-like stripe (labeled 1 in Fig. 5B and fig. S5B) (*53*). We conclude that both Cp190 protein and SF1 DNA critically form a regulatory boundary, ensuring independent regulation of *Scr* and *ftz*, but neither Cp190 nor SF1-SF2B pairing is required for endogenous *Scr* expression. Stronger *Scr* misexpression in *ftz* stripes observed in *SF1^KO^* than in *Cp190^0^* (Fig. 5C) correlates with stronger *Scr* TSS-*ftz* interdomain contacts observed upon SF1 deletion than upon Cp190 loss (Fig. 5B). We note that we do not know how Cp190 is recruited to *ftz* boundaries, as they are not CTCF- or Su(Hw)-dependent Cp190 peaks and *CTCF^0^* mutants did not show any contact domain boundary or gene misexpression defects at this locus (figs. S5, A and C).

### Cp190 is dispensable for HOX gene activation by long-range enhancers

**Fig. 6.**
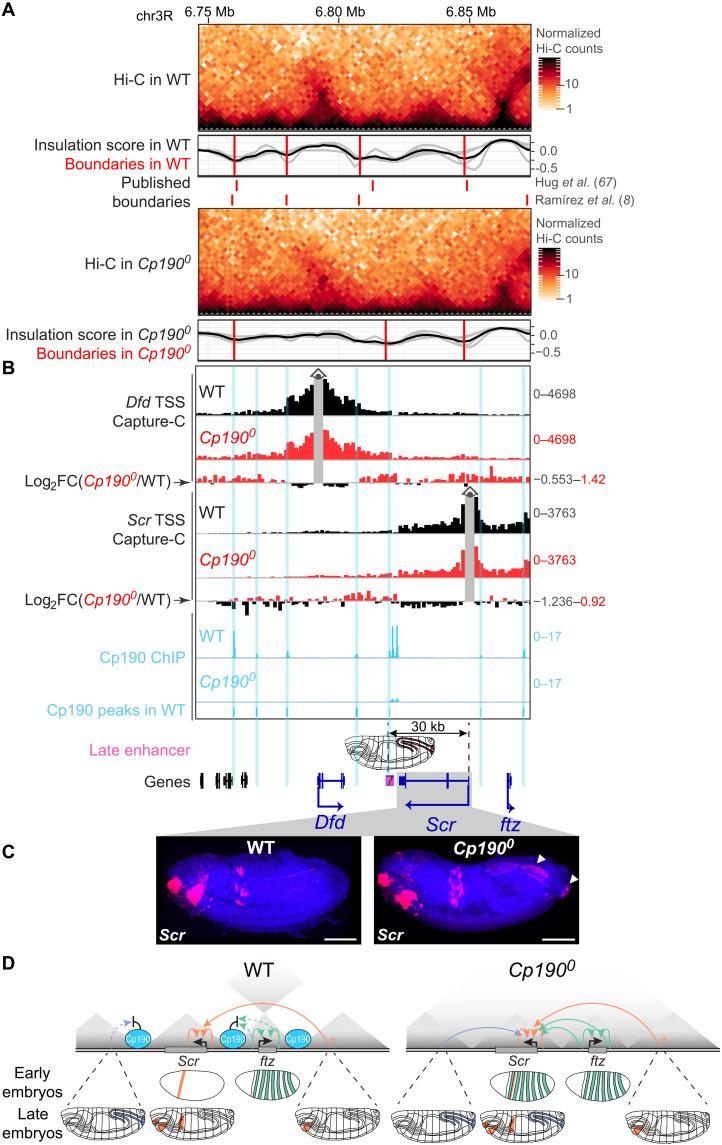
Cp190 is dispensable for ectopic *Scr* activation by a long-range enhancer. (**A**) Similar to Fig. 5A but showing contact domains downstream of *Scr*. (**B**) NG Capture-C profiles presented as in Fig. 5B but around *Dfd* or *Scr* TSS viewpoints in indicated genotypes. Enhancer 7 drives schematized reporter gene expression in the hindgut and anal plate of older embryos in transgene assays (*52*) and is separated from the *Scr* promoter (30 kb away) by Cp190 ChIP peaks. (**C**) RNA-FISH with antisense probes (red) against *Scr* mRNA in late-stage (stage 16) DAPI-stained embryos (anterior left; posterior right; scale bars, 100 μm). *Scr* is normally expressed in labial and prothoracic segments and the anterior midgut and is additionally expressed in the hindgut and anal plate (left and right arrowheads) of *Cp190^0^* mutants. (**D**) Summarized *Scr* misexpression phenotypes in *Cp190^0^* early and late embryos. Effective (solid arrows) or blocked (dotted arrows) transcriptional activation of promoters by indicated enhancers is shown (hindgut and anal plate enhancer in blue; *Scr* enhancers in orange including a putative distal enhancer marked by a question mark; *ftz* enhancers in green). In *Cp190^0^* embryos, *Scr* is activated by its endogenous enhancers and additionally by formerly insulated enhancers, resulting in cumulated expression patterns.

*ftz* stripe enhancers are only active in early embryos, and *Cp190^0^* and *SF1^KO^* older embryos no longer ectopically expressed *Scr* in stripes (fig. S5D). Instead, older *Cp190^0^* embryos misexpressed *Scr* in the hindgut and anal plate (Fig. 6, A to C). Near-complete characterization of embryonic enhancers in the extended *Scr* locus previously identified a single hindgut and anal plate enhancer 30 kb downstream of the *Scr* promoter that could activate transcription from the *Scr* promoter in a transgene (labeled 7 in Fig. 6B) (*52*, *53*). Cp190 normally binds to a contact domain boundary separating this enhancer from the *Scr* promoter, and both Hi-C (Fig. 6A) and NG Capture-C (Fig. 6B) revealed qualitatively weakly increased contacts in broad contiguous regions across former Cp190 peaks in *Cp190^0^* mutants. This strongly suggests that in *Cp190^0^* mutants, the *Scr* promoter is ectopically activated by a long-range enhancer from which it was formerly insulated. Cp190 is therefore required to insulate *Scr* from noncognate enhancers but not to bridge *Scr* to distal enhancers (summarized in Fig. 6D).

We also examined the expression of additional ANT-C HOX genes other than *Scr* in WT and *Cp190^0^* embryos (fig. S6A). Expression of *Antennapedia* (*Antp*) was normal in *Cp190^0^* embryos (fig. S6B). In contrast, *Deformed* (*Dfd*) was strongly ectopically expressed in the nervous system of *Cp190^0^* mutants in addition to being expressed in its endogenous pattern (fig. S6B). Several neuronal enhancers have been annotated both within and flanking the *Dfd* contact domain, and we are not able to hypothesize which of these may be the culprit enhancer driving *Dfd* misexpression in *Cp190^0^* mutants. Nevertheless, as in the case of *Scr*, ectopic *Dfd* expression is overlaid onto its endogenous expression pattern in *Cp190^0^* mutants. Abdominal HOX genes of the bithorax complex (BX-C) are expressed from a separate locus than ANT-C (fig. S7A). These genes are controlled by body segment–specific enhancers delimited by boundaries that maintain the independence of these enhancer domains (*61*, *62*). Expression of *Ultrabithorax* (*Ubx*), *abdominal-A* (*abd-A*), and *Abdominal-B* (*Abd-B*) was mostly normal in *Cp190^0^* mutants (fig. S7B). Therefore, Cp190 is not essential for abdominal HOX gene activation by their long-range enhancers (over more than 50 kb in the case of the *iab-5* enhancer domain driving *Abd-B* transcription in parasegment 10). Graded expression of *Ubx* and *abd-A* was, however, somewhat altered, suggesting that enhancer domains were inadequately insulated from each other (fig. S7B). These effects were subtle compared to the more severe phenotypes of BX-C boundary deletions (*63*, *64*), revealing that redundant mechanisms maintain the independence of BX-C regulatory domains. Together, we conclude that in all cases examined (*Scr*, *ftz*, *Antp*, *Dfd*, *Ubx*, *abd-A*, and *Abd-B*), developmental regulator genes were expressed in their endogenous patterns and, in some cases, in additional cells upon Cp190 loss.

### Cp190 is required for enhancer-blocking but not long-range pairing by the Homie insulator

If Cp190 is indeed required for enhancer-blocking but not distal enhancer–facilitating functions of insulators, these two functions should be differentially sensitive to Cp190 loss. We tested this hypothesis using the classical Homie insulator known to support one of the longest-range enhancer-promoter contacts described in flies (*36*, *37*). Homie overlaps a Cp190-occupied contact domain boundary downstream of *eve*, but Cp190 was not visibly required for formation of this boundary (fig. S8).

**Fig. 7.**
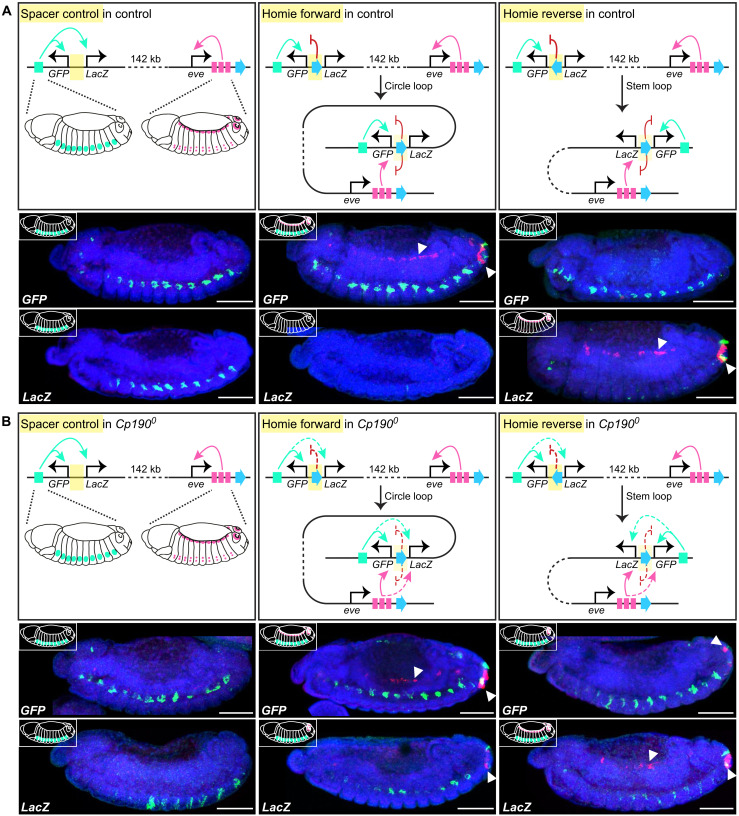
Cp190 supports enhancer-blocking but not long-range pairing by Homie. (**A**) WT expression of Fujioka *et al.* (*37*) transgenes with divergently transcribed *GFP* and *LacZ* reporter genes, integrated 142 kb upstream of *eve* in the vicinity of local *hebe* enhancers (green). *eve* endogenous enhancers (pink) are respectively active in anal plate, cardiac mesoderm, and specific neurons. When Homie insulator is between *GFP* and *LacZ*, it pairs in a head-to-head orientation with endogenous Homie downstream of *eve*, leading to schematized *GFP* and *LacZ* expression patterns. Below, RNA-FISH with antisense probes against *GFP* (top) or *LacZ* (bottom) mRNAs in midstage (stage 13) DAPI-stained control embryos with Cp190 (anterior left; posterior right; scale bars, 100 μm). RNA-FISH signal was false-colored green or pink when it was respectively detected in a deep (showing *hebe* enhancer-driven neuronal expression) or surface (showing cardiac mesoderm and anal plate expression marked by arrowheads) confocal slice. Note that neuronal expression driven by *hebe* enhancers masks that driven by *eve* enhancers, and anal plate signal is visible in all confocal slices. (**B**) Expression of same transgenes in *Cp190^0^*. Homie still supports long-distance reporter gene activation by distal *eve* enhancers (arrowheads) but is a weaker enhancer-blocker (as seen both by *LacZ* activation by *hebe* enhancer and activation of *LacZ/GFP* in the Homie forward/reverse transgenes, respectively, by the *eve* anal plate enhancer, although Homie is still able to block the *eve* cardiac enhancer).

Published Homie transgenes contain divergently transcribed reporter genes (*GFP* and *LacZ*) and are integrated 142 kb from *even-skipped* (*eve*), another pair-rule homeodomain gene similar to *ftz* (*37*). Local *hebe* gene enhancers close to the transgene integration site activate reporter gene expression in neurons of midstage WT embryos, except when Homie is present in between and specifically shields *LacZ* from *hebe* enhancers (Fig. 7A). In WT animals, transgenic Homie physically pairs with endogenous Homie in a head-to-head orientation and supports long-range reporter gene activation by *eve* enhancers active in anal plate, cardiac mesoderm, and specific neurons of mid-stage embryos (*36*, *37*) (Fig. 7A). When transgenic Homie is cloned in the same orientation as endogenous Homie (called forward orientation), it forms a circle loop enabling *GFP* activation by both local *hebe* and distal *eve* enhancers while preventing *LacZ* activation (*37*) (Fig. 7A). When transgenic Homie is cloned in the opposite reverse orientation, it forms a stem loop enabling *LacZ* activation by distal *eve* enhancers while ensuring that *GFP* is only activated by its nearby *hebe* enhancers (Fig. 7A) (*37*).

When the same Homie and control transgenes were introduced into *Cp190^0^* mutants, reporter genes were still activated by *eve* long-range enhancers with similar efficiencies as in WT (Fig. 7B), revealing that Cp190 is not essential for Homie pairing. *GFP* and *LacZ* reporters were, however, expressed in partially overlapping patterns in *Cp190^0^*, indicating that Homie’s enhancer-blocking activity partially relies on Cp190. Concretely, *LacZ* was activated (albeit more weakly than GFP) by *hebe* enhancers from which it was formerly insulated (Fig. 7B). In addition, Homie pairing enabled both *GFP* and *LacZ* activation by the *eve* anal plate enhancer in *Cp190^0^* mutants, although Homie still blocked activation of *LacZ* (in Homie forward) and *GFP* (in Homie reverse) by the *eve* cardiac mesoderm enhancer (Fig. 7B). The anal plate enhancer is known to activate Homie transgenes located at much larger distances from *eve* (up to 2 Mb away) than the cardiac mesoderm enhancer, potentially suggesting that the anal plate enhancer is stronger and thus requires fully functional Homie to be blocked (*37*).

These results demonstrate that enhancer-blocking and enhancer-pairing functions traditionally ascribed to the classical Homie insulator are separable and reveal that Cp190 is only required for efficient enhancer blocking. Notably, these results were obtained in embryos heterozygous for Homie transgene, arguing that transvection could not influence the result.

## DISCUSSION

Cp190 was hypothesized 17 years ago to organize the genome into chromosomal loops and thereby ensure gene regulation specificity (*25*). Here, we tested this model by analyzing *Drosophila* completely lacking Cp190, CTCF, and both factors. We reached the following conclusions: (i) Cp190 is critical for early development (Fig. 1A). (ii) Cp190 is required to form most promoter-distal boundaries but is dispensable to form promoter-proximal boundaries (Fig. 1). (iii) Cp190 is recruited to CTCF-dependent boundaries and is required for their formation (Fig. 2). (iv) While Cp190 is strictly recruited by CTCF to some of these boundaries, it reinforces other CTCF-occupied boundaries CTCF-independently (Fig. 3). (v) Cp190 assembles into diverse multisubunit complexes that share similar enhancer-blocking activity in a quantitative insulator reporter assay in transfected cells (Fig. 4). (vi) Cp190 critically insulates the HOX gene *Scr* from inappropriate enhancers located up to 30 kb away from the *Scr* promoter (Figs. 5 and 6). (vii) In contrast, Cp190 was largely dispensable for activation of HOX genes by distal enhancers (Fig. 6 and figs. S6 and S7). (viii) Cp190 is similarly only critical for the enhancer-blocking activity of the classical Homie insulator but not for Homie pairing. Below, we discuss how this work advances our understanding of how contact domain boundaries are formed and affect transcriptional regulation.

### Diversity of boundary-forming mechanisms

*Drosophila* contact domains frequently align with active/inactive compartmental domains, raising the question of whether fly contact domains are formed directly by architectural proteins assembled at boundaries or indirectly by transcription-related processes (*1*, *7*, *65*). Our studies of *Drosophila* completely lacking Cp190 or CTCF demonstrate that these proteins form a subset of domain boundaries that are distal to sites of transcription (Figs. 2 and 3) (*7*). At least two distinct mechanisms of boundary formation therefore exist, one relying on architectural proteins and the other correlating with transcribed promoters (*1*, *66*).

Part of this study focused on CTCF peaks and revealed three lines of evidence that Cp190 promotes boundary formation at CTCF peaks: (i) Only CTCF peaks colocalizing with Cp190 are present at domain boundaries (Fig. 2A). (ii) Most CTCF-occupied boundaries are lost or weakened in *Cp190^0^*, although CTCF remains bound (Figs. 2 and 3). (iii) Residual Cp190 binding at former CTCF-occupied boundaries coincides with boundary retention in *CTCF^0^* mutants (Fig. 3) (*7*). We do not know why some CTCF-occupied boundaries were unexpectedly reinforced in *CTCF^0^* mutants relative to WT (Fig. 3, A and C). Even boundaries not bound by CTCF were stronger in *CTCF^0^* relative to WT (fig. S2A). A hypothetical explanation is that boundary strength is somehow redistributed to the remaining Cp190-dependent boundaries in the absence of CTCF.

To summarize, whereas CTCF forms a large fraction of mammalian contact domains by directly blocking extruding cohesin, our results support the notion that flies use Cp190 as an adaptor protein recruited DNA sequence-specifically by proteins such as CTCF and Su(Hw) to form robust physical boundaries at these sites. Cp190 is therefore more widely required for boundary formation than individual DNA binding proteins such as CTCF.

Seventy-eight percent of contact domain boundaries are retained in *Cp190^0^* mutants (Fig. 1B). How are these boundaries formed? (i) At promoter boundaries, it is still unclear what drives boundary formation: transcription itself, active chromatin modifications, RNA polymerase II, promoter-associated factors, or insulator proteins at promoter boundaries such as BEAF-32 (*1*, *46*, *66*, *67*). Our results do not support a model in which Cp190 drives promoter boundary formation (*24*). (ii) At nonpromoter boundaries such as SF1 that was partially retained in *Cp190^0^*, deletion of SF1 boundary DNA more strongly increased contacts between flanking contact domains than Cp190 loss (Fig. 5B), revealing that Cp190 is less important than other SF1-associated factors to form this boundary.

### Cp190 prevents promiscuous gene regulation at tested loci

We previously reported that CTCF and Cp190 co-regulate a subset of genes near CTCF-dependent boundaries (*7*). We did not know which regulatory elements were driving gene misexpression and hence could not say whether misregulation arose from regulatory cross-talk between formerly insulated loci. By assessing how Cp190 loss affects gene expression in the best-characterized *Drosophila* developmental loci, we found that *Scr* was ectopically expressed in patterns that could be predicted on the basis of annotated enhancers located up to 30 kb away from its promoter (Figs. 5 and 6). *Scr* misexpression patterns in *Cp190^0^* evolved dynamically during embryogenesis, reflecting changing enhancer activities. *Scr* was ectopically activated by *ftz* enhancers despite retention of *ftz* contact domain boundaries.

These results, together with our findings that all tested Cp190 binding sites exert insulator activity in a reporter assay (Fig. 4), and that Cp190 is required for efficient Homie enhancer-blocking activity (Fig. 7), all consolidate the original notion that Cp190 is critical for gene insulation. A well-understood function of genetic insulators is the regulation of abdominal HOX gene expression along the anterior-posterior body axis by ensuring that segment-specific regulatory domains containing HOX gene enhancers and silencers act independently (*61*, *68*). These HOX insulators coincide with contact domain boundaries between regulatory domains, and boundary deletion results in contact domain fusion (*65*). We were therefore surprised that expression of abdominal HOX genes and contact domain boundaries themselves were mildly affected in *Cp190^0^* mutants compared to such boundary deletions (fig. S7) (*63*, *64*). Moreover, *Abd-B* is misexpressed in *CTCF^0^* but not *Cp190^0^* embryos (fig. S7B) (*69*). This indicates that other factors are able to exert genetic insulation independently of Cp190 at abdominal HOX gene boundaries. It also remains to be determined how widely Cp190 protects other genes from inappropriate regulation.

### Cp190 is not essential for HOX gene and Homie-mediated distal activation

*Drosophila* insulators are traditionally thought to form chromosomal loops exerting seemingly contradictory effects, both blocking and facilitating regulatory element-promoter communication by respectively segregating or connecting these elements. SF1 and SF2B boundary pairing was thus proposed to shield *Scr* from *ftz* enhancers and bridge *Scr* to its putative distal enhancer (*58*, *59*). We instead found that both Cp190 and SF1-SF2B boundary pairing are dispensable for *Scr* embryonic expression (Fig. 5C). The putative distal *Scr* enhancer is located 25 kb upstream of the *Scr* promoter (fig. S5B), and its relevance for *Scr* transcriptional activation remains uncertain, but we find that an enhancer located even further away (30 kb downstream of the *Scr* promoter) is able to activate *Scr* transcription in *Cp190^0^* mutants (Fig. 6). Our finding that abdominal HOX genes are also expressed in patterns normally driven by their long-range enhancers in *Cp190^0^* mutants further suggests that Cp190 is not essential for long-distance enhancer-promoter pairing at these loci. Consistently, the abilities of abdominal HOX boundaries to support long-distance activation of HOX promoters by their distal enhancers was recently suggested to rely on uncharacterized factors other than insulator proteins (*70*).

We further demonstrate that Cp190 is dispensable for Homie’s ability to mediate transcriptional activation by distal enhancers 142 kb away (Fig. 7). We suggest that Cp190 is not a “looping factor” critical for distal enhancer-promoter pairing. However, we have not assessed the relevance of Cp190 for fostering enhancer-promoter communication at other loci and thus cannot exclude that Cp190 supports long-range regulation of other genes, for example, within a contact domain by bringing promoters and their cognate enhancers into enhanced three-dimensional proximity.

### Molecular basis of Cp190 function

CTCF, Su(Hw), and BEAF-32 colocalize with Cp190 at only a subset of their respective binding sites (Fig. 2A) (*30*, *42*). Whether Cp190 colocalization with these proteins is regulated or instead dictated by the underlying DNA sequence is debated (*29*, *30*, *40*, *42*, *71*). We did not detect differentially enriched DNA motifs in our set of embryonic CTCF standalone versus CTCF+Cp190 cobound sites, and we do not know why CTCF standalone sites have intermediate ChIP occupancy in embryos (Fig. 2A). Standalone sites may exert different activities than sites cobound by Cp190. By testing a few Su(Hw) sites in a transgenic insulator assay, Su(Hw) standalone sites were proposed to be repressors unlike Su(Hw)+Cp190 cobound sites (*30*). Similarly, CTCF standalone sites were proposed to lack insulator activity unlike some CTCF+Cp190 cobound sites (*30*). We now show that Cp190 imparts physical boundary activity to sites to which it is recruited by CTCF or Su(Hw) (Fig. 2A and fig. S4, B and C). This activity may underlie co-regulation of some genes near CTCF-dependent boundaries by both CTCF and Cp190 (*7*).

How does Cp190 form boundaries? Two main models were proposed to explain how insulator proteins fold chromosomes: (i) by pairwise looping between contact domain boundaries or (ii) by stalling loop-extruding cohesin at contact domain boundaries. The first model originally proposed that Cp190 interacts with DNA-bound insulator proteins through its C-terminal domain and dimerizes with distal Cp190-bound sites via its BTB domain (*33*, *39*). It later became clear, however, that Cp190 BTB interacts directly with, for example, CTCF and Su(Hw) (*7*, *72*). On the other hand, whether insulator proteins stall loop-extruding cohesin in flies is still debated (*7*), and cohesin has not yet been shown to play a major role in fly contact domain formation (*1*, *46*). We find that both CTCF and Cp190 copurify with cohesin, but we do not know whether Cp190 interacts with cohesin independently of CTCF (*7*).

Not all CTCF binding sites in mammalian cells are associated with physical boundaries, and the contribution of non-CTCF proteins to boundary reinforcement has recently been explored (*73*). Our finding that Cp190 is recruited to DNA-bound CTCF to reinforce boundaries in *Drosophila* highlights that it will be interesting to further investigate whether analogous mechanisms are deployed across species and locus-specifically within a species.

## MATERIALS AND METHODS

### *Drosophila melanogaster* crosses

Using the same genetic strategy used to generate *CTCF^0^* (*69*) and *Cp190^0^* (*7*) mutants, *double^0^* mutants were generated for this study by recombining knockout mutations of the entire open reading frames of *CTCF* and *Cp190* and rescuing the double knockout animals by excisable FRT (flippase recognition target)-flanked genomic *CTCF* and *Cp190* rescue fragments. These rescue fragments were excised from the germ lines of conditionally rescued mothers and fathers expressing FLP (Flippase) under the control of *nanos* regulatory sequences. WT embryos with a matched genetic background were used as control in all Hi-C, NG Capture-C, and ChIP-seq experiments. Similar to *double^0^* embryos, WT embryos were generated by excising the same FRT-flanked genomic *CTCF* and *Cp190* rescue fragments from the germ lines of mothers and fathers expressing FLP enzyme under the control of *nanos* regulatory sequences, but these flies were WT for *CTCF* and *Cp190*.

*su(Hw)^KO^*, *SF1^KO^*, and *SF2B^KO^* mutants were generated by CRISPR-Cas9–mediated genome editing using two single guide RNAs flanking the regions chosen for deletion: 3157 bp of the entire *su(Hw)* open reading frame (dm6 coordinates chr3R:14307954-14304798) for *su(Hw)^KO^*, 2041 bp (dm6 coordinates chr3R:6853644-6855684) for *SF1^KO^*, or 2122 bp (dm6 coordinates chr3R:6869630-6871751) for *SF2B^KO^*. Guide RNAs were cloned into pCFD3 (Addgene, 49410). One-kilobase left and right homology arms were cloned into pHD-DsRed-attP vector (Addgene, 51019) for homology-directed repair leading to the integration of a *DsRed* fluorescent selection marker in each knockout allele. Primers used for cloning guide RNAs and homology arms of the donor plasmid are provided in table S2. Both guide RNA plasmids and the homology repair plasmid were injected into flies expressing Cas9 in their germ line (*nanos-Cas9*).

Homie and control transgenes inserted 142 kb upstream of *eve* originally described in figure 3 of Fujioka *et al.* (*37*) were introduced into the *Cp190^0^* mutant background by recombining them onto the same second chromosome also harboring the FLP transgene.

### *Drosophila* viability tests

Three sets of between 60 and 90 embryos of desired genotypes were aligned on a glass coverslip and vertically inserted into a fly culture vial. Vials were placed at 25°C, and unfertilized eggs and hatched larvae were counted 2 days later. The vials were later scored for the numbers of pupae and adult flies that completely emerged from the pupal case. The numbers of hatched embryos, pupae, and adults were counted in the triplicate experiments for each genotype.

### Western blotting

For Western blotting presented in fig. S1A, 6- to 10-hour embryos were dechorionated, homogenized in SDS sample buffer, sonicated for 10 cycles (30 s on and 30 s off) in a Bioruptor on high-intensity settings, and centrifuged. The supernatants were loaded on a 4 to 12% acrylamide gel and probed with rabbit anti-CTCF-N diluted 1:2000, rabbit anti–full-length Cp190 diluted 1:2000 (*7*), and mouse anti-tubulin clone B-5-1-2 (Sigma-Aldrich, T5168) diluted 1:10,000. Chemiluminescence pictures of nitrocellulose membranes were imaged in Fiji v2.1.0/1.53c.

For Western blotting presented in fig. S2G, 40 third-instar larval central nervous systems per biological replicate were dissected in ice-cold phosphate-buffered saline (PBS). Samples were sonicated in 100 μl of 20 mM tris (pH 7.5), 500 mM NaCl, 0.1% Triton X-100, and 1× cOmplete protease inhibitors (Roche) in a Bioruptor on high-intensity settings for 5 min at 4°C. Extracts were centrifuged for 5 min at maximum speed, and total protein was quantified by Qubit protein assay (Thermo Fisher Scientific). Calibrated amounts of extract from *CTCF^0^* animals rescued by TAP (Tandem Affinity Purification)-tagged transgenic versions CTCF^WT^, CTCF^ΔN^, or CTCF^ΔC^ were loaded on a 4 to 12% acrylamide gel and probed with rabbit peroxidase anti-peroxidase antibody complex (Sigma-Aldrich, P1291) diluted 1:2000 and mouse anti-tubulin clone B-5-1-2 (Sigma-Aldrich, T5168) diluted 1:10,000.

### Chromatin preparation from fly embryos

Approximately 400 0- to 14-hour-old embryos per biological replicate (three biological replicates prepared per genotype) were dechorionated in bleach diluted 1:1 in water for 2 min at room temperature, extensively rinsed with water, transferred to an Eppendorf, flash-frozen, and stored at −80°C. Embryos were homogenized in a glass 15-ml Dounce in 5 ml of cross-linking solution [50 mM Hepes (pH 7.9), 1 mM EDTA (pH 8), 0.5 mM EGTA (pH 8), 100 mM NaCl, and 1.8% formaldehyde] with 15 strokes, transferred to a 15-ml Falcon tube, and rotated at room temperature. Cross-linking was stopped after 15 min by pelleting nuclei for 2 min at 2000*g* and rotating for 10 min in stop solution (1× PBS, 125 mM glycine, and 0.01% Triton X-100). Nuclei were washed for 10 min in solution A [10 mM Hepes (pH 7.9), 10 mM EDTA (pH 8), 0.5 mM EGTA (pH 8), and 0.25% Triton X-100] and then for 10 min in solution B [10 mM Hepes (pH 7.9), 1 mM EDTA (pH 8), 0.5 mM EGTA (pH 8), 0.01% Triton X-100, and 200 mM NaCl]. Nuclei were sonicated in 100 μl of radioimmunoprecipitation assay (RIPA) buffer [10 mM tris-HCl (pH 8), 140 mM NaCl, 1 mM EDTA (pH 8), 1% Triton X-100, 0.1% SDS, 0.1% sodium deoxycholate, and 1× cOmplete protease inhibitor cocktail] in AFA microtubes in a Covaris S220 sonicator for 5 min with a peak incident power of 140 W, a duty cycle of 5%, and 200 cycles per burst. Sonicated chromatin was centrifuged to pellet insoluble material and snap-frozen.

### Chromatin preparation from larval central nervous systems

Thirty third-instar larval cuticles per biological replicate (two biological replicates per sample) were dissected in ice-cold PBS and then cross-linked for 15 min at room temperature in 1.8% (v/v) paraformaldehyde, 50 mM Hepes (pH 8), 100 mM NaCl, 1 mM EDTA, and 1 mM EGTA. Cross-linking was stopped by washing for 10 min in 1 ml of PBS, 0.01% Triton X-100, and 125 mM glycine. Then, cuticles were washed for 10 min in 10 mM Hepes (pH 7.6), 10 mM EDTA, 0.5 mM EGTA, and 0.25% Triton X-100. Central nervous systems were dissected from the cuticles in 10 mM Hepes (pH 7.6), 200 mM NaCl, 1 mM EDTA, 0.5 mM EGTA, and 0.01% Triton X-100 and then sonicated in 100 μl of RIPA buffer [10 mM tris-HCl (pH 8), 140 mM NaCl, 1 mM EDTA, 1% Triton X-100, 0.1% SDS, 0.1% sodium deoxycholate, and protease inhibitor cocktail] in AFA microtubes in a Covaris S220 sonicator for 5 min with a peak incident power of 140 W, a duty cycle of 5%, and 200 cycles per burst. Sonicated chromatin was centrifuged to pellet insoluble material and snap-frozen.

### ChIP-seq

ChIP was performed with 2 μl of rabbit polyclonal antibody crude sera against CTCF^1-293^ or Cp190^1-1096^ (*7*) each incubated with half of the chromatin prepared from a biological replicate overnight at 4°C. Premixed Protein A and G Dynabeads (25 μl; Thermo Fisher Scientific, 100-01D and 100-03D) were added for 3 hours at 4°C and then washed for 10 min each once with RIPA, four times with RIPA with 500 mM NaCl, once in LiCl buffer [10 mM tris-HCl (pH 8), 250 mM LiCl, 1 mM EDTA, 0.5% IGEPAL CA-630, and 0.5% sodium deoxycholate], and twice in TE buffer [10 mM tris-HCl (pH 8) and 1 mM EDTA]. DNA was purified by ribonuclease digestion, proteinase K digestion, reversal of cross-links at 65°C for 6 hours, and elution from a QIAGEN MinElute PCR (polymerase chain reaction) purification column. ChIP-seq libraries were prepared using the NEBNext Ultra II DNA Library Prep Kit for Illumina. An equimolar pool of multiplexed ChIP-seq libraries at 4 nM was sequenced on two lanes of an Illumina HiSeq 4000 150-bp paired-end.

### ChIP-seq analysis

Paired-end ChIP-seq reads were demultiplexed and mapped to the dm6 genome using micmap v2.20200223 (https://github.com/sib-swiss/micmap), a derivative of the fetchGWI tool. For samples that were sequenced twice, reads were merged with Samtools v1.10 (http://www.htslib.org/). Only chromosomes 2, 3, 4, and X were used. ChIP-seq peaks were called using the R package csaw v1.16.1 (https://bioconductor.org/packages/release/bioc/html/csaw.html) using a window width of 20 bp and spacing of 10 bp, ignoring duplicate reads, and blacklisted regions by ENCODE. A background enrichment was evaluated as the median over all samples in the comparison of the average number of reads per 2-kb bins. Windows with less than twofold (for ChIP-seq in embryos) or threefold (for ChIP-seq in larval central nervous systems, which gives better signal-to-noise ratio) enrichment over background were filtered out. Data were normalized using the TMM (trimmed mean of M-values) method implemented in csaw. Differential binding analysis in csaw is based on the quasi-likelihood framework implemented in the edgeR package v3.22.5 (https://bioconductor.org/packages/release/bioc/html/edgeR.html). Results obtained on different windows were combined into regions by clustering adjacent windows. Combined *P* values were evaluated for each region using csaw, and the Benjamini-Hochberg method was applied to control the false discovery rate. Regions with false discovery rate <0.01 and |best.logFC| > 1 were considered as differentially bound regions. Genuine Cp190 peaks were identified by differential analysis of ChIP-seq signals in WT versus *Cp190^0^* embryos (Cp190 peaks in WT embryos; data S1), in *CTCF^0^* versus *Cp190^0^* embryos (Cp190 peaks in *CTCF^0^* embryos; data S6), in WT versus *Cp190^KO^* larval central nervous systems (Cp190 peaks in WT larval central nervous system; data S10), or in *su(Hw)^KO^* versus *Cp190^KO^* larval central nervous systems [Cp190 peaks in *su(Hw)^KO^* larval central nervous system; data S11] as being lower in *Cp190* mutants relative to WT, *CTCF^0^*, or *su(Hw)^KO^*, respectively. Genuine CTCF peaks were similarly identified by differential analysis of ChIP-seq signals in WT versus *CTCF^0^* embryos (CTCF peaks in WT embryos; data S5) or in *Cp190^0^* versus *CTCF^0^* embryos (CTCF peaks in *Cp190^0^* embryos; data S8) as being lower in *CTCF^0^* relative to WT or *Cp190^0^*, respectively. One replicate of CTCF ChIP in *Cp190^0^* embryos failed at the library preparation step; hence, differential analysis was performed with the two remaining replicates. Additional differential analyses were performed for Cp190 ChIP-seq in WT versus *CTCF^0^* embryos (data S7), for CTCF ChIP-seq in WT versus *Cp190^0^* embryos (data S9), and for Cp190 ChIP-seq in WT versus *su(Hw)^KO^* larvae (data S12). Throughout the manuscript, the following conventions are used when comparing ChIP data to other data. ChIP occupancy was defined as the best.log2FC obtained from csaw in the respective differential analysis. ChIP peak (and differentially bound region) positions were defined as the best.pos obtained from csaw, and regions were defined as the [start,end] interval obtained from csaw. Overlapping ChIP peaks (and differentially bound regions) were defined as those with peak regions sharing at least 1 bp. Similarly, ChIP peaks overlapping a DNA motif were defined as those with peak regions sharing at least 1 bp with the motif. CTCF- or Cp190-occupied boundaries were defined as those with a ChIP peak position within ±2 kb of the boundary. Promoter-proximal and promoter-distal ChIP peaks were defined as those with peak positions within ±200 bp or further away from the closest TSS, respectively. In fig. S2D, CTCF peaks were defined as present in an intron when the peak position was inside an intron but not in an exon using gene annotations from FlyBase release FB2020_06.

### Hi-C

About 100 2- to 6-hour-old embryos per biological replicate (four biological replicates per genotype) were dechorionated in bleach diluted 1:1 in water for 2 min at room temperature, extensively rinsed with water, transferred to an Eppendorf, and crushed in RPMI supplemented with 10% fetal bovine serum using a micropestle. Nuclei were fixed in 1% (v/v) formaldehyde for 10 min at room temperature. Cross-linking was stopped by adding 200 mM glycine; then, nuclei were washed in PBS and snap-frozen for −80°C storage. Nuclei were restricted with Mse I and Csp 6I; restricted ends were marked with biotin and then ligated. DNA was purified by proteinase K digestion and reverse cross-linking at 65°C for 6 hours, then sonicated in AFA microtubes in a Covaris S220 sonicator, and purified on SPRIselect beads (Beckman Coulter). DNA was end-repaired, A-tailed, and ligated to barcoded adapters using the NEBNext Ultra II DNA Library Prep Kit for Illumina and then enriched for pairwise DNA junctions by biotin pull-down using Dynabeads MyOne Streptavidin T1 beads following the manufacturer’s instructions. Libraries were amplified using KAPA HiFi HotStart Ready Mix and purified on SPRIselect beads. Four nanomolar equimolar pools of multiplexed Hi-C libraries were subjected to 150-bp paired-end sequencing on HiSeq 4000 instruments.

### Hi-C analysis

We precomputed a table containing the positions of all restriction sites used for Hi-C present in the dm6 genome. The FASTQ read pairs were analyzed with a Perl script available for download in the micmap package v2.20200223 (https://github.com/sib-swiss/micmap) to locate and separate fusion sites using the patterns /GTATAC/, /TTATAA/, /GTATAA/, and /TTATAC/. The maximal length of each read was trimmed at 60 nucleotides (nt); then, reads were mapped to the dm6 genome using micmap and matched to their closest precomputed genomic restriction site. Read pairs were discarded if they (i) mapped to non-unique positions in the reference genome; (ii) had indels or more than two mismatches per read; (iii) represented fusion of two oppositely oriented reads within 2 kb of each other, which may have not resulted from ligation of two digested fragments; and (iv) were likely PCR duplicates. Only chromosomes 2, 3, 4, and X were considered, and chromosome arms were treated as separate chromosomes.

To assess correlation of biological replicates, samples were downsampled to 13 million contacts per replicate. Raw Hi-C contact matrices were created by binning Hi-C pairs at 10-kb resolution. These matrices were then normalized with the ICE (iterative correction and eigenvector decomposition) normalization implemented in iced v0.5.2 (https://github.com/hiclib/iced). Low-coverage regions (bins with no contacts and those with the 5% smallest total number of contacts among bins) were filtered out before normalization. Pearson correlation coefficients were determined for every pair of normalized matrices by flattening each matrix and evaluating the Pearson correlation coefficient for the resulting vector using only pairs of bins at a genomic distance below 1 Mb. The limitation on the distance was introduced to compare contacts at a scale relevant to the analyses performed in this manuscript, which were at the level of contact domains. Resulting Pearson correlation coefficients were ≥0.936 for all replicates, showing that they were well correlated and that WT and mutant Hi-C matrices were globally similar. For the analyses presented in the main figures, pooled quadruplicate replicates of the same genotype were downsampled to 79 million contacts per genotype. Raw Hi-C contact matrices obtained by binning Hi-C pairs at 2-kb resolution were then normalized with the ICE normalization implemented in iced v0.5.2. Low-coverage regions (bins with no contacts and 5% of bins with the smallest total number of contacts but at least one contact) were filtered out before normalization (these regions are marked by gray lines in Hi-C maps shown in the figures). Hi-C maps were visualized in R.

For each normalized Hi-C contact matrix, contact domain boundaries were called using TopDom v0.0.2 (https://github.com/jasminezhoulab/TopDom) (*41*). Given a window size *w*, a physical insulation score was defined for each bin *i* as

$$\text{log}2\frac{{\text{binSignal}}_{i}}{\sum_{i-w/2<j<i+w/2}{\text{binSignal}}_{j}}$$ (1)

where binSignal*_i_* is the average normalized Hi-C contact frequency between *w* bins upstream of bin *i* and *w* bins downstream of bin *i* determined by TopDom. The strength of a boundary at bin *i* was thus estimated as the log_2_ of the binSignal value at bin *i* normalized by its local average on a window of size *w*. With this definition, lower physical insulation scores indicate stronger boundaries. We extracted contact domain boundaries and physical insulation scores for Hi-C matrices at 2-kb resolution using window sizes 20, 40, 80, and 160 kb. Contact domain boundaries found with all window sizes were merged (boundaries with an insulation score >−0.1 were ignored), and the average insulation score obtained with all window sizes was retained. Boundaries with an average insulation score >−0.1 were filtered out. To facilitate comparisons of contact domain boundaries between genotypes and avoid mismatches due to small fluctuations of domain boundary positions obtained with different window sizes or genotypes, groups of consecutive boundaries (i.e., within 2 kb of each other) found in any of the four genotypes (WT, *Cp190^0^*, *CTCF^0^*, and *double^0^*) were replaced by the boundary with the lowest global insulation score (sum of insulation scores over all genotypes having this boundary), with insulation score taken from the corresponding genotype at the new boundary position. Boundaries in blacklisted regions were also filtered out to have a set of boundaries comparable to the set of ChIP peaks.

### A/B compartment calling

A/B compartment calling was performed following the method proposed by Lieberman-Aiden *et al.* (*74*). Each individual chromosome arm (chr2L, chr2R, chr3L, chr3R, chr4, and chrX) was analyzed separately. In addition, to avoid having eigenvectors dominated by a chromosomal rearrangement in chr2L present in all four genotypes (WT, *Cp190^0^*, *CTCF^0^*, and *double^0^*), chr2L was split into three subregions analyzed independently (region 1 < 9471500, 9471500 < region 2 < 13657500, and region 3 > 13657500). Normalized Hi-C contact matrices at 2-kb resolution were considered after discarding invalid bins (low-coverage regions filtered before ICE normalization) and bins around centromeres (chosen for exclusion as dm6 coordinates >22170000 for chr2L, <5650000 for chr2R, >22900000 for chr3L, and <4200000 for chr3R). Observed-over-expected matrices were generated by dividing the normalized Hi-C contact matrices by the average number of normalized Hi-C contacts at the corresponding genomic distance and clipped to the 99.9th percentile to avoid instabilities due to very large values. For each chromosome arm or chr2L region, the first eigenvector of the correlation matrix was obtained by principal components analysis of the observed-over-expected matrix. Each eigenvector was then multiplied by the sign of the Spearman correlation between the eigenvector and the number of gene TSSs per 2-kb bin (gene list taken from FlyBase release FB2020_06; ftp://ftp.flybase.net/genomes/Drosophila_melanogaster_dmel_r6.37_FB2020_06/gtf/dmel-all-r6.37.gtf.gz) and then centered around zero by subtracting its mean value. For chr2L, centering around zero was performed after merging eigenvectors from all regions. Two-kilobase bins with positive eigenvector values were assigned to compartment A; those with negative eigenvector values were assigned to compartment B. chr4 first eigenvector failed to capture the relevant A/B compartment structure and was thus excluded from fig. S1C.

### Capture-C

The NG Capture-C (*60*) protocol was adapted as follows. Hi-C was performed exactly as described above on 2- to 6-hour-old WT, *Cp190^0^*, and *SF1^KO^* embryos in biological triplicates. Five hundred nanograms of each of the nine Hi-C libraries (4.5 μg total DNA) was multiplexed and hybridized with 2.5 μg of Cot DNA, 2.5 μg of salmon sperm, 2 nmol of blocking oligos, and 16 fmol of xGen Lockdown probe pool [Integrated DNA Technologies (IDT)] consisting of 5′-biotinylated 120-nt single-stranded DNA capture probes listed in table S3 complementary to both ends of each of the 10 restriction fragments selected as viewpoints. Hybridization (24 hours at 65°C) and washes were performed with the xGen Hybridization and Wash Kit (IDT, 1080577) according to the manufacturer’s protocol. The first capture was PCR-amplified with KAPA HiFi HotStart ReadyMix in a 50-μl reaction with 14 cycles to obtain 1 μg of postcapture DNA. This DNA was subsequently subjected to a second capture identical to the first, after which only nine PCR cycles were necessary to obtain sequencing-ready DNA. Capture-C libraries were subjected to 150-bp paired-end sequencing on a NovaSeq 6000.

### Capture-C analysis

As for Hi-C analysis, we precomputed a table containing the positions of all restriction sites used for Hi-C present in the dm6 genome. To extract the read pairs corresponding to each captured region (called viewpoint), all capture probe sequences were split into consecutive 25-mers, and a Perl script was used to scan all read pairs in the raw FASTQ files and generate a specific pair of FASTQ files per viewpoint in which either read of the pair had an exact match to one of the 25-mers. The FASTQ read pairs were mapped to the dm6 genome using STAR (v2.7.7a; https://github.com/alexdobin/STAR) with parameters tuned to map the expected chimeric read pairs generated by Hi-C (--chimOutType WithinBAM, --chimSegmentMin 10, --outFilterMultimapNmax 1, --outFilterMismatchNoverLmax 0.04, --scoreGapNoncan 0, --scoreGapGCAG 0, --scoreGapATAC 0, --alignIntronMax 1, --chimScoreJunctionNonGTAG 0). Each matching pair was assigned to their closest precomputed genomic restriction site. Only chimeric read pairs (as defined by STAR) were retained.

For each viewpoint per sample, only informative read pairs were considered, i.e., only unique read pairs (after discarding probable PCR duplicates) with at least one read mapping to the viewpoint restriction fragment or one of its two neighboring restriction fragments. A vector of raw counts between the viewpoint and other regions in the genome was obtained by partitioning the genome into 1-kb bins (rounded to the nearest restriction site) and evaluating, for each bin, the number of read pairs with one end associated with the viewpoint and the other end associated with a restriction fragment overlapping the bin. Bins located <2 kb or >100 kb from the viewpoint restriction fragment were discarded.

For each comparison (*Cp190^0^* versus WT in data S14 and *SF1^KO^* versus WT in data S15), a differential analysis was done with diffHic v1.14.0 (https://www.bioconductor.org/packages/release/bioc/html/diffHic.html) using vectors of raw counts for all viewpoints. Data were normalized by library size. The Benjamini-Hochberg method was applied to control the false discovery rate. Capture-C read pair quality metrics are shown in table S4.

### Analysis of published datasets

RNA-seq data in 4- to 6-hour-old WT embryos [mE_mRNA_em4-6hr_(FBIc0000088)] (*75*) were downloaded from FlyBase release FB2020_06 (http://ftp.flybase.org/releases/FB2020_06/precomputed_files/genes/gene_rpkm_matrix_fb_2020_06.tsv.gz). Reads per kilobase per million reads (RPKM) values were calculated only for the unique exonic regions of the gene (excluding segments that overlap other genes), except for genes derived from di- or polycistronic transcripts, in which case, all exons were used in the RPKM expression calculation. For visualizing TSSs mapped by PRO-seq (Precision Run-On Sequencing) in 3- to 4-hour-old WT embryos and CAGE (cap analysis of gene expression) in 2- to 4-hour-old WT embryos (*76*) in fig. S1D, these published datasets were lifted over from dm3 to dm6 coordinates using the CrossMap tool (v0.6.0; https://crossmap.readthedocs.io/en/latest/). These datasets contain signed counts of 5′ ends of reads per base pair (with positive/negative values corresponding to reads on the positive/negative strand), and only the absolute values of the counts were plotted in fig. S1D.

Published ChIP-seq profiles in S2 or in Kc cells when not available in S2 cells were remapped to genome version dm6 using the same pipeline described above for ChIP-seq analysis and visualized in Fig. 4B: Ibf1 (SRR837792) and Ibf2 (SRR837793) in S2 cells from GSE47559 (*31*); CTCF (SRR580343), Su(Hw) (SRR580339), mod(mdg4) (SRR580341), and Cp190 (SRR580337) in S2 cells from GSE41354 (*40*); BEAF-32 (SRR1042411) in S2 cells from GSE52962 (*33*); ZIPIC (SRR1141009) in S2 cells from GSM1313421 (*32*); and Pzg (SRR1636808) and Chro (SRR1636762) in Kc cells from GSE63518 (*46*).

To assess whether contact domain boundaries called in our study overlap those of previously published Hi-C contact maps, we compared our boundaries to those called by Hug *et al.* (*67*) using 3- to 4-hour-old WT embryo Hi-C maps binned at 2-kb resolution and by Ramírez *et al.* (*8*) using Kc167 tissue culture cell Hi-C maps binned at Dpn II restriction fragment resolution. Boundaries from Ramírez *et al.* (*8*) were converted from dm3 to dm6 genome coordinates using the LiftOver tool (http://genome.ucsc.edu/cgi-bin/hgLiftOver). Published boundaries are displayed together with those from this study in all Hi-C screenshots in the manuscript for comparison.

To visualize and assess boundary defects around DNA motifs, we used known motifs [JASPAR motifs MA0531.1 for CTCF, MA0533.1 for Su(Hw), MA0529.2 for BEAF-32, and MA1459.1 for M1BP or published core motif-6 from Ohler *et al.* (*77*)]. The Ibf1 and ZIPIC motifs were rediscovered from the published ChIP-seq datasets mentioned above. For each ChIP-seq dataset, peaks were called with MACS2, the 500 peaks with the highest scores were selected, and sequences ±100 bp around the ChIP peak summits were extracted and submitted to MEME (https://meme-suite.org/meme/tools/meme). The Ibf2 motif was similarly also rediscovered and was almost identical to that of Ibf1 as expected (*31*); hence, we only used the Ibf1 motif in Fig. 1 and fig. S1. The genome was then scanned for occurrences of each considered motif using PWMScan (https://ccg.epfl.ch/pwmtools/pwmscan.php) with default parameters. For visualizing deoxyribonuclease (DNase) hypersensitive sites mapped by DNase sequencing in 4- to 6-hour-old whole WT embryos (*78*) in fig. S1D, this published dataset was lifted over from dm3 to dm6 coordinates using the LiftOver tool.

### RNA–fluorescence in situ hybridization

For single RNA–fluorescence in situ hybridization (FISH), labeled RNA probes were generated by in vitro transcription with Dig-UTP (digoxigenin-11-uridine triphosphate) labeling mix (Roche, 11277073910) and T7 RNA polymerase (Roche, 10881767001) antisense to full-length cDNA clones of *Scr*, *Dfd*, *Antp* (LD33666), *Ubx* (RE43738), *abd-A* (RE04174), *Abd-B* (RE47096), and *ftz* [dm6 coordinates chr3R:6864324-6865765 as originally published by Yokoshi *et al.* (*59*)] (see table S2 for primer sequences). After DNase I digestion for 20 min at 37°C, probes were fragmented by incubating for 20 min at 65°C in 60 mM Na_2_CO_3_ and 40 mM NaHCO_3_ (pH 10.2); precipitated in 300 mM sodium acetate (pH 5.2), 1.25 M LiCl, tRNA (50 mg/ml), and 80% ethanol; resuspended in 50% formamide, 75 mM sodium citrate (pH 5), 750 mM NaCl, salmon sperm DNA (100 μg/ml), heparin (50 μg/ml), and 0.1% Tween 20; and stored at −20°C. Embryos were fixed in 4% paraformaldehyde for 30 min at room temperature, washed, and then stored in 100% methanol at −20°C. Samples were rehydrated in PBS with 0.1% Tween 20, postfixed in 4% paraformaldehyde for 20 min at room temperature, progressively equilibrated to hybridization buffer [50% formamide, 75 mM sodium citrate (pH 5), and 750 mM NaCl], and heated to 65°C. RNA probes were diluted 1:50 in hybridization buffer, denatured at 80°C for 10 min, then placed on ice, and added to the samples for overnight shaking at 65°C. Samples were washed six times for 10 min in hybridization buffer at 65°C and then progressively equilibrated to PBS with 0.1% Triton X-100. Samples were incubated overnight at 4°C in anti-Dig peroxidase (Roche, 11207733910) diluted 1:2000 in PBS, 0.1% Triton X-100, and 1× Western blocking reagent (Sigma-Aldrich, 1921673). Samples were washed six times for 10 min in PBS with 0.1% Tween 20, labeled with Cyanine 3 Tyramide in the TSA Plus kit (PerkinElmer, NEL753001KT) for 3 min at room temperature, washed six times for 10 min in PBS with 0.1% Tween 20, and lastly mounted with 4′,6-diamidino-2-phenylindole (DAPI) to stain DNA. Images were acquired on a Zeiss LSM 880 microscope with a ×20 objective and visualized with Fiji software v2.1.0/1.53c.

For double RNA-FISH, we used MUSE technology (arcoris bio). Twenty probes with around 30 bases antisense to the mRNA of interest (*Scr* or *ftz*; complementary sequences in table S2) were ordered with MUSE overhangs from IDT. Equimolar pools containing 100 nM each probe (100× stock) were made in 2× SSC (pH 7; 300 mM NaCl and 30 mM sodium citrate) and stored at −20°C. Embryos were fixed, stored, and rehydrated as for single RNA-FISH. Probe pools were diluted 100× and incubated with embryos at 40°C overnight. Samples were washed six times for 10 min in hybridization buffer at 40°C and then progressively equilibrated to and washed in 2× SSCT [2× SSC (pH 7) and 0.1% Tween 20]. MUSE signal was then detected by hybridizing nanoamplifiers and ATTO-488 and ATTO-550 readout probes according to the manufacturer’s instructions. Embryos were DAPI-stained and mounted on microscope slides in 2× SSC for immediate imaging on a Zeiss LSM 880 microscope with a ×20 objective. Images were processed with Fiji software v2.1.0/1.53c.

### Insulator reporter

An insulator reporter plasmid (Fig. 4C) (*7*) comprises an enhancer (*OpIE2*) equidistant from EGFP and mCherry fluorescent reporters with basal *Hsp70* promoters. A *gypsy* insulator is present in the reporter plasmid, downstream of the EGFP transcription unit. Selected genomic loci were PCR-amplified using primer sequences in table S2 from genomic DNA and cloned in between the enhancer and EGFP. Control reporters had a neutral spacer (a fragment of the bacterial *Kanamycin* resistance gene) or the *gypsy* insulator in between the enhancer and EGFP. In addition, genomic fragments with BEAF-32 motifs were mutagenized by PCR to mutate 1 bp in a BEAF-32 motif (TATCGATW to TAGCGATW). All plasmids of a given class [CTCF-bound, Su(Hw)-bound, or Chro+Pzg+BEAF-32-bound] were transfected in parallel with spacer and *gypsy* control reporters into S2 cells in duplicates in a 96-well plate using 60 ng of reporter plasmid per replicate and Effectene (QIAGEN) following the manufacturer’s instructions. S2 cells were originally purchased from the American Type Culture Collection (reference number CRL-1963) in 2006. After 48 hours, fluorescence was measured on a NovoCyte Flow Cytometer (ACEA) using fluorescein isothiocyanate and phycoerythrin–Texas Red detection settings. Distributions of mCherry/EGFP fluorescence ratios in thousands of single transfected cells were plotted, and the median mCherry/EGFP ratio was extracted for each experiment. The average mCherry/EGFP log_2_ ratio obtained with the neutral spacer control was subtracted from all mCherry/EGFP log_2_ ratios obtained. Then, the average mCherry/EGFP log_2_ ratio obtained with the *gypsy* controls was set to 100% insulator strength. Relative insulator strengths of the tested fragments are expressed as percentage of *gypsy* insulator strength in Fig. 4C.

### Copurification of insulator protein interactors from embryo nuclear extracts

Soluble nuclear protein extracts were prepared from WT (OregonR) 0- to 14-hour embryos. Thirty grams of embryos was dechorionated, taken up in 30 ml of NU1 buffer [15 mM Hepes (pH 7.6), 10 mM KCl, 5 mM MgCl_2_, 0.1 mM EDTA (pH 8), 0.5 mM EGTA (pH 8), 350 mM sucrose, 2 mM dithiothreitol (DTT), and 0.2 mM phenylmethylsulfonyl fluoride], and Dounce-homogenized. The lysate was filtered through a double layer of Miracloth and then centrifuged for 15 min at 9000 rpm at 4°C. The nuclei pellet was resuspended and lysed in 30 ml of high-salt buffer [15 mM Hepes (pH 7.9), 400 mM KCl, 1.5 mM MgCl_2_, 0.2 mM EDTA, 20% glycerol, 1 mM DTT, and protease inhibitor cocktail] rotating for 20 min at 4°C and ultracentrifuged for 1 hour with an SW 40 rotor at 38,000 rpm at 4°C. The lipid layer was removed by suction, and the soluble nuclear extract was dialyzed into 15 mM Hepes (pH 7.9), 200 mM KCl, 1.5 mM MgCl_2_, 0.2 mM EDTA (pH 7.9), 20% glycerol, and 1 mM DTT with a 6- to 8-kDa molecular weight cutoff membrane. Soluble nuclear extract was snap-frozen in liquid nitrogen and stored at −80°C.

Baits with an N-terminal GFP-3C tag and a 3C-His_6_ C-terminal tag were purified from bacterial lysates by Ni–nitrilotriacetic acid affinity. Baits were full length when soluble or spanned soluble portions of baits when the full-length protein was not soluble. The following baits were used: Su(Hw)[1-219] (N terminus), CTCF[1-293] [N terminus; results reproduced from Kaushal *et al.* (*7*)], Cp190[1-1096] (full length), Chro[613-926] (C terminus), or *Xenopus* Nse I (full length) as an unrelated bait for negative control. Numbering is based on UniProt accession numbers P08970 [Su(Hw)], Q9VS55 (CTCF), Q24478 (Cp190), and Q86BS3 (Chro). Purified GFP-3C-bait-3C-His_6_ was immobilized on GFP binder beads, of which 30-μl bead volume was then incubated with 6 mg of *Drosophila* embryo nuclear extract in a total volume of 10 ml of IP buffer [50 mM tris-Cl (pH 7.5), 150 mM potassium acetate, 2 mM MgCl_2_, 10% glycerol, 0.1 mM DTT, 0.2% IGEPAL, and 1× cOmplete protease inhibitor cocktail] rotating for 3 hours at 4°C. Beads were washed three times with IP buffer, rotating for 10 min at 4°C for each wash. Proteins were eluted with 3C protease, adjusted to 1× SDS loading buffer, and loaded on an SDS–polyacrylamide gel electrophoresis (SDS-PAGE) gel. A duplicate experiment was similarly performed with nuclear protein extracts prepared from another biological replicate embryo sample.

Although CTCF and Su(Hw) pull-downs were only performed with N-terminal portions, peptides spanning full-length CTCF and Su(Hw) were respectively recovered, indicating that interactors of full-length proteins could be recovered. Pull-downs that we also performed with CTCF and Su(Hw) C-terminal portions did not recover additional interactors.

### Mass spectrometry analysis

For CTCF [previously described in (*7*)], Cp190, Chro, and their respective Nse I negative control pull-downs, protein samples were separated by SDS-PAGE and stained by Coomassie. Gel lanes between 10 and 300 kDa were excised into 5 to 10 pieces and digested with sequencing-grade trypsin. Extracted tryptic peptides were dried and resuspended in 0.05% trifluoroacetic acid (TFA) and 2% (v/v) acetonitrile. Tryptic peptide mixtures were injected on a Dionex RSLC 3000 nanoHPLC system (Dionex, Sunnyvale, CA, USA) interfaced via a nanospray source to a high-resolution mass spectrometer LTQ-Orbitrap Velos Pro (Thermo Fisher Scientific, Bremen, Germany) or timsTOF Pro (Bruker, Bremen, Germany). Peptides were loaded onto a trapping microcolumn Acclaim PepMap 100 C18 [20 mm by 100 μm; inside diameter (ID), 5 μm; Dionex] before separation on a C18 reversed-phase custom-packed column (75 μm ID by 40 cm; 1.8-μm particles; ReproSil-Pur, Dr. Maisch) using a gradient from 4 to 76% acetonitrile in 0.1% formic acid.

In the LTQ-Orbitrap Velos instrument, the 10 most intense multiply charged precursor ions detected with a full mass spectrometry (MS) survey scan in the Orbitrap [resolution of 60,000 at mass/charge ratio (*m*/*z*) of 400] were selected for collision-induced dissociation (normalized collision energy, 35%) and analysis in the ion trap. The window for precursor isolation was of 4.0 *m*/*z* units around the precursor, and selected fragments were excluded for 60 s from further analysis.

In the timsTOF instrument, data-dependent acquisition was carried out using a standard TIMS PASEF method with ion accumulation for 100 ms for both the survey MS1 scan and the TIMS-coupled MS2 scans. Duty cycle was kept at 100%. Up to 10 precursors were targeted per TIMS scan. Precursor isolation was done with 2 or 3 *m*/*z* windows below or above *m*/*z* of 800, respectively. The minimum threshold intensity for precursor selection was 2500. If the inclusion list allowed it, then precursors were targeted more than once to reach a minimum target total intensity of 20,000. Collision energy was ramped linearly uniquely on the basis of the 1/k0 values from 20 (at 1/k0 = 0.6) to 59 eV (at 1/k0 = 1.6). Total duration of a scan cycle including one survey and 10 MS2 TIMS scans was 1.16 s. Precursors could be targeted again in subsequent cycles if their signal increased by a factor 4.0 or more. After selection in 1 cycle, precursors were excluded from further selection for 60 s. Mass resolution in all MS measurements was approximately 35,000.

For Su(Hw) and its respective Nse I negative control pull-down, samples were digested following a modified version of the iST method. After dilution 1:1 (v/v) with 1% sodium deoxycholate, 100 mM tris (pH 8.6), and 10 mM DTT buffer, reduced disulfides were alkylated by adding ¼ vol of 160 mM chloroacetamide (final, 32 mM) and incubating at 25°C for 45 min in the dark. Samples were adjusted to 3 mM EDTA and digested with 1 μg of trypsin/LysC mix (Promega, #V5073) under gentle shaking for 1 hour at 37°C, followed by a second 1-hour digestion with a second identical aliquot of proteases. To remove sodium deoxycholate, two sample volumes of isopropanol containing 1% TFA were added to the digests, and the samples were desalted on a cation exchange plate (Oasis MCX microelution plate; Waters Corp., Milford, MA, prod.#186001830BA) by centrifugation. After washing with isopropanol/1% TFA, peptides were eluted in 250 μl of 80% acetonitrile (MeCN), 19% water, and 1% (v/v) ammonia. Eluates after steric exclusion chromatography desalting were dried and resuspended in 2% MeCN and 0.1% TFA before injection on a timsTOF mass spectrometer using a 110-min gradient from 4 to 76% acetonitrile in 0.1% formic acid.

Data files were analyzed with MaxQuant 1.6.3.4 incorporating the Andromeda search engine for protein identification and quantification based on iBAQ intensities. The following variable modifications were specified: cysteine carbamidomethylation (fixed) and methionine oxidation and protein N-terminal acetylation (variable). The sequence databases used for searching were *D. melanogaster* and *Escherichia coli* (strain K12) reference proteomes based on the UniProt database (www.uniprot.org; versions of 24 August 2020, containing 22,039 and 4391 sequences, respectively) and a contaminant database containing the most usual environmental contaminants and the enzymes used for digestion (keratins, trypsin, etc.). Both peptide and protein identifications were filtered at 1% false discovery rate relative to hits against a decoy database built by reversing protein sequences. The MaxQuant output tables proteinGroups.txt were processed with Perseus software to remove proteins matched to the contaminants database and proteins identified only by modified peptides or reverse database hits. Next, the tables were filtered to retain only proteins identified by a minimum of two peptides; the iBAQ quantitative values were log_2_-transformed, and *E. coli* proteins were removed. Missing values were imputed with the lowest value measured. Results are represented in Fig. 4A as the log_2_ average fold enrichment of proteins in biological duplicate pull-down experiments relative to that of a negative control pull-down (with GFP-tagged *Xenopus* Nse I as unrelated bait) done in parallel. Enrichments of baits themselves are not plotted because baits were exogenously added in large excess relative to endogenous proteins present in the nuclear extracts.

### Recombinant protein pull-downs

Expression plasmids encoding untagged full-length Cp190[1-1096] and/or Cp60[1-440] (with kanamycin resistance) were cotransformed with a plasmid expressing GFP-tagged CTCF[1-293] (N terminus), CTCF[610-818] (C terminus), Su(Hw)[1-219] (N terminus), Su(Hw)[724-941] (C terminus), Ibf1[1-242] (full length), or Ibf2[1-195] (full length) (with ampicillin resistance) into the *E. coli* Rosetta strain. Numbering is based on UniProt accession numbers Q24478 (Cp190), Q7K180 (Cp60), Q9VS55 (CTCF), P08970 [Su(Hw)], Q9VHG5 (Ibf1), and Q9VHG6 (Ibf2). Colonies were inoculated in 10 ml of TB cultures and grown at 37°C to an OD_600_ (optical density at 600 nm) of 1. The culture temperature was then reduced to 18°C, and 0.5 mM isopropyl-β-D-thiogalactopyranoside was added to induce protein expression. Cells were harvested after overnight incubation at 18°C, and the pellets were resuspended in 2 volumes of lysis buffer [50 mM tris (pH 7.5), 200 mM NaCl, 5% glycerol, and 25 mM imidazole]. Cells were lysed by sonication, and the lysate was clarified by centrifugation at 16,000*g* for 10 min at 4°C. The lysates were incubated for 1 hour at 4°C with 20 μl of GFP-binder resin. The beads were washed three times with 1 ml of lysis buffer and then boiled in SDS loading buffer to elute purified proteins, which were then visualized by SDS-PAGE and Coomassie staining.

### Annotation of embryonic enhancers at *Scr-ftz-Antp* locus

Figure 5B shows stripe enhancers active in early embryos. Enhancer 1 is VT37564 active in a stripe posterior to the cephalic furrow, thus overlapping *Scr* expression (*53*). Enhancer 2 is VT37565 active in *ftz* stripes 3 and 6 (*53*). Enhancer 3 is the *ftz* upstream element active in all seven *ftz* stripes (*56*). Enhancer 4 is the *ftz* zebra enhancer active in all seven stripes (*79*). Enhancer 5 is ftzDE active in *ftz* stripes 1 and 5 (*55*).

Figures S5B and 6B show selected enhancers active in later embryos and relevant to the discussed phenotypes. Enhancer 6 is VT37574 active in an anterior segment overlapping *Scr* expression (*53*). VT37574 overlaps the 3.7-kb Hind III and 7-kb Eco RI fragments (*52*) and also the T1 enhancer (*80*) described to be active in labial or prothoracic segments overlapping *Scr* expression. Enhancer 7 is the 6.8-kb Xba I fragment driving expression in hindgut and anal plate (*52*). This enhancer overlaps two inactive enhancers [VT37547 (*53*) and the 6.7-kb Bam HI fragment (*52*)] such that the probable active enhancer is entirely separated from the *Scr* promoter by an annotated Cp190 peak in WT (see Fig. 6B).

### Statistics and reproducibility

All described replicate experiments are biological (not technical) replicates. For all box plots, the center line denotes the median, box limits are upper and lower quartiles, the upper whisker extends to the largest value no further than 1.5× interquartile range from the upper hinge, the lower whisker extends to the smallest value no further than 1.5× interquartile range from the lower hinge, and points indicate outliers. Contingency tables in fig. S2 are colored by log_10_(*n*_observed_/*n*_expected_), where *n*_expected_ is the expected value assuming independence of rows and columns. This value was obtained for each cell as (row sum) × (column sum)/(table sum).

Animals were not separated by sex. Samples were grouped according to genotype (WT or various mutants). The investigators were not blinded during data collection, as the biological groups (genotypes) were well defined and handled in parallel. Computational analysis was performed by data scientists different from the researchers who collected the data. No data were excluded from the analyses.

For *Drosophila* viability tests, at least 100 animals were analyzed per genotype because clear differences between genotypes were visible already at this scale. For RNA-FISH experiments, approximately 20 embryos were examined per genotype over two independent experiments and only representative phenotypes that were observed in all animals are shown. These numbers were chosen because they revealed that phenotypes were reproducibly detected in all animals and because sample collection beyond this scale was rate limiting. For ChIP-seq, Hi-C, and Capture-seq experiments, at least 100 embryos were collected or 30 third instar larval brains were dissected per replicate because these numbers allowed sufficient material to be amplified for NG sequencing library preparation with a limited number of PCR cycles to avoid overamplification. This number was sufficient because all biological replicates were well correlated.

## Notes

View/request a protocol for this paper from *Bio-protocol*.
