# Supplementary material for: Essential role of Cp190 in physical and regulatory boundary formation
Source: Sci Adv. 2022 May 13;8(19):eabl8834. doi: 10.1126/sciadv.abl8834 (PMC9106302; doi:10.1126/sciadv.abl8834)
Supplement: Supplementary file 1 — Figs. S1 to S8 Tables S1 to S4 [file sciadv.abl8834_sm.pdf]

Science Advances
AAAS

Supplementary Materials for

Essential role of Cp190 in physical and regulatory boundary formation

Anjali Kaushal, Julien Dorier, Bihan Wang, Giriram Mohana, Michael Taschner, Pascal Cousin, Patrice Waridel, Christian Iseli, Anastasiia Semenova, Simon Restrepo, Nicolas Guex, Erez Lieberman Aiden, Maria Cristina Gambetta*

*Corresponding author. Email: mariacristina.gambetta@unil.ch

Published 13 May 2022, *Sci. Adv.* **8**, eabl8834 (2022)
DOI: 10.1126/sciadv.abl8834

The PDF file includes:

Figs. S1 to S8
Tables S1 to S4
Legends for data S1 to S15

Other Supplementary Material for this manuscript includes the following:

Data S1 to S15

Figure S1

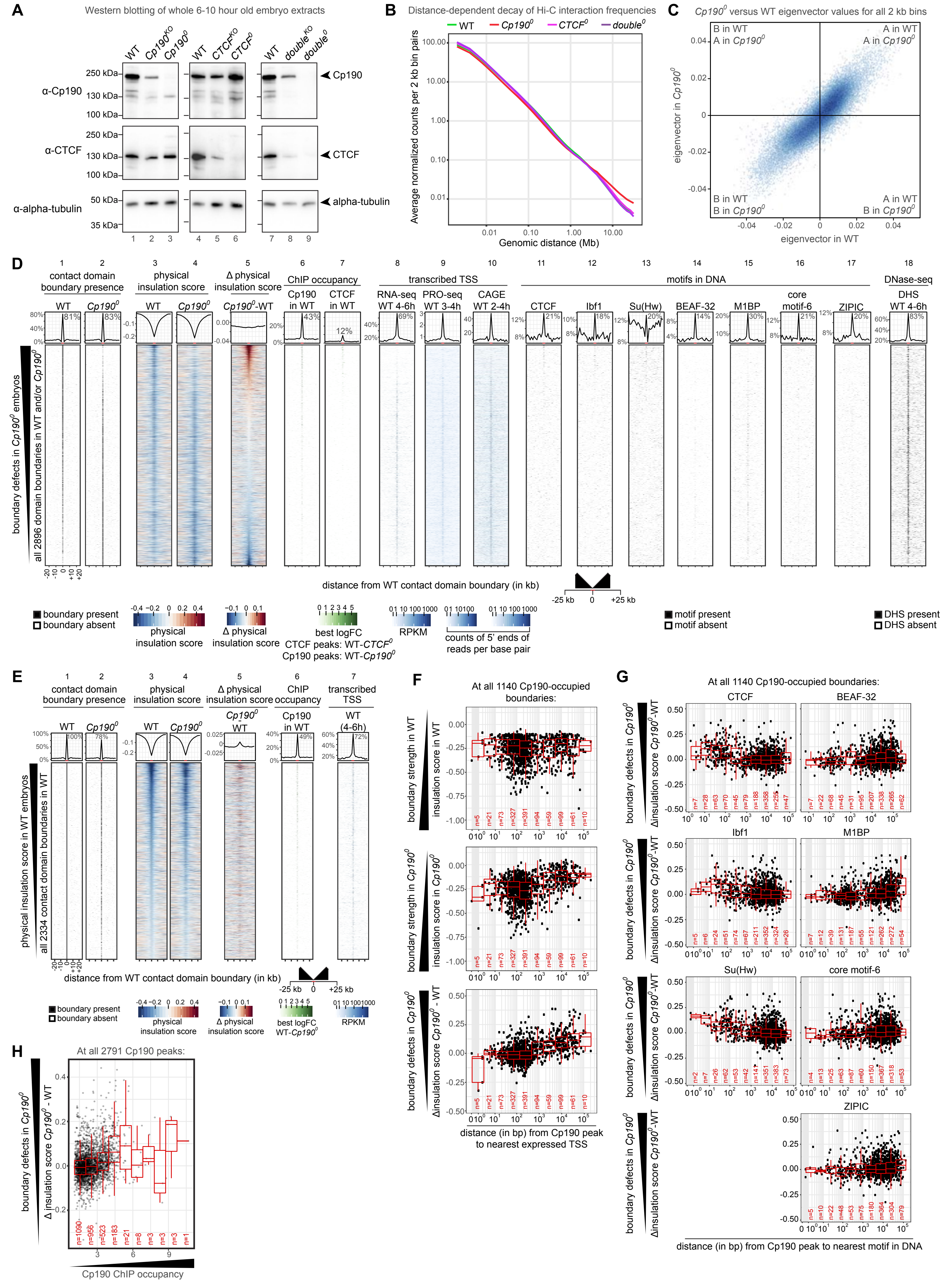
A
Western blotting of whole 6-10 hour old embryo extracts
WT Cp190KO Cp190⁰ WT CTCFKO CTCF⁰ WT doubleKO double⁰
α-Cp190
α-CTCF
α-alpha-tubulin
250 kDa
130 kDa
100 kDa
50 kDa
35 kDa
Cp190
CTCF
alpha-tubulin
1 2 3 4 5 6 7 8 9
B
Distance-dependent decay of Hi-C interaction frequencies
WT Cp190⁰ CTCF⁰ double⁰
Average normalized counts per 2 kb bin pairs
Genomic distance (Mb)
C
Cp190⁰ versus WT eigenvector values for all 2 kb bins
B in WT A in Cp190⁰
A in WT A in Cp190⁰
B in WT B in Cp190⁰
A in WT B in Cp190⁰
eigenvector in Cp190⁰
eigenvector in WT
D
contact domain boundary presence
physical insulation score
Δ physical insulation score
ChIP occupancy
transcribed TSS
motifs in DNA
DNase-seq
WT Cp190⁰ Cp190⁰-WT
Cp190 in WT
CTCF in WT
RNA-seq WT 4-6h
PRO-seq WT 3-4h
CAGE WT 2-4h
CTCF Ibf1 Su(Hw) BEAF-32 M1BP core motif-6 ZIPIC
DHS WT 4-6h
all 2896 domain boundaries in WT and/or Cp190⁰
boundary defects in Cp190⁰ embryos
distance from WT contact domain boundary (in kb)
-25 kb 0 +25 kb
boundary present
boundary absent
physical insulation score
Δ physical insulation score
best logFC
CTCF peaks: WT-CTCF⁰
Cp190 peaks: WT-Cp190⁰
RPKM
counts of 5' ends of reads per base pair
motif present
motif absent
DHS present
DHS absent
E
contact domain boundary presence
physical insulation score
Δ physical insulation score
ChIP occupancy
transcribed TSS
WT Cp190⁰
Cp190⁰ WT
Cp190 in WT
WT (4-6h)
all 2334 contact domain boundaries in WT
physical insulation score in WT embryos
distance from WT contact domain boundary (in kb)
-25 kb 0 +25 kb
boundary present
boundary absent
physical insulation score
Δ physical insulation score
best logFC WT-Cp190⁰
RPKM
F
At all 1140 Cp190-occupied boundaries:
boundary strength in WT
insulation score in WT
boundary strength in Cp190⁰
insulation score in Cp190⁰
boundary defects in Cp190⁰ - WT
Δinsulation score Cp190⁰ - WT
distance (in bp) from Cp190 peak to nearest expressed TSS
G
At all 1140 Cp190-occupied boundaries:
CTCF BEAF-32 Ibf1 M1BP Su(Hw) core motif-6 ZIPIC
boundary defects in Cp190⁰
Δinsulation score Cp190⁰-WT
distance (in bp) from Cp190 peak to nearest motif in DNA
H
At all 2791 Cp190 peaks:
boundary defects in Cp190⁰
Δ insulation score Cp190⁰ - WT
Cp190 ChIP occupancy

Fig. S1. Cp190 is required to form non-promoter boundaries in fly embryos. Related to Figure 1.

A. Western blotting of whole cell extracts from 6-10 hour old embryos of indicated genotypes. Blotting membranes were cut at the 70 kDa marker height. The upper part was probed first with anti-CTCF and then with anti-Cp190, while the lower part was probed with anti-alpha-tubulin to verify similar loading of each extract. Each picture delimited by a black border is of the same membrane. No Cp190 and/or CTCF signal is detected in $Cp190^0$ (lane 3), $CTCF^0$ (lane 6), and $double^0$ (lane 9) embryo extracts. The reduced Cp190 and/or CTCF signals in $Cp190^{KO}$ (lane 2), $CTCF^{KO}$ (lane 5), and $double^{KO}$ (lane 8) extracts represent maternally deposited proteins. The intense signals in wildtype extracts (lanes 1, 4, 7) represent summed maternally deposited and zygotically expressed CTCF and Cp190 proteins. CTCF levels seem normal in $Cp190^0$ (lane 3), and Cp190 levels seem normal in $CTCF^0$ (lane 6).

B. Average normalized Hi-C counts per pair of 2 kb bins (in y) as a function of genomic distance (using exponentially increasing bins in x) per genotype (merged biological quadruplicates downsampled to 79 million contacts shown for separately colored genotypes). This shows that all genotypes show comparable distance-dependent decays of Hi-C interaction frequencies up to over 1 Mb.

C. First eigenvector values in $Cp190^0$ (in y) versus WT (in x) for every 2 kb bin of chromosomes 2, 3 and X (density plot in blue, one point per 2 kb bin). Bins with positive eigenvector values are in the A (active) compartment, those with negative eigenvector values are in the B (inactive) compartment. Bins in the top left and bottom right quadrants are considered to be located in opposite compartments in $Cp190^0$ mutants relative to WT. This shows that compartments are not strongly affected in $Cp190^0$ mutant embryos.

D. Like top of Fig. 1B, but for all 2896 contact domain boundaries identified in 2-6 hour old WT and/or $Cp190^0$ embryos ranked by strongest (top) to weakest (bottom) physical insulation defects in $Cp190^0$ relative to WT. (1-2) Presence of boundaries called in each genotype by TopDom in 2 kb bins around the boundary center. (3-4) Physical insulation scores measured in each genotype. (5) Physical insulation score differences measured in $Cp190^0$ minus WT by Hi-C. (6-7) Cp190 and CTCF ChIP occupancy in WT. (8-10) Expressed transcription start sites (TSSs) detected by RNA-seq in WT 4-6 hour embryos [Graveley et al. (*75*)], by PRO-seq in WT 3-4 hour embryos or by CAGE in 2-4 hour embryos [Mikhaylichenko et al. (*76*)]. (11-17) Indicated motifs in DNA. (18) DNase-hypersensitive sites (DHSs) mapped by DNA-seq in WT 4-6 hour embryos [Reddington et al. (*78*)]. Summarized values (average physical insulation score/counts of 5' ends measured by PRO-seq or CAGE, or percentage of WT boundaries with boundary/ChIP peak/transcribed TSS/DNA motif/DHS present) across 2 kb bins are plotted on top, and enrichment ±2 kb around the central boundary (in red on x axis) is indicated. Color-coded value ranges are shown at the bottom. This shows that Cp190-dependent boundaries are generally TSS-distal, depleted for motifs enriched in promoter boundaries (BEAF-32,

M1BP, core motif-6, ZIPIC), and instead enriched in motifs found in non-promoter boundaries (CTCF, Ibf1, Su(Hw)). In contrast, boundaries only found in *Cp190⁰* but absent in WT are not visibly associated with any of the analyzed features in WT embryos, and their origins remain unclear.

E. Like top of Fig. 1B, but all 2334 WT contact domain boundaries are ranked by strongest (top) to weakest (bottom) physical insulation scores measured in WT. (1-2) Presence of boundaries called in each genotype by TopDom in 2 kb bins around the boundary center. (3-4) Physical insulation scores measured in each genotype. (5) Physical insulation score differences measured in *Cp190⁰* minus WT by Hi-C, showing that Cp190 loss affects boundaries of variable strengths. (6) Cp190 ChIP occupancy in WT. (7) Expressed transcription start sites (TSSs) in WT 4-6 hour embryos [Graveley et al. (*75*)].

F. Scatter plot of physical insulation scores measured by Hi-C in WT (top) or in *Cp190⁰* (middle) embryos, or of physical insulation score differences in *Cp190⁰* minus WT (bottom) (in y) at all 1140 Cp190-occupied boundaries (i.e. all boundaries within ±2 kb of a Cp190 ChIP peak in WT, points) versus distance (in bp, in $\log_{(x+1)}$ transformed x axis) from the position of the most boundary-proximal Cp190 peak to the nearest transcribed TSS (RPKM>0) in WT 4-6 hour old embryos [expression data from Graveley et al. (*75*)]. Box plots of indicated n Cp190 peaks binned by distance to expressed TSS are overlaid. Box plot center line is median; box limits are upper and lower quartiles; whiskers are 1.5x interquartile ranges. This shows that Cp190-occupied boundaries that are close to or far from expressed TSSs have similar strengths in WT, but boundaries far from expressed TSSs are more severely affected by Cp190 loss. These scatter plots accompany the box plots shown in Fig. 1C.

G. Scatter plot of physical insulation score differences measured in *Cp190⁰* minus WT Hi-C maps (in y) at all 1140 Cp190-occupied boundaries (points) versus distance (in bp, in $\log_{(x+1)}$ transformed x axis) from the position of the most boundary-proximal Cp190 peak to the center of the nearest indicated motif. Motifs enriched in non-promoter boundaries are in the first column; motifs enriched in promoter boundaries are in the second column. Box plots of indicated n Cp190 peaks binned by distance to motif are overlaid. This shows that insulation defects in *Cp190⁰* are higher closer to motifs enriched in non-promoter boundaries (CTCF, Ibf1, Su(Hw)) than to motifs enriched at promoter boundaries (BEAF-32, M1BP, core motif-6, ZIPIC). These scatter plots accompany the box plots shown in Fig. 1D.

H. Scatter plot of physical insulation score differences measured in *Cp190⁰* minus WT Hi-C maps (in y) versus Cp190 ChIP occupancy (in x) for each WT Cp190 ChIP peak (points). Insulation scores are measured at the nearest bin boundary to the Cp190 peak position. Box plots of indicated n Cp190 peaks binned by ChIP occupancy are overlaid. This shows that insulation defects in *Cp190⁰* scale with Cp190 ChIP occupancy, except at rare very high occupancy Cp190 ChIP peaks.

Figure S2

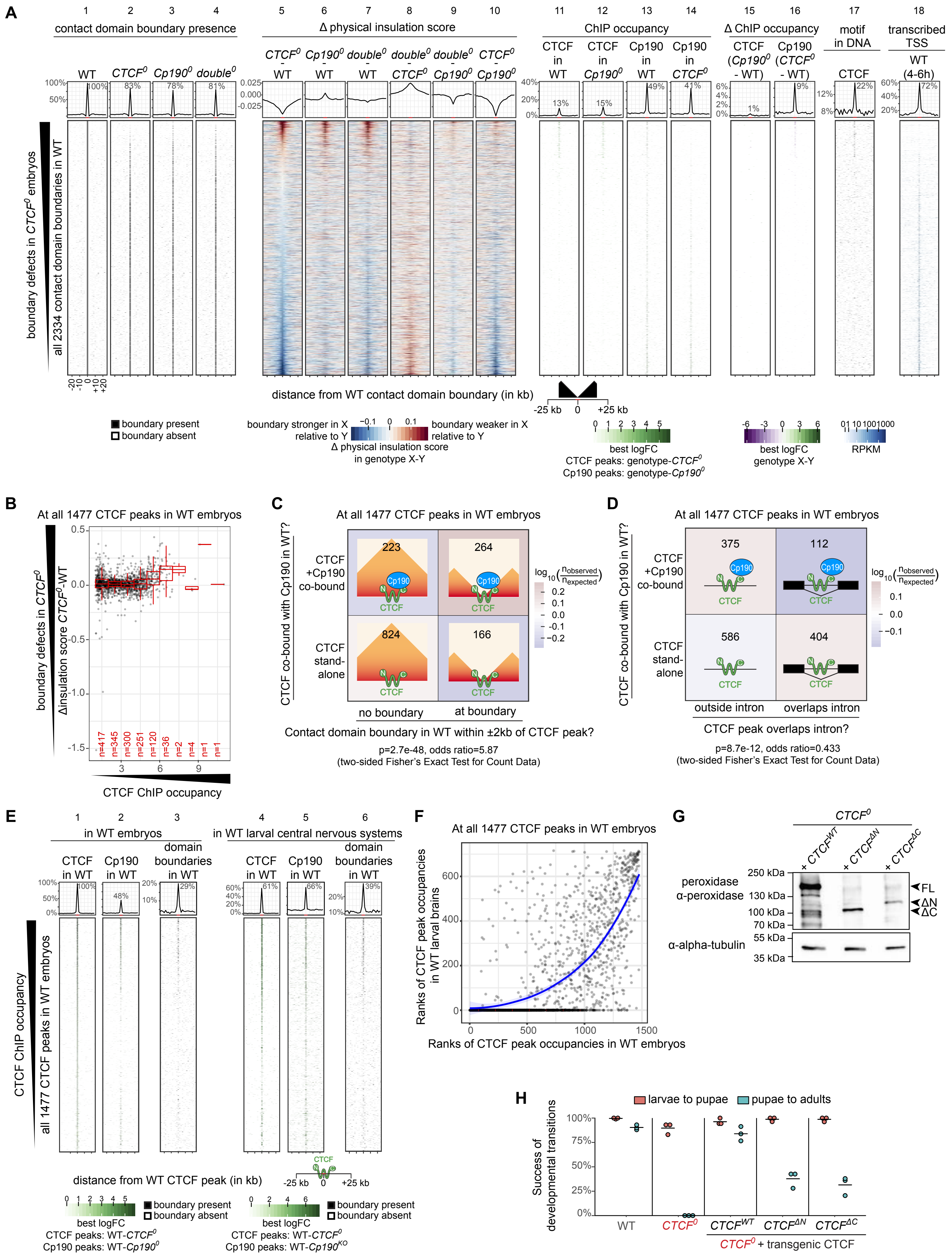
A
contact domain boundary presence
Δ physical insulation score
ChIP occupancy
Δ ChIP occupancy
motif in DNA
transcribed TSS
WT
CTCF0
Cp190 0
double 0
CTCF0 - WT
Cp190 0 - WT
double 0 - WT
double 0 - CTCF0
double 0 - Cp190 0
CTCF0 - Cp190 0
CTCF in WT
CTCF in Cp190 0
Cp190 in WT
Cp190 in CTCF0
CTCF (Cp190 0 - WT)
Cp190 (CTCF0 - WT)
CTCF
WT (4-6h)
boundary defects in CTCF0 embryos
all 2334 contact domain boundaries in WT
distance from WT contact domain boundary (in kb)
-25 kb
0
+25 kb
boundary present
boundary absent
boundary stronger in X relative to Y
boundary weaker in X relative to Y
Δ physical insulation score in genotype X-Y
best logFC
CTCF peaks: genotype-CTCF0
Cp190 peaks: genotype-Cp190 0
best logFC genotype X-Y
RPKM
B
At all 1477 CTCF peaks in WT embryos
boundary defects in CTCF0
Δinsulation score CTCF0-WT
n=417
n=345
n=300
n=251
n=120
n=36
n=2
n=4
n=1
n=1
CTCF ChIP occupancy
C
At all 1477 CTCF peaks in WT embryos
CTCF co-bound with Cp190 in WT?
CTCF +Cp190 co-bound
CTCF stand-alone
223
264
824
166
no boundary
at boundary
Contact domain boundary in WT within ±2kb of CTCF peak?
p=2.7e-48, odds ratio=5.87
(two-sided Fisher's Exact Test for Count Data)
D
At all 1477 CTCF peaks in WT embryos
CTCF co-bound with Cp190 in WT?
CTCF +Cp190 co-bound
CTCF stand-alone
375
112
586
404
outside intron
overlaps intron
CTCF peak overlaps intron?
p=8.7e-12, odds ratio=0.433
(two-sided Fisher's Exact Test for Count Data)
E
in WT embryos
in WT larval central nervous systems
CTCF in WT
Cp190 in WT
domain boundaries in WT
CTCF ChIP occupancy
all 1477 CTCF peaks in WT embryos
distance from WT CTCF peak (in kb)
-25 kb
+25 kb
boundary present
boundary absent
best logFC
CTCF peaks: WT-CTCF0
Cp190 peaks: WT-Cp190 0
CTCF peaks: WT-CTCF0
Cp190 peaks: WT-Cp190 KO
F
At all 1477 CTCF peaks in WT embryos
Ranks of CTCF peak occupancies in WT larval brains
Ranks of CTCF peak occupancies in WT embryos
G
CTCF0
× CTCF WT
× CTCF ΔN
× CTCF ΔC
peroxidase α-peroxidase
α-alpha-tubulin
250 kDa
130 kDa
100 kDa
70 kDa
55 kDa
35 kDa
FL
ΔN
ΔC
H
larvae to pupae
pupae to adults
Success of developmental transitions
100%
75%
50%
25%
0%
WT
CTCF0
CTCF WT
CTCF ΔN
CTCF ΔC
CTCF0 + transgenic CTCF

Fig. S2. Cp190 is required for boundary formation at CTCF peaks. Related to Figure 2.

A. Distribution of indicated datasets in ±25 kb windows centered around all 2334 contact domain boundaries identified in WT embryos ranked by strongest (top) to weakest (bottom) boundary defects measured in $CTCF^0$ mutant embryos. (1-4) Presence of contact domain boundaries called in each genotype by TopDom in 2 kb bins around the boundary center. (5-10) Physical insulation score differences measured in genotype X (top) minus genotype Y (bottom) by Hi-C. (11-12) CTCF or (13-14) Cp190 ChIP occupancy in indicated genotypes. (15-16) Differential CTCF and Cp190 ChIP occupancy in genotype X (top) minus genotype Y (bottom). (17) CTCF motif presence in DNA. (18) Expressed TSSs in WT 4-6 hour old embryos [Graveley et al. (*75*)]. Summarized values (average physical insulation score or percentage of WT boundaries with boundary/ChIP peak/differentially bound region/motif/transcribed TSS present) across 2 kb bins are shown above with indicated enrichments ±2 kb around the central boundary. Color-coded value ranges are shown at the bottom. This shows that CTCF is present ±2 kb from 13% of all WT boundaries (lane 11), and that boundary defects in $CTCF^0$ mutants (lanes 2, 5) are specifically observed at boundaries occupied by CTCF in WT (lane 11).

B. Scatter plot of physical insulation score differences measured in $CTCF^0$ minus WT Hi-C maps (in y) versus CTCF ChIP occupancy (in x) for each CTCF peak (points). Insulation scores are measured at the nearest bin boundary to the CTCF peak position. Box plots of indicated n CTCF peaks binned by ChIP occupancy are overlaid. Box plot center line is median; box limits are upper and lower quartiles; whiskers are 1.5x interquartile ranges. This shows that insulation defects in $CTCF^0$ are observed at low and high occupancy CTCF ChIP peaks, but not at intermediate occupancy CTCF ChIP peaks (which are not at boundaries – see Fig. 2A).

C. Numbers of CTCF peaks in WT (n=1477 peaks) that overlap a Cp190 peak in WT or not (rows), and have a contact domain boundary in WT within ±2 kb of the peak position or not (columns). Cells are colored by $\log_{10}(_{observed}/n_{expected})$, where $n_{expected}$ is the expected value assuming independence of rows and columns (see Methods). CTCF+Cp190 co-localization is significantly positively associated with localization at a boundary (odds ratio and p-value are from two-sided Fisher's Exact Test for Count Data).

D. Numbers of CTCF peaks in WT (n=1477 peaks) that overlap a Cp190 peak in WT or not (rows), and whose peak position is inside an intron or not (columns). Cells are colored by $\log_{10}(n_{observed}/n_{expected})$, where $n_{expected}$ is the expected value assuming independence of rows and columns (see Methods). CTCF co-localization with Cp190 is significantly negatively associated with localization in an intron (odds ratio and p-value are from two-sided Fisher's Exact Test for Count Data).

E. Occupancy of CTCF ChIP peaks in WT embryos (this study) compared to occupancy of CTCF peaks previously mapped in WT larval central nervous systems [from Kaushal et al.

(*7*)]. All 1477 WT CTCF peaks in embryos are ranked from highest (top) to lowest (bottom) ChIP occupancy (lane 1). Intermediate occupancy CTCF peaks frequently do not colocalize with Cp190 (lane 2) or with contact domain boundaries in WT (lane 3) (these panels were reproduced from Fig. 2A). Intermediate CTCF peaks in WT embryos are often low or zero occupancy CTCF peaks in larval central nervous systems (lane 4), and also overlap less frequently a Cp190 peak (lane 5) and a contact domain boundary (lane 6) in WT larval central nervous systems.

F. Scatter plot of ranks of CTCF peak occupancies in WT larval central nervous systems (in y) versus ranks of CTCF peak occupancies in WT embryos (in x). Ranks are assigned by ordering non-zero peak occupancies by increasing order and assigning increasing ranks from 1 to the number of non-zero peak occupancies (see Methods). Ties are replaced by average rank, while rank 0 is assigned to unoccupied peaks. Each point is a CTCF peak in WT embryos, with CTCF peaks in WT larval central nervous systems [data from Kaushal et al. (*7*)] set to rank 0 when no CTCF peaks in WT larval central nervous systems overlap the corresponding CTCF peak in WT embryos. Loess fit (blue line) with 95% confidence interval (blue shade) is added. This illustrates that intermediate occupancy CTCF peaks in WT embryos are generally low occupancy CTCF peaks in larval central nervous systems.

G. Western blotting of whole-cell extracts prepared from dissected larval central nervous systems of $CTCF^0$ animals rescued with transgenes expressing wildtype CTCF ($CTCF^{WT}$) or CTCF completely lacking N- ($CTCF^{\Delta N}$) or C- ($CTCF^{\Delta C}$) termini, under the control of *CTCF* regulatory sequences. $CTCF^{\Delta N}$ lacks amino acids 1-293, $CTCF^{\Delta C}$ lacks amino acids 610-818 (numbering according to Uniprot accession Q9VS55). $CTCF^{WT}$ and $CTCF^{\Delta N}$ were expressed as C-terminal tandem affinity purification (TAP) tag fusions, $CTCF^{\Delta C}$ was expressed as an N-terminal TAP tag fusion. Extracts were probed with peroxidase anti-peroxidase antibody complex that binds to the TAP tag (top), then with anti-alpha-tubulin to verify equal loading of each extract. Observed sizes of CTCF versions are marked by arrowheads on the right.

H. Percentages (in y) of wildtype (WT), $CTCF^0$, and $CTCF^0$ animals rescued with $CTCF^{WT}$, $CTCF^{\Delta N}$, or $CTCF^{\Delta C}$ transgenically expressed proteins that successfully transitioned from third instar larva to pupa (pink) and from pupa to adult (blue) in 3 biological replicates (each starting with between 83 and 100 larvae). Horizontal lines show means.

Figure S3

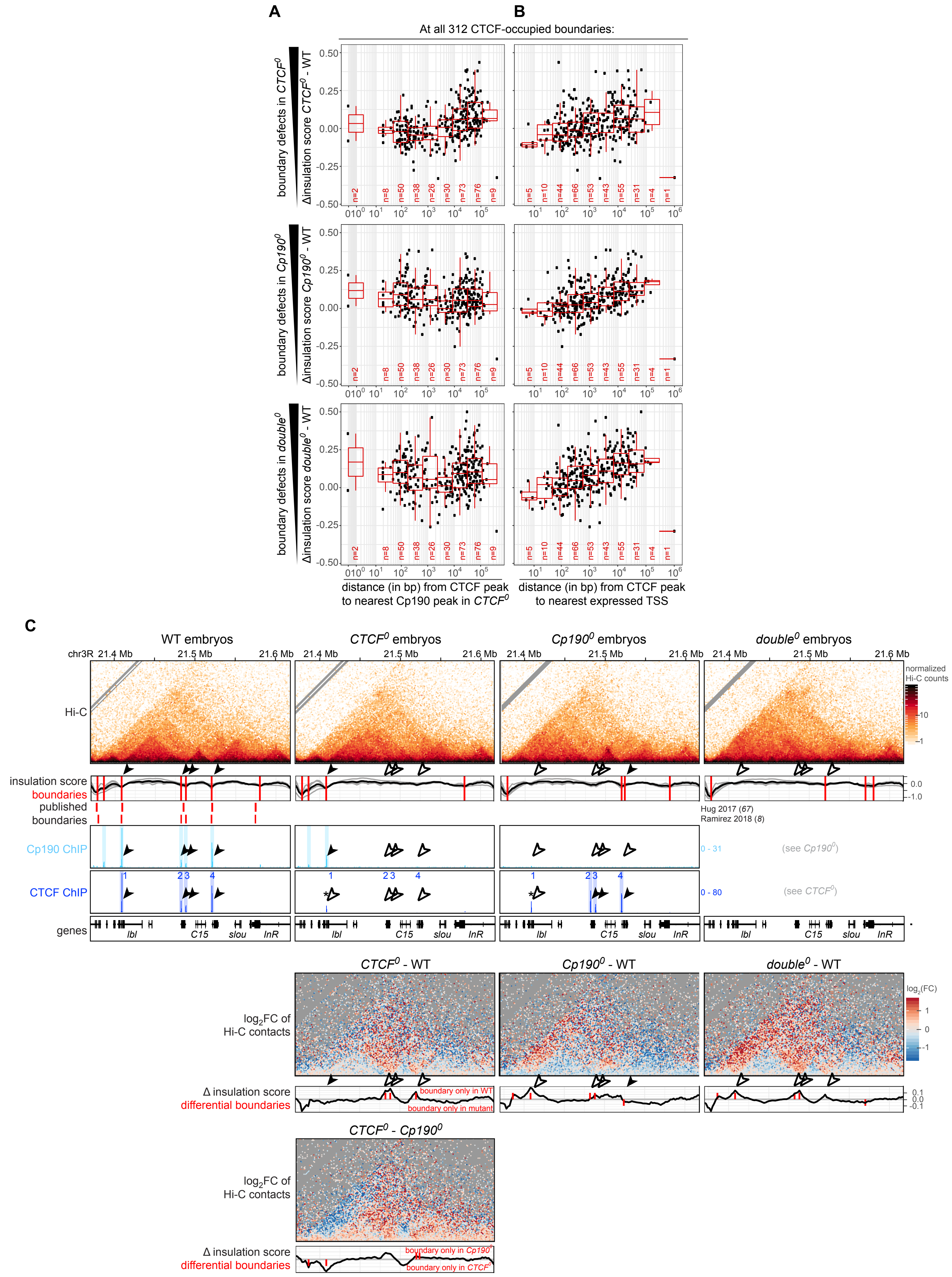

A
B
At all 312 CTCF-occupied boundaries:
boundary defects in CTCF0
Δinsulation score CTCF0 - WT
boundary defects in Cp1900
Δinsulation score Cp1900 - WT
boundary defects in double0
Δinsulation score double0 - WT
distance (in bp) from CTCF peak to nearest Cp190 peak in CTCF0
distance (in bp) from CTCF peak to nearest expressed TSS
C
WT embryos
CTCF0 embryos
Cp1900 embryos
double0 embryos
chr3R 21.4 Mb
21.5 Mb
21.6 Mb
Hi-C
normalized Hi-C counts
insulation score
boundaries
published boundaries
Hug 2017 (67)
Ramirez 2018 (8)
Cp190 ChIP
CTCF ChIP
(see Cp1900)
(see CTCF0)
genes
lbl
C15
slou
InR
CTCF0 - WT
Cp1900 - WT
double0 - WT
log2FC of Hi-C contacts
log2(FC)
Δ insulation score
differential boundaries
boundary only in WT
boundary only in mutant
CTCF0 - Cp1900
boundary only in Cp1900
boundary only in CTCF0

Fig. S3. CTCF-occupied boundaries are differentially sensitive to loss of CTCF or Cp190. Related to Figure 3.

A. Scatter plot of physical insulation score differences measured by Hi-C in *CTCF⁰* (top), *Cp190⁰* (middle) or *double⁰* (bottom) minus WT (in y) at all 312 boundaries occupied by CTCF in WT (i.e. all boundaries within ±2 kb of a CTCF ChIP peak in WT, points) versus distance (in bp, in $\log_{(x+1)}$ transformed x axis) from the position of the most boundary-proximal CTCF peak to the nearest Cp190 peak in *CTCF⁰*. Box plots of indicated n CTCF peaks binned by distance to the Cp190 peak are overlaid. Box plot center line is median; box limits are upper and lower quartiles; whiskers are 1.5x interquartile ranges. This shows that boundary defects in *CTCF⁰* are smaller when the boundary is close to a residual Cp190 peak, which is not the case in *Cp190⁰* or *double⁰*. These scatter plots accompany the box plots shown in Fig. 3C.

B. Same as A but versus distance from the position of the most boundary-proximal CTCF peak to the nearest transcribed TSS (RPKM>0) in WT 4-6 hour old embryos [expression data from Graveley et al. (*75*)]. This shows that insulation defects in all genotypes are smaller at boundaries close to transcribed TSSs. These scatter plots accompany the box plots shown in Fig. 3D.

C. Example locus (dm6 coordinates) Hi-C maps, physical insulation score (calculated with different window sizes in gray, average in black) and contact domain boundaries (vertical red lines) from this study (above) and published Hi-C studies in embryos [Hug et al. (*67*)] and tissue culture cells [Ramírez et al. (*8*)] (below), Cp190 ChIP-seq with Cp190 peaks defined in the respective genotype relative to *Cp190⁰* highlighted in light blue, CTCF ChIP-seq with CTCF peaks defined in the respective genotype relative to *CTCF⁰* highlighted in dark blue and numbered 1 to 4, and gene tracks (only longest isoform of each protein-coding gene shown) in embryos of the indicated genotypes. ChIP-seq scale is reads per million. Differential Hi-C maps (mutants minus WT in row 2, and *CTCF⁰* minus *Cp190⁰* in row 3), physical insulation score and contact domain boundaries are shown below. A non-specific CTCF ChIP-seq signal detected in *CTCF⁰* is marked by a black asterisk (the signal was higher in WT and thus called a CTCF peak, but it was not sufficiently high in *Cp190⁰* to be called a CTCF peak in the differential analysis). Arrowheads point to CTCF+Cp190 co-bound peaks located at domain boundaries in WT, while empty arrowheads indicate sites where these peaks are absent in the indicated genotypes. Cp190 co-localizing with CTCF peak 1 is partially CTCF-dependent because its peak height is reduced but the peak is still detected in *CTCF⁰*. Cp190 co-localizing with CTCF peaks 2-4 are strictly CTCF-dependent because these peaks are lost in *CTCF⁰*. This locus illustrates that contact domain boundaries at partially CTCF-dependent Cp190 peaks (peak 1) are more strongly affected in *Cp190⁰* than in *CTCF⁰*, whereas the strictly CTCF-dependent Cp190 peaks are more strongly affected in *CTCF⁰* than in *Cp190⁰*.

Figure S4

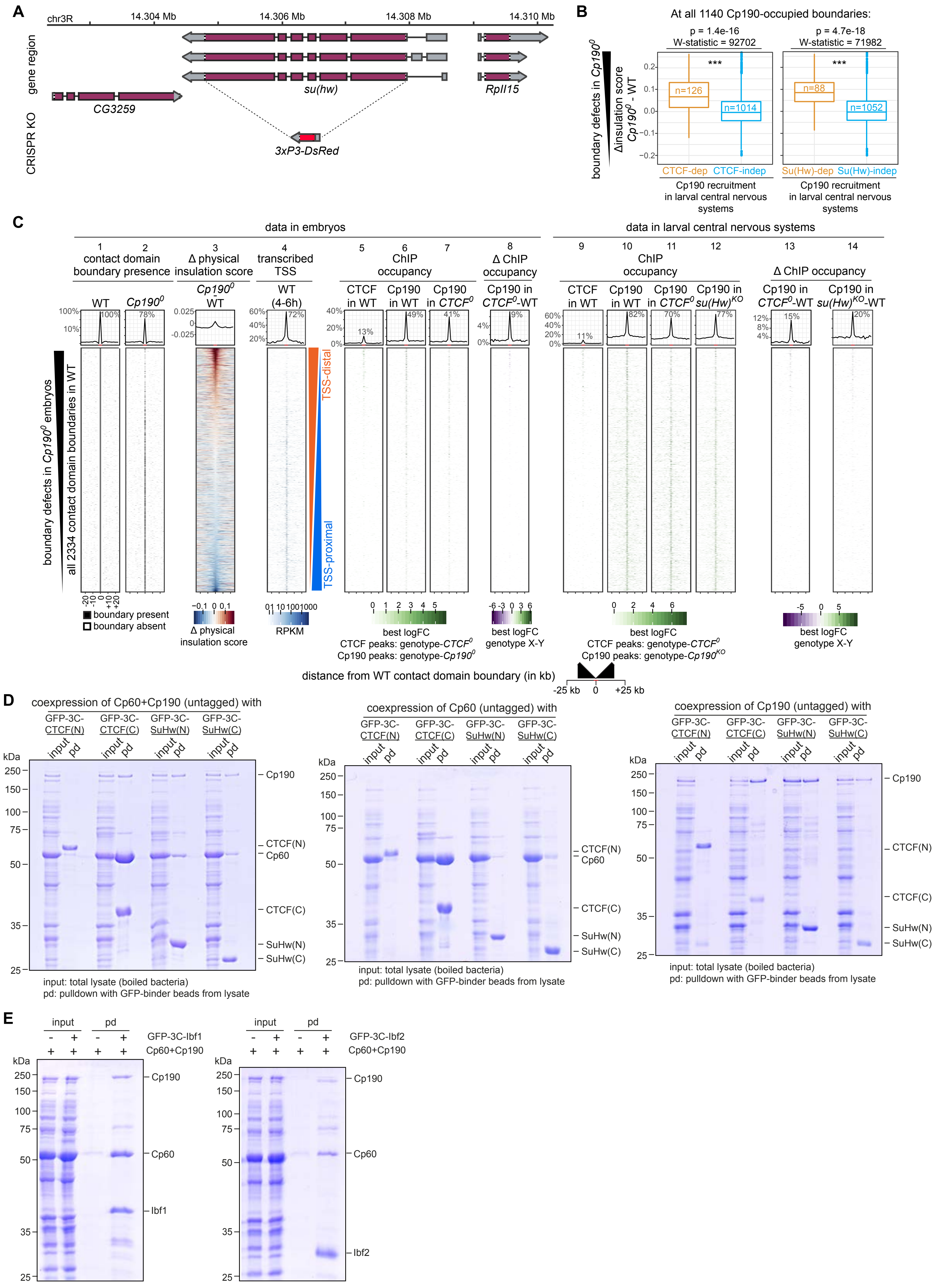
A
chr3R
14.304 Mb
14.306 Mb
14.308 Mb
14.310 Mb
gene region
CRISPR KO
CG3259
su(hw)
Rpl15
3xP3-DsRed
B
At all 1140 Cp190-occupied boundaries:
p = 1.4e-16
W-statistic = 92702
p = 4.7e-18
W-statistic = 71982
boundary defects in Cp190⁰
Δinsulation score Cp190⁰ - WT
n=126
n=1014
n=88
n=1052
CTCF-dep
CTCF-indep
Su(Hw)-dep
Su(Hw)-indep
Cp190 recruitment in larval central nervous systems
C
data in embryos
data in larval central nervous systems
contact domain boundary presence
Δ physical insulation score
transcribed TSS
ChIP occupancy
Δ ChIP occupancy
WT
Cp190⁰
Cp190⁰ - WT
WT (4-6h)
CTCF in WT
Cp190 in WT
Cp190 in CTCF⁰
Cp190 in CTCF⁰-WT
Cp190 in su(Hw)KO
Cp190 in su(Hw)KO-WT
boundary defects in Cp190⁰ embryos
all 2334 contact domain boundaries in WT
TSS-distal
TSS-proximal
boundary present
boundary absent
Δ physical insulation score
RPKM
best logFC
CTCF peaks: genotype-CTCF⁰
Cp190 peaks: genotype-Cp190⁰
best logFC genotype X-Y
Cp190 peaks: genotype-Cp190KO
distance from WT contact domain boundary (in kb)
-25 kb
0
+25 kb
D
coexpression of Cp60+Cp190 (untagged) with
coexpression of Cp60 (untagged) with
coexpression of Cp190 (untagged) with
GFP-3C-CTCF(N)
GFP-3C-CTCF(C)
GFP-3C-SuHw(N)
GFP-3C-SuHw(C)
input
pd
kDa
Cp190
CTCF(N)
Cp60
CTCF(C)
SuHw(N)
SuHw(C)
input: total lysate (boiled bacteria)
pd: pulldown with GFP-binder beads from lysate
E
GFP-3C-Ibf1
Cp60+Cp190
GFP-3C-Ibf2
Ibf1
Ibf2

Fig. S4. Cp190 assembly into diverse multisubunit complexes. Related to Figure 4.

A. *su(Hw)* extended gene region with coding (purple) and noncoding (grey) exons and introns (lines). In *su(Hw)*^{KO} mutants, a *DsRed* selection marker replaces the *su(Hw)* open reading frame.

B. Boxplots of physical insulation score differences in *Cp190*^{0} minus WT embryos at all 1140 contact domain boundaries occupied by Cp190 in WT (i.e. all boundaries within ±2 kb of a Cp190 ChIP peak in WT) at which the Cp190 peak in embryos overlaps a CTCF-dependent Cp190 peak in WT larval central nervous systems [left, data from Kaushal et al. (*7*)] or a Su(Hw)-dependent Cp190 peak in WT larval central nervous systems (right). (Note that Su(Hw)-dependent Cp190 peaks had to be mapped in larvae instead of embryos because *su(Hw)*^{0} mutants cannot be generated.) Indicated p value and W-statistic from two-sided Wilcoxon rank-sum test with continuity correction. Box plot center line is median; box limits are upper and lower quartiles; whiskers are 1.5× interquartile ranges; points are outliers; n = Cp190-occupied boundaries of indicated categories.

C. Distribution of indicated datasets in ±25 kb windows centered around all 2334 contact domain boundaries identified in WT ranked by strongest (top) to weakest (bottom) physical insulation defects in *Cp190*^{0} relative to WT. (1-2) Presence of contact domain boundaries called in each genotype by TopDom in 2 kb bins around the boundary center. (3) Physical insulation score differences measured in *Cp190*^{0} minus WT by Hi-C. (4) Expressed TSSs in WT 4-6 hour old embryos [expression data from Graveley et al. (*75*)]. (5-7) CTCF and Cp190 ChIP occupancy in embryos of indicated genotypes. (8) Differential Cp190 ChIP occupancy in *CTCF*^{0} minus WT embryos. (9-12) CTCF and Cp190 ChIP occupancy in WT, *CTCF*^{0} or *su(Hw)*^{KO} larval central nervous systems [data in lanes 9 and 11 is from Kaushal et al. (*7*)]. (13-14) Differential Cp190 ChIP occupancy in *CTCF*^{0} or *su(Hw)*^{KO} mutants minus WT larval central nervous systems. Summarized values (average physical insulation score or percentage of WT boundaries with boundary/ChIP peak/differentially bound region/transcribed TSS present) across 2 kb bins are shown above with indicated enrichments ±2 kb around the central boundary (highlighted in red on x axis). Color-coded value ranges are shown at the bottom. Lanes 1-4 and 6 were reproduced from Fig. 1B. This shows that Cp190 is recruited to Cp190-dependent boundaries by CTCF or Su(Hw).

D. GFP pull-down of tagged CTCF N-terminus (residues 1-123), CTCF C-terminus (residues 610-818), Su(Hw) N-terminus (residues 1-219), or Su(Hw) C-terminus (residues 724-941), each co-expressed with untagged Cp190 and Cp60 (left), Cp60 alone (middle) or Cp190 alone (right). Positions of molecular weight ladder bands are marked on the left, those of co-expressed proteins are marked on the right. Amino acid numbering is based on Uniprot entries Q9VS55 (CTCF) and P08970 (Su(Hw)).

E. GFP pull-down of tagged full-length Ibf1 (left) or Ibf2 (right), each co-expressed with untagged Cp190 and Cp60. GFP pull-down in the absence of Ibf1 or Ibf2 is shown as negative control. Positions of molecular weight ladder bands are marked on the left, those of co-expressed proteins are marked on the right.

Figure S5

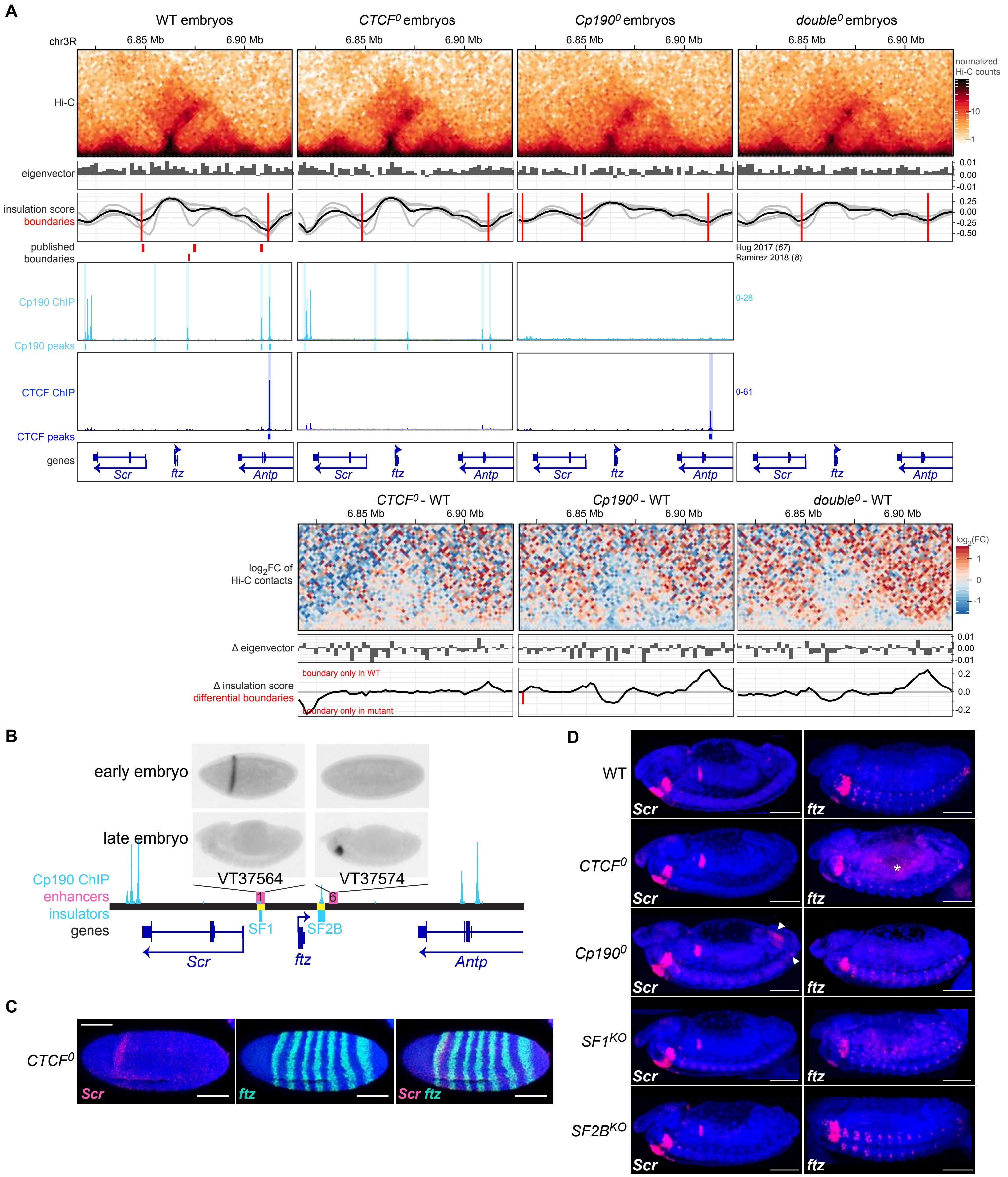

A
WT embryos
CTCF0 embryos
Cp1900 embryos
double0 embryos
chr3R
6.85 Mb
6.90 Mb
Hi-C
normalized Hi-C counts
eigenvector
insulation score
boundaries
published boundaries
Hug 2017 (67)
Ramirez 2018 (8)
Cp190 ChIP
Cp190 peaks
0-28
CTCF ChIP
CTCF peaks
0-61
genes
Scr
ftz
Antp
CTCF0 - WT
Cp1900 - WT
double0 - WT
log2FC of Hi-C contacts
log2(FC)
Δ eigenvector
Δ insulation score
differential boundaries
boundary only in WT
boundary only in mutant
B
early embryo
late embryo
VT37564
VT37574
Cp190 ChIP
enhancers
insulators
genes
SF1
SF2B
C
CTCF0
Scr
ftz
Scr ftz
D
WT
CTCF0
Cp1900
SF1KO
SF2BKO

Fig. S5. Cp190 prevents regulatory crosstalk between early patterning gene loci. Related to Figure 5.

A. Same locus shown in Fig. 5A additionally showing eigenvector values (2 kb resolution, positive for A compartment, negative for B compartment), and CTCF ChIP-seq with CTCF peaks defined in the respective genotype relative to $CTCF^0$ in dark blue, in embryos of the indicated genotypes. Differential Hi-C maps, physical insulation scores and contact domain boundaries in the respective mutants minus WT are shown below. Note limitations in TopDom boundary calls at this locus: the *ftz* upstream boundary was called but is shifted to the left of its visible position in the Hi-C map, and the *ftz* downstream boundary was not called robustly by TopDom and was hence subsequently filtered out in our analysis (see Methods). *ftz* boundaries are therefore visible by Hi-C but were not called because they challenge the classical definition of boundaries due to the unusually strong inter-domain interactions occurring across them at this exceptional locus.

B. Same locus shown in A. Enhancer 1 (also shown in Fig. 5B) is VT37564 [Kvon et al. (*53*)] and drives expression in early gastrulae overlapping *Scr* expression (i.e. it is expressed in a stripe just posterior to the cephalic furrow like *Scr*), as shown on top. Enhancer 1 is silent in older embryos (shown below), and $SF1^{KO}$ older embryos consistently express *Scr* normally [as shown in panel D row 1 and previously reported by Yokoshi et al. (*59*)]. Enhancer 6 is VT37574 [Kvon et al. (*53*)] and drives expression in the labial segment overlapping *Scr* expression (compare to *Scr* expression at the same embryonic stage shown in panel D row 1), consistent with the possibility that it may be a distal *Scr* enhancer in older embryos. Enhancer 6 is silent in young embryos, further suggesting that it is unlikely to be responsible for early *Scr* expression. Pictures of reporter gene expression driven by these enhancers were obtained from https://enhancers.starklab.org/.

C. Like Fig. 5C. RNA-FISH with co-hybridized antisense probes against *Scr* (red) and *ftz* (green) mRNAs in early gastrula $CTCF^0$ embryos stained with DAPI (blue) and imaged from the side (anterior left, posterior right, scale bars 100 µm). Single *Scr* and *ftz* images are shown on the left, and merged images of the same embryo is shown on the right. This shows that *Scr* and *ftz* appear normally expressed in $CTCF^0$ embryos.

D. RNA-FISH with antisense probes (red) against *Scr* (left) or *ftz* mRNAs (right) in stage 14 (mid-embryogenesis) embryos stained with DAPI (anterior left, posterior right, scale bars 100 µm). In WT embryos, *Scr* is expressed in anterior (labial and prothoracic) segments and the anterior midgut. *Scr* is expressed normally in all genotypes (rows) except $Cp190^0$ mutants which misexpress *Scr* in the hindgut and anal plate (arrowheads). Misexpression in anal plate becomes stronger in older embryos (see Fig. 6C). In WT embryos, *ftz* is expressed in a subset of cells of the ventral nerve cord. *ftz* is expressed normally in all genotypes (white asterisk marks background staining).

Figure S6

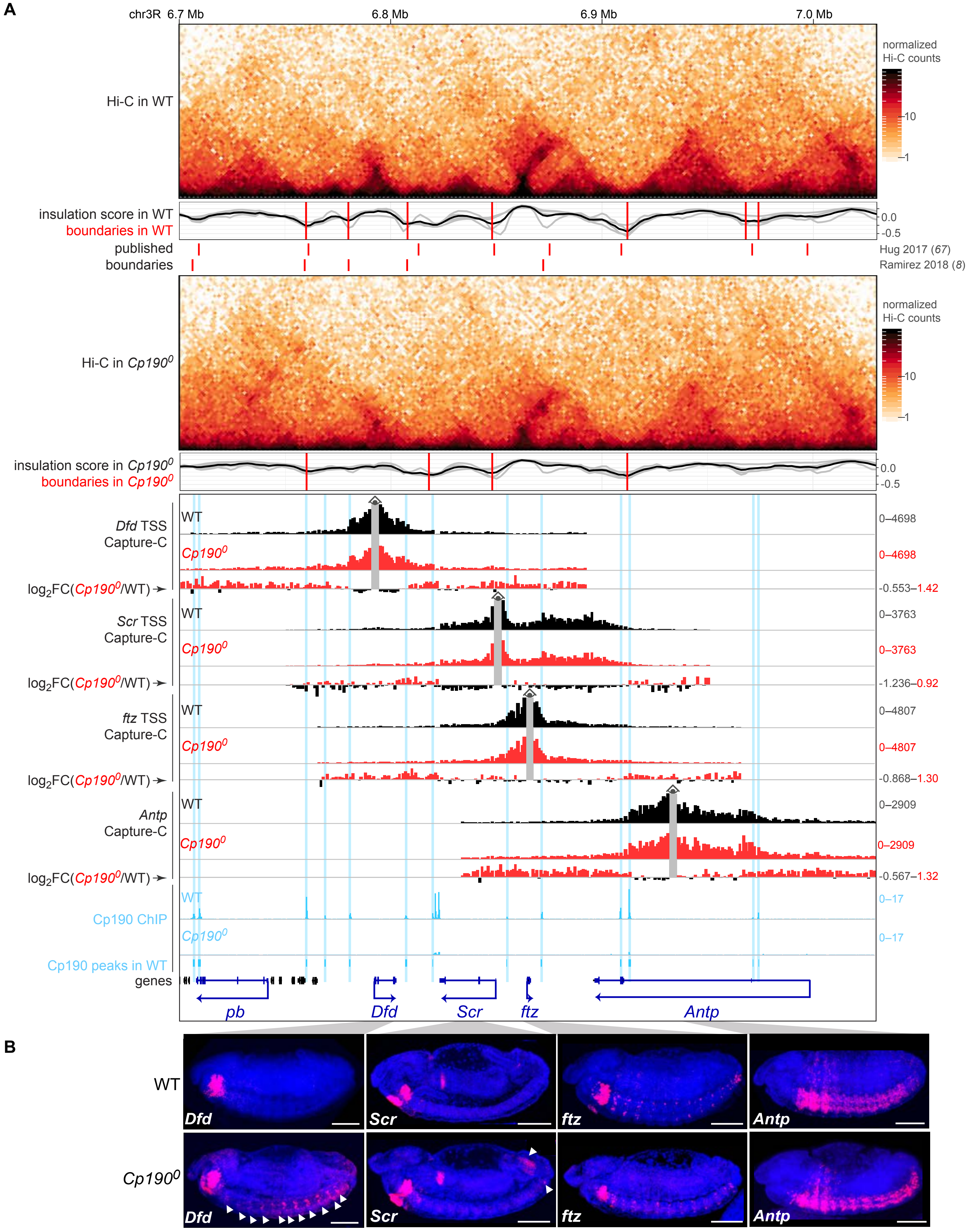

A
chr3R 6.7 Mb
6.8 Mb
6.9 Mb
7.0 Mb
Hi-C in WT
normalized Hi-C counts
10
1
insulation score in WT
boundaries in WT
0.0
-0.5
published boundaries
Hug 2017 (67)
Ramirez 2018 (8)
Hi-C in Cp190^0
insulation score in Cp190^0
boundaries in Cp190^0
Dfd TSS Capture-C
WT
Cp190^0
log2FC(Cp190^0/WT)
0–4698
-0.553–1.42
Scr TSS Capture-C
0–3763
-1.236–0.92
ftz TSS Capture-C
0–4807
-0.868–1.30
Antp Capture-C
0–2909
-0.567–1.32
Cp190 ChIP
0–17
Cp190 peaks in WT
genes
pb
Dfd
Scr
ftz
Antp
B
WT
Cp190^0
Dfd
Scr
ftz
Antp

Fig. S6. ANT-C HOX gene expression in *$Cp190^0$* mutants. Related to Figure 6.

A. Portion of the ANT-C locus (dm6 coordinates) Hi-C maps (2 kb resolution), physical insulation score (calculated with different window sizes in gray, average in black) and contact domain boundaries (vertical red lines) from this study (above) and published Hi-C studies in WT 3-4 hour old embryos [Hug et al. (*67*)] and tissue culture cells [Ramírez et al. (*8*)] (below), NG Capture-C profiles binned at 1 kb resolution around *Dfd*, *Scr*, *ftz* and *Antp* viewpoints in WT (top) and *$Cp190^0$* (bottom) embryos. Average normalized Capture-C reads (in reads per million) obtained from biological triplicates of each genotype are shown, excluding bins ±2 kb around the viewpoint (grey rectangles below eye symbols). Differences in Capture-C profiles in *$Cp190^0$* versus WT are shown as $\log_2$ fold-change profiles obtained from diffHic, with ratios >0 indicating increased contacts in *$Cp190^0$*, and values <0 indicating decreased contacts in *$Cp190^0$*. Below Capture-C profiles, Cp190 ChIP-seq in indicated genotypes (in reads per million), Cp190 peaks defined as enriched in WT relative to *$Cp190^0$* in blue, and gene tracks (only longest isoform of each protein coding gene shown, homeobox genes are blue). This shows that for each viewpoint in *$Cp190^0$* mutants, Capture-C contacts are qualitatively weakly increased in broad contiguous regions beyond former Cp190 peaks at the expense of intra-domain contacts.

B. RNA-FISH with antisense probes (red) against *Dfd*, *Scr*, *ftz* or *Antp* mRNAs in stage 14 (mid-embryogenesis) embryos stained with DAPI (blue) and imaged from the side (anterior left, posterior right, scale bars 100 µm). *Scr* and *ftz* images are reproduced from Fig. S5D for completeness. In WT embryos, *Dfd* is expressed in mandibular and maxillary segments. In *$Cp190^0$* embryos, *Dfd* is additionally misexpressed in the nervous system (arrowheads). In WT embryos, *Antp* is expressed in anterior segments and in the nerve cord. In *$Cp190^0$* embryos, *Antp* appears normally expressed.

Figure S7

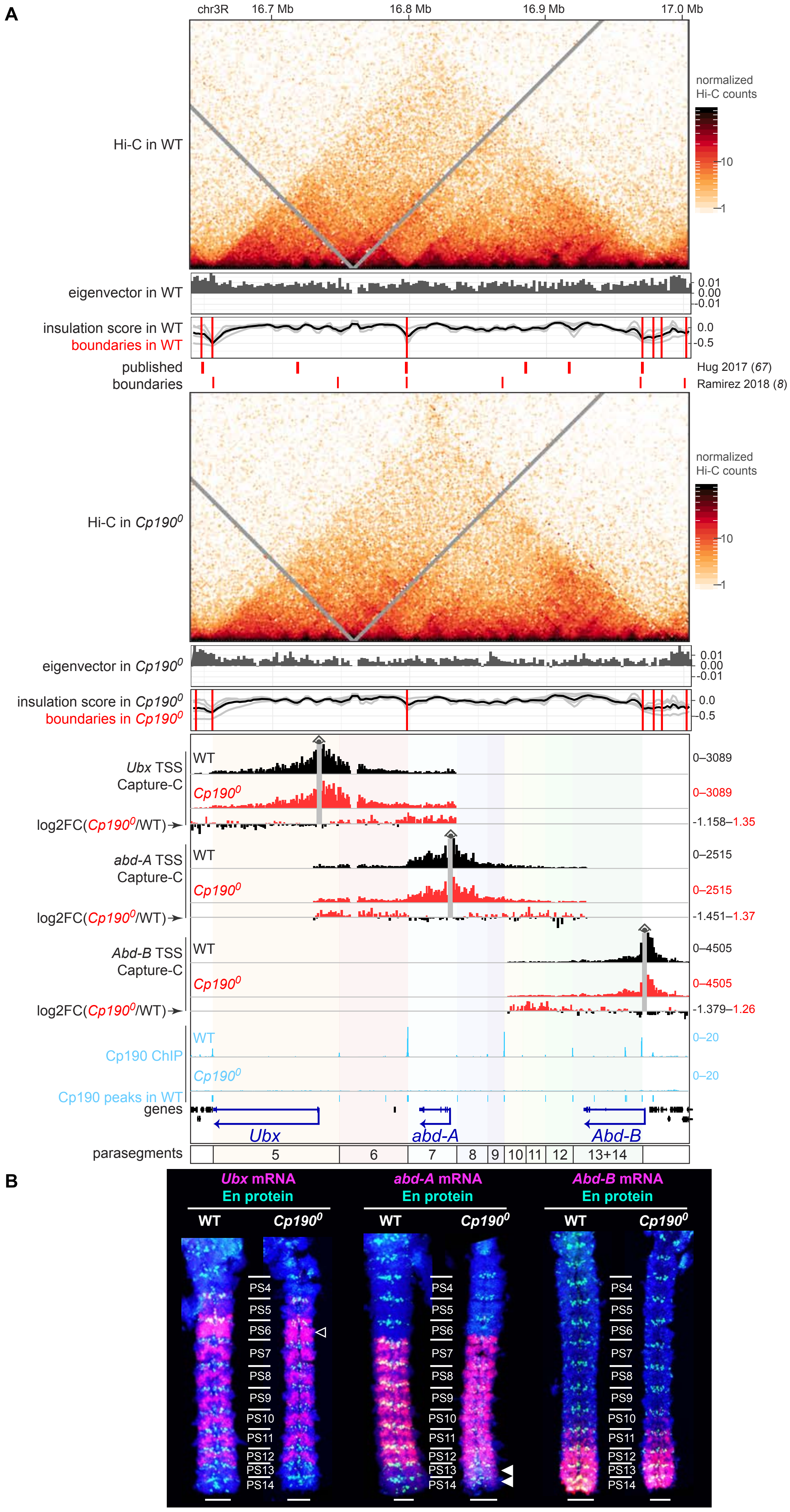
A
chr3R
16.7 Mb
16.8 Mb
16.9 Mb
17.0 Mb
Hi-C in WT
normalized Hi-C counts
10
1
eigenvector in WT
0.01
0.00
-0.01
insulation score in WT
boundaries in WT
0.0
-0.5
published boundaries
Hug 2017 (67)
Ramirez 2018 (8)
Hi-C in Cp190^0
eigenvector in Cp190^0
insulation score in Cp190^0
boundaries in Cp190^0
Ubx TSS Capture-C
WT
Cp190^0
log2FC(Cp190^0/WT)
0–3089
0–3089
-1.158–1.35
abd-A TSS Capture-C
0–2515
0–2515
-1.451–1.37
Abd-B TSS Capture-C
0–4505
0–4505
-1.379–1.26
Cp190 ChIP
0–20
0–20
Cp190 peaks in WT
genes
Ubx
abd-A
Abd-B
parasegments
5
6
7
8
9
10
11
12
13+14
B
Ubx mRNA
En protein
abd-A mRNA
Abd-B mRNA
WT
Cp190^0
PS4
PS5
PS6
PS7
PS8
PS9
PS10
PS11
PS12
PS13
PS14

Fig. S7. Cp190 is dispensable for abdominal HOX gene activation by long-range enhancers. Related to Figure 6.

A. Bithorax-complex locus (dm6 coordinates) Hi-C maps (2 kb resolution), eigenvector values (2 kb resolution, positive for A compartment, negative for B compartment), physical insulation score (calculated with different window sizes in gray, average in black) and contact domain boundaries (vertical red lines) from this study (above) and published Hi-C studies in WT 3-4 hour old embryos [Hug et al. (*67*)] and tissue culture cells [Ramírez et al. (*8*)] (below), NG Capture-C profiles binned at 1 kb resolution around *Ubx*, *abd-A* and *Abd-B* TSS viewpoints in WT (top) and *Cp190*^{0} (bottom) embryos. Average normalized Capture-C reads (in reads per million) obtained from biological triplicates of each genotype are shown, excluding bins ±2 kb around the viewpoint (grey rectangles below eye symbols). Differences in Capture-C profiles in *Cp190*^{0} versus WT are shown as $\log_2$ fold-change profiles obtained from diffHic, with ratios >0 indicating increased contacts in *Cp190*^{0}, and values <0 indicating decreased contacts in *Cp190*^{0}. Below Capture-C profiles, Cp190 ChIP-seq in indicated genotypes (in reads per million), Cp190 peaks defined as enriched in WT relative to *Cp190*^{0} in blue, and gene tracks (only longest isoform of each protein coding gene shown, homeobox genes are blue). Enhancer domains driving expression of *Ubx* (shades of orange), *abd-A* (shades of blue) and *Abd-B* (shades of green) in the indicated parasegments are colored. This shows that in *Cp190*^{0} mutants, Capture-C contacts are qualitatively weakly increased in broad contiguous regions beyond former Cp190 peaks at the expense of intra-domain contacts.

B. Ventral nerve cords dissected from stage 15 embryos (at mid-embryogenesis) (oriented with anterior up) of the indicated genotypes subjected to RNA-FISH with probes against the indicated abdominal HOX gene mRNAs (red), followed by immunostaining with anti-Engrailed (green) to mark parasegment boundaries and DAPI-labeling (blue) of DNA. Parasegments (PS) are labeled. Phenotypes consistently seen in *Cp190*^{0} mutants are marked by arrowheads: the empty arrowhead shows lower *Ubx* mRNA levels in PS6 in *Cp190*^{0} than in WT, and solid arrowheads show higher *abd-A* mRNA levels in PS13 and PS14 in *Cp190*^{0} than in WT. Scale bars below each nerve cord show 50 µm.

Figure S8

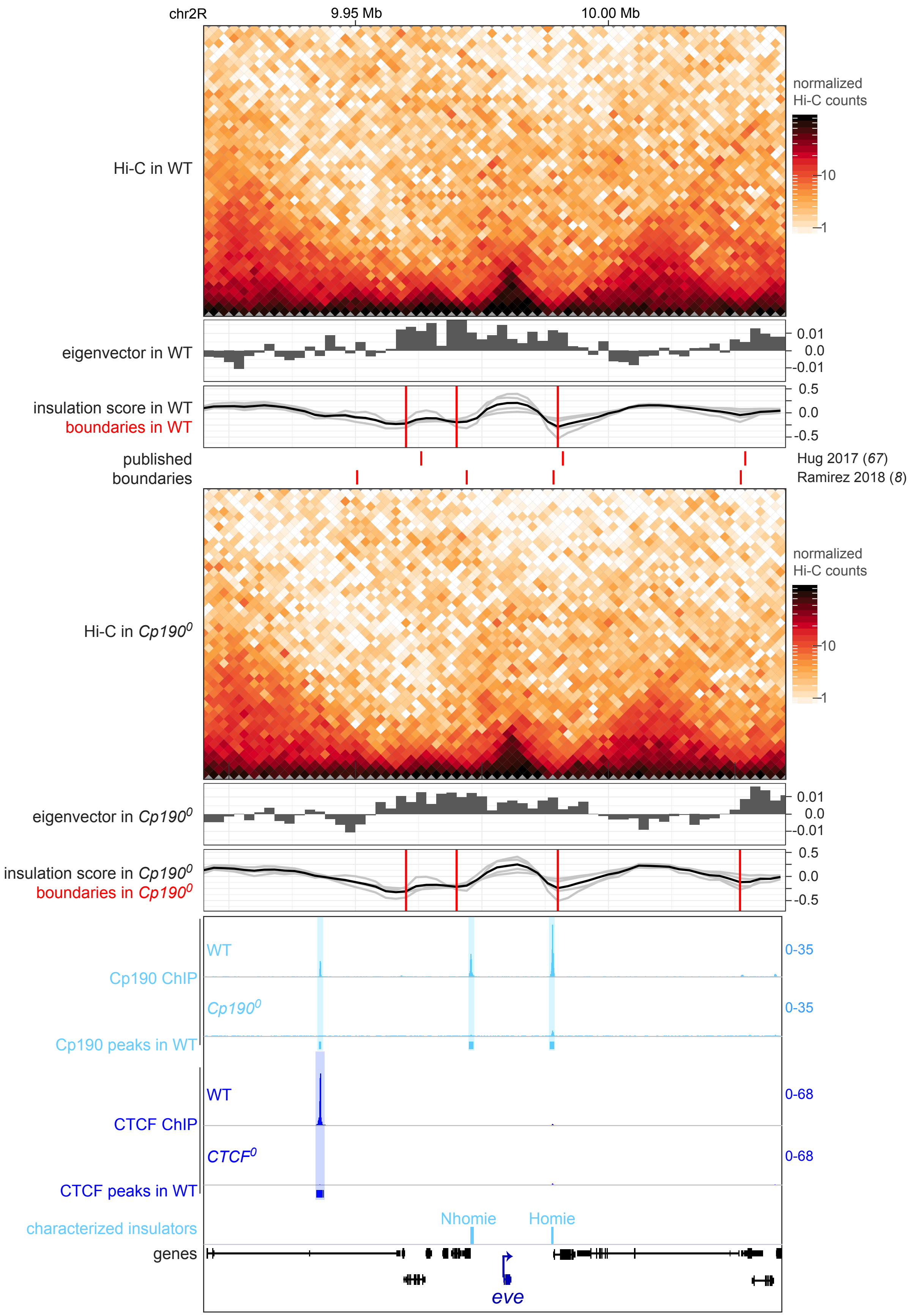

chr2R
9.95 Mb
10.00 Mb
Hi-C in WT
normalized
Hi-C counts
10
1
eigenvector in WT
0.01
0.0
-0.01
insulation score in WT
boundaries in WT
0.5
0.0
-0.5
published
boundaries
Hug 2017 (67)
Ramirez 2018 (8)
Hi-C in Cp190⁰
normalized
Hi-C counts
10
1
eigenvector in Cp190⁰
0.01
0.0
-0.01
insulation score in Cp190⁰
boundaries in Cp190⁰
0.5
0.0
-0.5
Cp190 ChIP
WT
0-35
Cp190⁰
0-35
Cp190 peaks in WT
CTCF ChIP
WT
0-68
CTCF⁰
0-68
CTCF peaks in WT
characterized insulators
Nhomie
Homie
genes
eve

Fig. S8. Homie overlaps a Cp190-occupied boundary. Related to Figure 7.

eve locus (dm6 coordinates) Hi-C maps (2 kb resolution), eigenvector values (2 kb resolution, positive for A compartment, negative for B compartment), physical insulation score (calculated with different window sizes in gray, average in black) and contact domain boundaries (vertical red lines) from this study (above) and published Hi-C studies in WT 3-4 hour old embryos [Hug et al. (*67*)] and tissue culture cells [Ramírez et al. (*8*)] (below), Cp190 ChIP-seq (in reads per million), Cp190 peaks defined as enriched in WT relative to $Cp190^0$ in light blue, CTCF ChIP-seq, CTCF peaks defined as enriched in WT relative to $CTCF^0$ in dark blue, characterized Nhomie and Homie insulators [Fujioka et al. (*37*)], and gene tracks (only longest isoform of each protein-coding gene shown, homeobox genes are blue). This shows that Homie overlaps a Cp190-occupied boundary in WT that is not visibly affected in $Cp190^0$ mutants.

Hi-C sample	total reads	interchr	intrachr	intrachr (<20 kb)	intrachr (≥20 kb)
WT_embryo_rep1234_79M	79000000	14918886	64081114	24847640	39233474
Cp190^0_embryo_rep1234_79M	79000000	20871310	58128690	22155105	35973585
CTCF^0_embryo_rep1234_79M	79000000	15880776	63119224	26388098	36731126
double^0_embryo_rep1234_79M	79000000	13048469	65951531	27630124	38321407

Table S1. Quality metrics of Hi-C reads.

Primer name	Primer sequence (5'-3')	Purpose
dmscr_1	CGAATTTATGACTGTCCATCA GCGCCAG	MUSE-RNA-FISH probe against *Scr* mRNA
dmscr_2	GCACACGCGCAATTTGTTAC AGATCACG	MUSE-RNA-FISH probe against *Scr* mRNA
dmscr_3	AGCGACGGATTTGGACGGCT TTTGGTAT	MUSE-RNA-FISH probe against *Scr* mRNA
dmscr_4	TTGCGTCCTACCTGCAGGCTT ATGTTTTG	MUSE-RNA-FISH probe against *Scr* mRNA
dmscr_5	GCGGAAATCAACGTGCACAA TTACGAAGG	MUSE-RNA-FISH probe against *Scr* mRNA
dmscr_6	GCACTGAACCACGATTTTCA CTTGTGCTGAG	MUSE-RNA-FISH probe against *Scr* mRNA
dmscr_7	GGCGTTCACCTCCGTTCAAGT GAGTTTGTTT	MUSE-RNA-FISH probe against *Scr* mRNA
dmscr_8	ATTTTAAAGCCAGGGGTCGT TGTCGTGG	MUSE-RNA-FISH probe against *Scr* mRNA
dmscr_9	GAACTGGTACGAGGACATCG CAAAACAG	MUSE-RNA-FISH probe against *Scr* mRNA
dmscr_10	TCGTTGGCGTACTTGCAACTG ATGTTCG	MUSE-RNA-FISH probe against *Scr* mRNA
dmscr_11	TTCTTTCCATTGCCGCTGTTC TGGCTACTG	MUSE-RNA-FISH probe against *Scr* mRNA
dmscr_12	TTGTCGTTTCGTCTCGCCATT GGCATTC	MUSE-RNA-FISH probe against *Scr* mRNA
dmscr_13	AGTCTCTAACCAGTACCCGA AAAGTGCC	MUSE-RNA-FISH probe against *Scr* mRNA
dmscr_14	GAGATCAGAACTCCTGCGGA TACTTGATG	MUSE-RNA-FISH probe against *Scr* mRNA
dmscr_15	AGTTTTAGGTCCCGCTCCTGA TTCCGAT	MUSE-RNA-FISH probe against *Scr* mRNA
dmscr_16	TTCGACAATGTCCGCCTTGA ACTCCAGTT	MUSE-RNA-FISH probe against *Scr* mRNA
dmscr_17	TTAAAGAGTGACGTCGATTG CTCGTGGTTCC	MUSE-RNA-FISH probe against *Scr* mRNA
dmscr_18	CCCTCGAGCATTCGCATAGA AAGGTTTAG	MUSE-RNA-FISH probe against *Scr* mRNA
dmscr_19	CTGCTCATCGTGCAGCTTACG TGCTAAAA	MUSE-RNA-FISH probe against *Scr* mRNA
dmscr_20	GTTGGAGATTGGGCGATACA AACGAAGAC	MUSE-RNA-FISH probe against *Scr* mRNA
dmftz_1	CTTCTCTAACTCTGCGATGTG CACGCAACG	MUSE-RNA-FISH probe against *ftz* mRNA

dmftz_2	TTGCTGCCTGAATTATCGTAG TAGGTGG	MUSE-RNA-FISH probe against *ftz* mRNA
dmftz_3	GCCCTGATAATTGGAGGTGT TCTGATAG	MUSE-RNA-FISH probe against *ftz* mRNA
dmftz_4	AGTAGCAGCTCTCCGAGTAA CTCTCCTG	MUSE-RNA-FISH probe against *ftz* mRNA
dmftz_5	AAGCAGCATCATCTTCGGCC TTGCGCTT	MUSE-RNA-FISH probe against *ftz* mRNA
dmftz_6	CTTCAGCTTCTTCACGGGATT GGTGAGC	MUSE-RNA-FISH probe against *ftz* mRNA
dmftz_7	CTGCTCGACGGTTGTGTAGA AATAGTCGG	MUSE-RNA-FISH probe against *ftz* mRNA
dmftz_8	ATTCGATGATTGATCTCCTGG CTGACAG	MUSE-RNA-FISH probe against *ftz* mRNA
dmftz_9	CTCCTCGATGTGCGACCAATT GAAATCG	MUSE-RNA-FISH probe against *ftz* mRNA
dmftz_10	GCGTTTCGAGTCTTTGCAATC TGATGCCAAAG	MUSE-RNA-FISH probe against *ftz* mRNA
dmftz_11	TCTTGATCTGCCTTTCGCTCA GGCTCAG	MUSE-RNA-FISH probe against *ftz* mRNA
dmftz_12	CTCTGGGGAAGAGAGTAACT GAGCATCG	MUSE-RNA-FISH probe against *ftz* mRNA
dmftz_13	AATGGTCGAGAGAAGTGCGC TTCGGTTTCGT	MUSE-RNA-FISH probe against *ftz* mRNA
dmftz_14	CTTCGTTCTCGGCTGTGTCAT TTGCGTG	MUSE-RNA-FISH probe against *ftz* mRNA
dmftz_15	TCTTTTGCCTCTGCCTTCTGC ACTTGCG	MUSE-RNA-FISH probe against *ftz* mRNA
dmftz_16	AGCAAGGCTCCTTTTCTGTTT GCGCTGC	MUSE-RNA-FISH probe against *ftz* mRNA
dmftz_17	AGCTAATCGATCGCTGAGAA CCCATC	MUSE-RNA-FISH probe against *ftz* mRNA
dmftz_18	AGCTGAGTGTTTTGGGCTTGT GTTTGGC	MUSE-RNA-FISH probe against *ftz* mRNA
CTCF A fw	CTCCGGAATATTAGGTCTCAT ACTTTTCATTTCATTAATTTG CGG	CTCF-bound insulator cloning (Fig. 4b)
CTCF A rv	GGCTCAAGCAGTGGGTCTCC CATTTTTTGGTTCATATGAAG CGC	CTCF-bound insulator cloning (Fig. 4b)
CTCF B fw	CTCCGGAATATTAGGTCTCAT ACTGCATCATTTTGTAGTTGT CC	CTCF-bound insulator cloning (Fig. 4b)

CTCF B rv	GGCTCAAGCAGTGGGTCTCCCATTAATTGGGAAATAAACTCTAGC	CTCF-bound insulator cloning (Fig. 4b)
Su(Hw) A' fw	CTCCGGAATATTAGGTCTCATACTAGCCCTGCAACTCAATGG	Su(Hw)-bound insulator cloning (Fig. 4b)
Su(Hw) A' rv	GGCTCAAGCAGTGGGTCTCCCATTATGCCAATTGACTGCATGG	Su(Hw)-bound insulator cloning (Fig. 4b)
Su(Hw) B' fw	CTCCGGAATATTAGGTCTCATACTTTGGGGATTGTTTGTAATGTTG	Su(Hw)-bound insulator cloning (Fig. 4b)
Su(Hw) B' rv	GGCTCAAGCAGTGGGTCTCCCATTTGCTGGCAACATTTTAGTGG	Su(Hw)-bound insulator cloning (Fig. 4b)
Su(Hw) C' fw	CTCCGGAATATTAGGTCTCATACTGGTTTTACATTTGACTTCTTCG	Su(Hw)-bound insulator cloning (Fig. 4b)
Su(Hw) C' rv	GGCTCAAGCAGTGGGTCTCCCATTTGATCGGCTAAGTGAAGTGG	Su(Hw)-bound insulator cloning (Fig. 4b)
Su(Hw) D' fw	CTCCGGAATATTAGGTCTCATACTCGCACTTCCCCACCAACC	Su(Hw)-bound insulator cloning (Fig. 4b)
Su(Hw) D' rv	GGCTCAAGCAGTGGGTCTCCCATTGCTGGCGGCATACAAATAG	Su(Hw)-bound insulator cloning (Fig. 4b)
Chro/Pzg/BEAF A'' fw	CTCCGGAATATTAGGTCTCATACTGTCACAGGATTGCTGGTGG	Chro/Pzg/BEAF-3 insulator cloning (Fig. 4b)
Chro/Pzg/BEAF A'' rv	GGCTCAAGCAGTGGGTCTCCCATTAATTGTGTTTCACTGCTTTGC	Chro/Pzg/BEAF-3 insulator cloning (Fig. 4b)
Chro/Pzg/BEAF B'' fw	CTCCGGAATATTAGGTCTCATACTAACAACAAGCGCATATGTTTGG	Chro/Pzg/BEAF-3 insulator cloning (Fig. 4b)
Chro/Pzg/BEAF B'' rv	GGCTCAAGCAGTGGGTCTCCCATTAGCGAATAATTTAATTCACACC	Chro/Pzg/BEAF-3 insulator cloning (Fig. 4b)
Chro/Pzg/BEAF C'' fw	CTCCGGAATATTAGGTCTCATACTTCCGAAAATTTCTTCTCTGACC	Chro/Pzg/BEAF-3 insulator cloning (Fig. 4b)

Chro/Pzg/BEAF C" rv	GGCTCAAGCAGTGGGTCTCCCATTCAAGAAAGTATATAAAATGCGC	Chro/Pzg/BEAF-3 insulator cloning (Fig. 4b)
Chro/Pzg/BEAF D" fw	CTCCGGAATATTAGGTCTCATACTGCTGTTGTTGTTCGAAATGTTG	Chro/Pzg/BEAF-3 insulator cloning (Fig. 4b)
Chro/Pzg/BEAF D" rv	GGCTCAAGCAGTGGGTCTCCCATTCACACTTGCACTTAGACGCGGC	Chro/Pzg/BEAF-3 insulator cloning (Fig. 4b)
Chro/Pzg/BEAF E" fw	CTCCGGAATATTAGGTCTCATACTGACAAAACTGCCACAAAATC	Chro/Pzg/BEAF-3 insulator cloning (Fig. 4b)
Chro/Pzg/BEAF E" rv	GGCTCAAGCAGTGGGTCTCCCATTGAAATGTAATGCATTCGACTC	Chro/Pzg/BEAF-3 insulator cloning (Fig. 4b)
Chro/Pzg/BEAF F" fw	CTCCGGAATATTAGGTCTCATACTAACGTTTTGCCGGCTACGC	Chro/Pzg/BEAF-3 insulator cloning (Fig. 4b)
Chro/Pzg/BEAF F" rv	GGCTCAAGCAGTGGGTCTCCCATTGGCCATGTTAAAGTCTGTG	Chro/Pzg/BEAF-3 insulator cloning (Fig. 4b)
Chro/Pzg/BEAF G" fw	CTCCGGAATATTAGGTCTCATACTTGTTCTATCCTCTTCCTTGG	Chro/Pzg/BEAF-3 insulator cloning (Fig. 4b)
Chro/Pzg/BEAF G" rv	GGCTCAAGCAGTGGGTCTCCCATTGCCCAACTCGACGCTAGC	Chro/Pzg/BEAF-3 insulator cloning (Fig. 4b)
Chro/Pzg/BEAF H" fw	CTCCGGAATATTAGGTCTCATACTCATGTTTAACCAACTTACCTAG	Chro/Pzg/BEAF-3 insulator cloning (Fig. 4b)
Chro/Pzg/BEAF H" rv	GGCTCAAGCAGTGGGTCTCCCATTGGCACAAAACAATGGCATTTG	Chro/Pzg/BEAF-3 insulator cloning (Fig. 4b)
Chro/Pzg/BEAF I" fw	CTCCGGAATATTAGGTCTCATACTACTAGCAACAAGACGCAC	Chro/Pzg/BEAF-3 insulator cloning (Fig. 4b)
Chro/Pzg/BEAF I" rv	GGCTCAAGCAGTGGGTCTCCCATTATGGCTTTCGCTTGTTGATTCC	Chro/Pzg/BEAF-3 insulator cloning (Fig. 4b)
Chro/Pzg/BEAF J" fw	CTCCGGAATATTAGGTCTCATACTGCTCCAAAATTTCCCGCTCG	Chro/Pzg/BEAF-3 insulator cloning (Fig. 4b)

Chro/Pzg/BEAF J" rv	GGCTCAAGCAGTGGGTCTCC CATTAACAAATTTTCCCTGCA TAG	Chro/Pzg/BEAF-3 insulator cloning (Fig. 4b)
Chro/Pzg/BEAF K" fw	CTCCGGAATATTAGGTCTCAT ACTCCGCTGCTTATACGCACC	Chro/Pzg/BEAF-3 insulator cloning (Fig. 4b)
Chro/Pzg/BEAF K" rv	GGCTCAAGCAGTGGGTCTCC CATTTACCTTCAGTACTTAAT GTTCC	Chro/Pzg/BEAF-3 insulator cloning (Fig. 4b)
Chro/Pzg/BEAF L" fw	CTCCGGAATATTAGGTCTCAT ACTTCTGCAGTGCTCGAAAG TGTC	Chro/Pzg/BEAF-3 insulator cloning (Fig. 4b)
Chro/Pzg/BEAF L" rv	GGCTCAAGCAGTGGGTCTCC CATTGGCGAATTTGCGTGAA ATTG	Chro/Pzg/BEAF-3 insulator cloning (Fig. 4b)
Chro/Pzg/BEAF M" fw	CTCCGGAATATTAGGTCTCAT ACTGAATGCACACTTGCAAT TTGCG	Chro/Pzg/BEAF-3 insulator cloning (Fig. 4b)
Chro/Pzg/BEAF M" rv	GGCTCAAGCAGTGGGTCTCC CATTGCGGCTGACGCCTTAT AAAC	Chro/Pzg/BEAF-3 insulator cloning (Fig. 4b)
Chro/Pzg/BEAF B" mut1 fw	CGATAAGCCAAACATATGC	Chro/Pzg/BEAF-3 insulator cloning (Fig. 4b)
Chro/Pzg/BEAF B" mut1 rv	CTAGTCTGGAGCTATTGCAG	Chro/Pzg/BEAF-3 insulator cloning (Fig. 4b)
Chro/Pzg/BEAF B" mut2 fw	CGATTGTTTTGTTGAAAAGG	Chro/Pzg/BEAF-3 insulator cloning (Fig. 4b)
Chro/Pzg/BEAF B" mut2 rv	CTATTACCTCACCGCCTAAA AC	Chro/Pzg/BEAF-3 insulator cloning (Fig. 4b)
Chro/Pzg/BEAF F" mut1 fw	CGATATTTAATTGCGTAGCC	Chro/Pzg/BEAF-3 insulator cloning (Fig. 4b)
Chro/Pzg/BEAF F" mut1 rv	CTAACACCACTGTCATACC	Chro/Pzg/BEAF-3 insulator cloning (Fig. 4b)
Chro/Pzg/BEAF F" mut2 fw	CGATTGTTTGTCACATGGATT G	Chro/Pzg/BEAF-3 insulator cloning (Fig. 4b)
Chro/Pzg/BEAF F" mut2 rv	CTAGACCGCTGCTGCCCCAC	Chro/Pzg/BEAF-3 insulator cloning (Fig. 4b)
su(Hw) L sgRNA fw	GTCGCCTTGGAGGCACTCAT GT	su(Hw) sgRNA left
su(Hw) L sgRNA rv	AAACACATGAGTGCCTCCAA GG	su(Hw) sgRNA left
su(Hw) R sgRNA fw	GTCGGTACGCATGCATCAAG GGC	su(Hw) sgRNA right

su(Hw) R sgRNA rv	AAACGCCCTTGATGCATGCGTAC	su(Hw) sgRNA right
su(Hw) LHA fw	GTCGACGAATTCTCCGCCTCCTGTTTGTAATC	su(Hw) homology arm left
su(Hw) LHA rv	GTCGACgcggccgcTGTTGGTGATACCAGCctg	su(Hw) homology arm left
su(Hw) RHA fw	GTCGACACGCGTCTTGATGCATGCGTACAATTACC	su(Hw) homology arm right
su(Hw) RHA rv	gagaGCTCTTCgGACAGCCAGTCTATGTCGCCAGT	su(Hw) homology arm right
SF1 L sgRNA fw	gtcgGTCCTTCTAACAGGTTCT	SF1 sgRNA left
SF1 L sgRNA rv	AAACAGAACCTGTTAGAAGGAC	SF1 sgRNA left
SF1 R sgRNA fw	GTCGCTGAAATCAGCATAAAAGG	SF1 sgRNA right
SF1 R sgRNA rv	AAACCCTTTTATGCTGATTTCAG	SF1 sgRNA right
SF2b L sgRNA fw	GTCGGTTTGGCTCGGAGATTTCA	SF2b sgRNA left
SF2b L sgRNA rv	AAACTGAAATCTCCGAGCCAAAC	SF2b sgRNA left
SF2b R sgRNA fw	GTCGGCAAGTAAACAGAAATATC	SF2b sgRNA right
SF2b R sgRNA rv	AAACGATATTTCTGTTTACTTGC	SF2b sgRNA right
SF1 LHA fw	GAGACACCTGCGAGATCGCGGCCTTGGAGGTCATTAGG	SF1 homology arm left
SF1 LHA rv	GAGACACCTGCGAGACTACACCTGTTAGAAGGACATTCAG	SF1 homology arm left
SF1 RHA fw	ccccagttggggcactacGCTCTTCGTATAGGTGGGATTGCAGACAGC	SF1 homology arm right
SF1 RHA rv	GTCGCCCTTGAACTCGATTGCTCTTCGGACAAATGCAATAACTGGGAGTGG	SF1 homology arm right
SF2b LHA fw	GAGACACCTGCGAGATCGCCCGTGCCGTTTTTATAGCC	SF2b homology arm left
SF2b LHA rv	GAGACACCTGCGAGACTACTCATGGAAAGTACAAGAAAC	SF2b homology arm left
SF2b RHA fw	ccccagttggggcactacGCTCTTCGTATATCTGGAAGACACAATTATAC	SF2b homology arm right

SF2b RHA rv	GTCGCCCTTGAACTCGATTGCTCTTCGGACTGCATGTGCGATCTGAAGTT	SF2b homology arm right
Scr fw	ATGGATCCCGACTGTTTTGCG	Scr RNA-FISH probe
T7 Scr rv	AATACGACTCACTATAGGTGCGCTTAGGTGCGCGAACTGC	Scr RNA-FISH probe
ftz fw	ATTCGCAAACTCACCAGCGT	ftz RNA-FISH probe
T7 ftz rv	AATACGACTCACTATAGGGATGGTAGAGGTCCTGTGG	ftz RNA-FISH probe
Dfd fw	ATGAGCTCTTTTCTCATGGG	Dfd RNA-FISH probe
T7 Dfd rv	GAAATTAATACGACTCACTATAGGCAGGGCCGTCAGATCGTAG	Dfd RNA-FISH probe
abd-A fw	TTCGCCGTGTTTATTGTTCC	abd-A RNA-FISH probe
T7 abd-A rv	GAAATTAATACGACTCACTATAGGAACAAGGCAAAAGGTTGTCG	abd-A RNA-FISH probe

Table S2. Primer list.
List of primers used.

Taf1_TSS_L	GTACAAAACGCAATTGACCTTCCCAAGCTCCCAACCCTTGTCC AAATCTAATCGCGGATTTTCTGGTGCCAGTGGGGAGCCATGAG GCATCTTTGCTTTGTCCATCGACGGTCACACTTA
Taf1_TSS_R	GTACTCACTTGGAAAGGGAACCAATGTTTTCCCGGAGCTCCGC ATCGAAACCGGTGCCCCCGCGCCCCTCTCCGTCGTCATCTTGCA GCAGTCTGCCCTCGGAGTCGATGTTGCCGAAAA
Dfd_TSS_L	GTACGAGGCGAAGGGTGAGAAAAGGTGAACAGACATGGCTGC TACATATGAATGGCTTTATTTGTGCGCGTGTGTGGGCCGTCCTT CGATTGCTTCGGCAGTGAAAACCCGTTTTGATGG
Dfd_TSS_R	TTAAAAGCCGTGGGGCCTTGGAATTCTGAGAAACTTGATTATG GTGTTATTTTCCGGTTATAAAAAACGTTTTACAACGAATTTCA ATCCCACACAGGGAGAAAAGTCGGACGTTTCGG
Scr_TSS_L	GTACGAATACGAGAACGTGTATCTGTATCTTTTTATGTTTGCAG AAGACCGAGCGCGCTGCTTGGCGACTGAAGTGCGCCACGTTCA ATTCACGTTTGAGCCATAAAAACCGACAAAACG
Scr_TSS_R	TTAAGCTTCAGCAGGAGACTTTGCATTTTGCCAGGCCAATAAA AATGGAAATGAACGCGGAGCGGAACGTTTATAAAATAATAAT GCGAAATAATAATGTGGCGGTGATTTAGCGGAACA
ftz_TSS_L	GTACGTGACTGTGCCCACGCCCAGCGCCTCCGAGGATGTCGAC TACTTGGACGTCTACTCGCCCCAGTCGCAGACGCAGAAGCTGA AGAATGGCGACTTTGCCACCCCTCCGCCAACCAC
ftz_TSS_R	TTAATCGTGTGTGATGCCTACCTGATGCCAAAGTCTCCTCGATG TGCGACCAATTGAAATCGCCGGCTCCATTCGGGGCTGTCACAA TTCGATGATTGATCTCCTGGCTGACAGCTGACG
Antp_TSS_L	TTAAGAACTGGGAATGAACCGAACGGAACTAAACGCGTGAAA CGACGGCAAAACCGAAAGCAAAGTGCGAATCGAGCGGCGGCG GCACGTCTATTTGGCACGTCCATTCATTCACAACTG
Antp_TSS_R	TTAACGAGCCACTAGTTACCTATCCAGCACTGTTTGCGTTCTCC AGCCGGCGAGAGTGTGTGTGTGTTTGCACAAAAACAATGCCGC CATTTATCATTTTCTATGGTGGCCGTGTTGCTG
ArgRS-m_TSS_L	TTAAAAACAGAAACTTGCGCGAGAGCCACGCTCAGATTCTTAT TTTCTTGCCCTAGCCAATCGATATCTATTTTTCATGACAGCCCC GTGGTATGCAATACTTTTTAGCCGCTTCGCGCT
ArgRS-m_TSS_R	TTAAATTTGCAGACCTAACCTTCTTGCCCAAAAATTTCCCAAGT ATCTAAAAGCTTGTTTTGCCTATTGGTAACGCAGTTTCGTGCAC CCACCGACGCCCATGCAAACAGATGTTCAGTG
stck_TSS_L	TTAAAAAGAAACTCATTTCATAGTGATTGTGGGTTTCATAGCA AGTTTACAAGCTCTTGATTCTCTTATCAATACATATTTTCTAAT GATAAGTATCAGCGAAACTGTTATGACATTTGT
stck_TSS_R	GTACTATTTGTTTGTAAACAAGACCGGTTCGCATGCCAATTTAG GCTGCAGGCAAGAAATCCCTTTAGCATTGAATTTGATTGATTTT CTTTTTGCTATGTTACACGCCAATTGGGGAAA
Ubx_TSS_L	TTAACGATTTGAACGATTATTAGCCATAGTGCTGACCGAACGG GCGCGAATGCGTCTGCTGACGGATTTCCTCGAATCTGGACGCC AATCTAGTGGAAAGCGACTTTTCCGCGCGGCATT
Ubx_TSS_R	TTAAACTGAACGAACACTCAAGAGAGAGCGCAAGAGCGCTCA AAAACAATCTGGTTTTGAGCGTTTCGCTGGCTCTCTGTTTCTGT TTTCCACTCGTTTTTAGGCCGAGTCGAGTGAGTT

abd-A_TSS_L	TTAAAAGAGTATAAAAATTTCGTGTAATTGGTAATTCTTGCTGCCAGCGCGGGCAGCGGCGTCGACAGAAACGGCGGCAGAGCGGCAGCGACTGAGGCGCTTTGAGTCGTTGGAGACTTT
abd-A_TSS_R	TTAAAAAGCATAGCACTCAAAGCGGGGCTCCAATAGTTTCCATTCTCACGTTATAAAGAGTGAAATTAGGAGAGGCACTCAAATCGGGAAATTACTCACTCAAAGCAGCGAGGCGGCCAT
Abd-B_TSS_L	TTAACACTTTCGAGCAAGAGCGCGCCGGCGGTGGCCAAGTGTTAGAGCCAGTTTTTGGTTTTGGAGTCCGAGGTGCGGAGCGTCATCGAGTGAACTGAATGGTGTGCGAGTTCTCCGCTG
Abd-B_TSS_R	TTAAGGGTGCGGGTGCGAGTCGAAAAAAAAGATATATGCCCGTCATATATCTAGGCGGTTCCCCATGCCGCTTTATTTATTTGTTTATCTACTATTTATGGTCCCAAGAGCCGAAGCCGA
Blocking oligo 1	CAAGCAGAAGACGGCATACGAGATCGGTCTCGGCATTCCTGCTGAACCGCTCTTCCGATCT
Blocking oligo 2	AATGATACGGCGACCACCGAGATCTACACTCTTTCCCTACACGACGCTCTTCCGATCT

Table S3. Capture-C probe list and blocking oligos.
List of probes and blocking oligos used for Capture-C.

Capture-C sample	total read pairs	interchr	intrachr	intrachr (<20 kb)	intrachr (≥20 kb)
WT_embryo_rep1	222071	35016	187055	65749	121306
WT_embryo_rep2	218013	37884	180129	58335	121794
WT_embryo_rep3	372540	81031	291509	107001	184508
$Cp190^0$_embryo_rep1	263835	35939	227896	76655	151241
$Cp190^0$_embryo_rep2	255505	43570	211935	63822	148113
$Cp190^0$_embryo_rep3	352172	25730	326442	143762	182680
$SF1^{KO}$_embryo_rep1	342434	55638	286796	99181	187615
$SF1^{KO}$_embryo_rep2	281072	44945	236127	81849	154278
$SF1^{KO}$_embryo_rep3	389964	68287	321677	107680	213997

Table S4. Quality metrics of Capture-C read pairs.

Only informative read pairs were considered, i.e. only unique read pairs (after discarding probable PCR duplicates) with at least one read mapping to a viewpoint restriction fragment or one of its 2 neighboring restriction fragments.

Data_S1_ChIPseq_Cp190_WT0-Cp1900_embryo.xlsx
Differential Cp190 ChIP-seq analysis in wildtype versus *Cp190*^{0} embryos (Cp190 peaks in WT embryos).

Data S2. Hi-C_CD_boundaries.xlsx
Contact domain boundaries found in WT (column exist.WT_0), *double*^{0} (column exist.CTCF_0_Cp190_0), *CTCF*^{0} (column exist.CTCF_0) and *Cp190*^{0} (column exist.Cp190_0), with physical insulation scores measured at each boundaries in each genotype (columns score.).

Data S3. Hi-C_insulation_scores.xlsx
Physical insulation scores measured for all bins in the genome by Hi-C in WT (score.WT_0), *double*^{0} (column score.CTCF_0_Cp190_0), *CTCF*^{0} (column score.CTCF_0) and *Cp190*^{0} (column score.Cp190_0) embryos.

Data S4. Hi-C_eigenvectors.xlsx
First eigenvector and A/B compartments.

Data S5. ChIPseq_CTCF_WT0-CTCF0_embryo.xlsx
Differential CTCF ChIP-seq analysis in wildtype versus *CTCF*^{0} embryos (CTCF peaks in WT embryos). Contains only peaks in up direction on chr2,3,4 and X.

Data S6. ChIPseq_Cp190_CTCF0-Cp1900_embryo.xlsx
Differential Cp190 ChIP-seq analysis in *CTCF*^{0} versus *Cp190*^{0} embryos (Cp190 peaks in *CTCF*^{0} embryos). Contains only peaks in up direction on chr2,3,4 and X.

Data S7. ChIPseq_Cp190_CTCF0-WT0_embryo.xlsx
Differential Cp190 ChIP-seq analysis in *CTCF*^{0} versus WT embryos (differential Cp190-bound regions in *CTCF*^{0} versus WT embryos). Contains only differentially bound regions on chr2,3,4 and X.

Data S8. ChIPseq_CTCF_Cp1900-CTCF0_embryo.xlsx
Differential CTCF ChIP-seq analysis in *Cp190*^{0} versus *CTCF*^{0} embryos (CTCF peaks in *Cp190*^{0} embryos). Contains only peaks in up direction on chr2,3,4 and X.

Data S9. ChIPseq_CTCF_Cp1900-WT0_embryo.xlsx
Differential CTCF ChIP-seq analysis in *Cp190*^{0} versus WT embryos (differential CTCF-bound regions in *Cp190*^{0} versus WT embryos). Contains only differentially bound regions on chr2,3,4 and X.

Data S10. ChIPseq_Cp190_WT-Cp190KO_larva.xlsx
Differential Cp190 ChIP-seq analysis in wildtype versus *Cp190*^{KO} larval central nervous systems (Cp190 peaks in WT larval central nervous systems). Contains only peaks in up direction on chr2,3,4 and X.

Data S11. ChIPseq_Cp190_SuHwKO-Cp190KO_larva.xlsx
Differential Cp190 ChIP-seq analysis in *su(Hw)*^{KO} versus *Cp190*^{KO} larval central nervous systems (Cp190 peaks in *su(Hw)*^{KO} larval central nervous systems). Contains only peaks in up direction on chr2,3,4 and X.

Data S12. ChIPseq_Cp190_SuHwKO-WT_larva.xlsx
Differential Cp190 ChIP-seq analysis in *su(Hw)*^{KO} versus WT larval central nervous systems (differential Cp190-bound regions in *su(Hw)*^{KO} versus WT larval central nervous systems). Contains only differentially bound regions on chr2,3,4 and X.

Data S13. IBAQ.xlsx
List of intensity-based absolute quantification (IBAQ) values of proteins detected by mass spectrometry in pull-down experiments with baits shown in Fig. 4A and the respective negative control pull-downs performed in parallel.

Data S14. CaptureC_Cp1900-WT0_embryo.xlsx
Differential Capture-C analysis in *Cp190*^{0} versus WT embryos.

Data S15. CaptureC_SF1KO-WT0_embryo.xlsx
Differential Capture-C analysis in *SF1*^{KO} versus WT embryos.
